# Intermolecular Oxidopyrylium (5 + 2) Cycloaddition/Reductive Ring-Opening Strategy for the Synthesis of α-Methoxytropones

**DOI:** 10.1021/acs.joc.4c01989

**Published:** 2024-11-12

**Authors:** Orugbani S. Eli, Lauren P. Bejcek, Anastasiya Lyubimova, Dan L. Sackett, Ryan P. Murelli

**Affiliations:** † Department of Chemistry, Brooklyn College, The City University of New York, Brooklyn, New York 11210, United States; ⊥ Ph.D. Program in Chemistry, The Graduate Center of The City University of New York, New York, New York 10016, United States; § Division of Basic and Translational Biophysics, Eunice Kennedy Shriver National Institute of Child Health and Human Development, National Institutes of Health, Bethesda, Maryland 20892, United States; ∥ Ph.D. Program in Biochemistry, The Graduate Center of The City University of New York, New York, New York 10016, United States

## Abstract

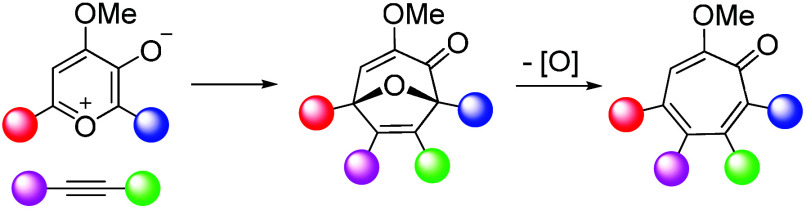

α-Methoxytropone is a structural motif found in various natural products and other compounds of interest to the scientific community but remains a synthetic challenge. The present Note describes the synthesis of variously substituted α-methoxytropones and related compounds through an intermolecular 3-hydroxy-4-pyrone-based oxidopyrylium (5 + 2) cycloaddition followed by a samarium iodide-mediated reductive ring-opening. The strategy is highlighted in the synthesis of a novel AC-ring analogue of colchicine to compare it to existing methods.

α-Methoxytropone is a structural motif found in a number of natural products and other targets of interest to the scientific community. They are also challenging synthetic targets, which limits the breadth of studies that can be carried out on them.^1^ The most famous example of an α-methoxytropone-containing molecule is the natural product colchicine (**1**) (Figure 1),^2^ a tubulin inhibitor used clinically for the treatment of inflammation-related diseases. Another example is imerubrine (**2**),^3^ an extract from the plant *Abuta imene* that has been synthesized by a number of research laboratories^4^ and that could be useful as a bradykinin receptor agonist.^5^ α-Methoxytropones may also represent interesting photocapture agents, as was recently illustrated by the ability of PF-06670015 (**3**) to photolabel bromodomains.^6^ For these reasons, strategies for the synthesis of diversely functionalized α-methoxytropones could have broad utility.

**Figure 1:**
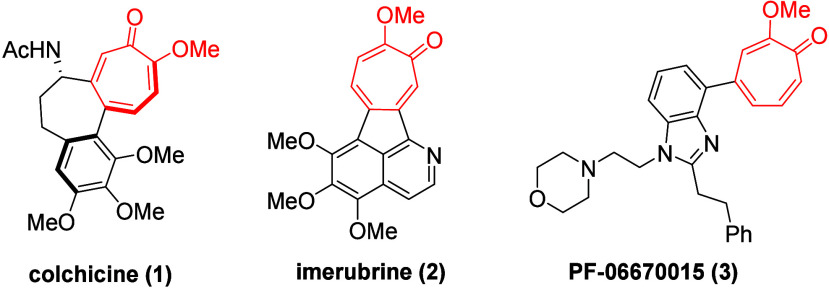
Examples of α-methoxytropolone-containing molecules with novel functions.

Our lab has published a number of papers over the last several years detailing the value of the intermolecular 3-hydroxy-4-pyrone-based oxidopyrylium (5 + 2) cycloaddition reaction in the synthesis and study of α-hydroxytropolones (αHTs) (**7**) (Scheme 1A).^7^ This strategy leverages acid-mediated ring-opening conditions and has been used in numerous medicinal chemistry studies, both by our lab^8^ and others.^9^ Our work on this scaffold has led to hundreds of diversely substituted αHTs and the identification of highly potent and selective antiviral agents against hepatitis B^10^ and herpes simplex virus.^11^

**Scheme 1:**
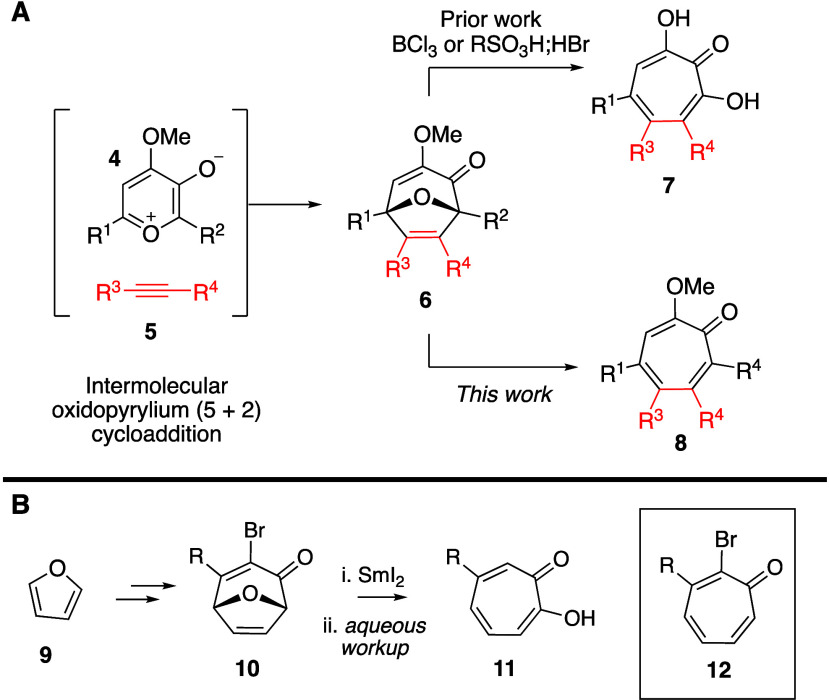
Synthetic Routes to Troponoids: (A) Graphical Comparison of Prior Acid-Mediated Work and Current Work; (B) Previously Published Samarium Iodide Route to Tropolone **11** and Side Product **12**

Given the value of this intermolecular 3-hydroxy-4-pyrone-based oxidopyrylium cycloaddition in library-oriented syntheses of troponoids, we became interested in whether it could be used in methoxytropone synthesis. Such an approach would require a reductive ring-opening with removal of the bridging oxygen (**6 → 8**; Scheme 1A). We were inspired by the report of side product **12** described by Wright and co-workers in their development of a furan route to tropolones (Scheme 1B).^12^ The conversion of **10** into **12** was analogous to our desired transformation, and it seemed reasonable that the presence of a methoxy group on cycloadduct **6** instead of the bromide in cycloadduct **10** could be enough to shift the selectivity toward **8**. Thus, a series of oxidopyrylium cycloadducts were generated and subjected to reductive ring-opening conditions (Scheme 2).

**Scheme 2:**
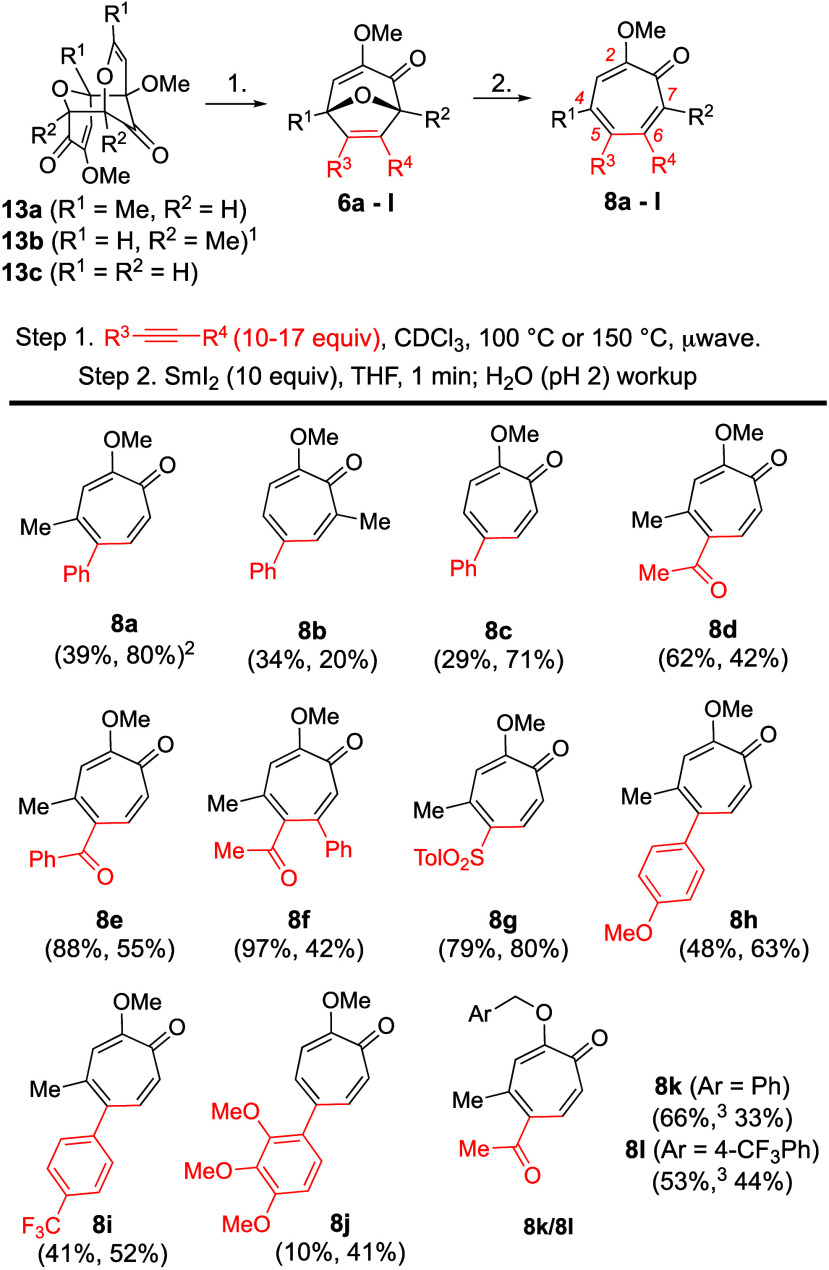
Substrate Scope Studies Notes: ^1^**13b** was synthesized and used as mixture of regioisomeric dimers. ^2^Isolated yields for cycloaddition and reductive ring-opening, listed in order. ^3^The cycloaddition reaction carried out using a modified three-component procedure wherein benzylic alcohols were added to the reaction mixture prior to cycloaddition.

To our delight, the reaction proved to be effective at synthesizing a number of different methoxytropolones. For example, phenylacetylene-derived methoxytropones **8a** and **8c** can be generated in synthetically useful yields, but substitution at the 7-position led to substantially lower yields of the ring-opening (**8b**). Substitution of phenyl groups with either electron-donating (**8h**) or -withdrawing (**8i**) groups was also tolerated and facilitated the synthesis of MTC (**8j**), a widely studied tubulin inhibitor.^13^ In this latter case, the cycloaddition between dimer **13c**(14c) and the associated alkyne was extremely sluggish and low-yielding, even compared to the cycloaddition with phenylacetylene (see **8c** vs **8j**). This is due to a combination of the sluggish activity of dimer **13c** more generally and the combination of steric and electron-donating effects of the aryl acetylene. Sulfonyl (**8g**)- and ketone (**8d**, **8e**)-appended methoxytropones could also be synthesized, as well as the tropone derived from β-phenyl-substituted ynones (**8f**). Finally, the reaction can convert oxabicycles generated through a three-component cycloaddition employing benzylic alcohols (**8k**, **8l**). While benzyl ether **8k** is produced in diminished yield compared to **8d**, the trifluoro-substituted variant **8l** is generated in similar yield.

In prior studies on the conversion of **10** to **11** (Scheme 1), it was hypothesized that hydrolytic conditions would lead to variations of generalized intermediate **16** (Scheme 3A).^12^ Consistent with observations, acidic conditions would help promote the loss of water and lead to competitive production of **12**, whereas a basic workup led to exclusive elimination of the bromide ion to form **11** (generalized as **17**). In the case of the reaction of bicycles **6a**–**l**, an analogous mechanism would lead to a competitive elimination between water and methanol. If this were the case, we would expect a mixture of products. However, the alternative tropolone product was never observed, even under basic workups. Thus, the 3-hydroxy-4-pyrone-derived oxabicycles seem to be biased toward elimination of the bridging oxygen. One possible mechanism that could explain this preference could be *via* elimination of hydroxy(oxo)samarium, which could be promoted by the donor ability of the methoxy group at C2 (Scheme 3B). This mechanism assumes ring-opening through initial opening of the C4–O bond, in analogy to the work of Wright and consistent with ring-opening in our prior acid-catalyzed work. However, a C7–O bond opening can also not be ruled out as a primary or competitive process. Furthermore, in the case of ketone-containing substrates (**8d**–**f**), ring-openings that are initiated by reduction of these appendages are an additional possibility. These alternative sites for reduction could also help explain the lower yields observed for this series.

**Scheme 3:**
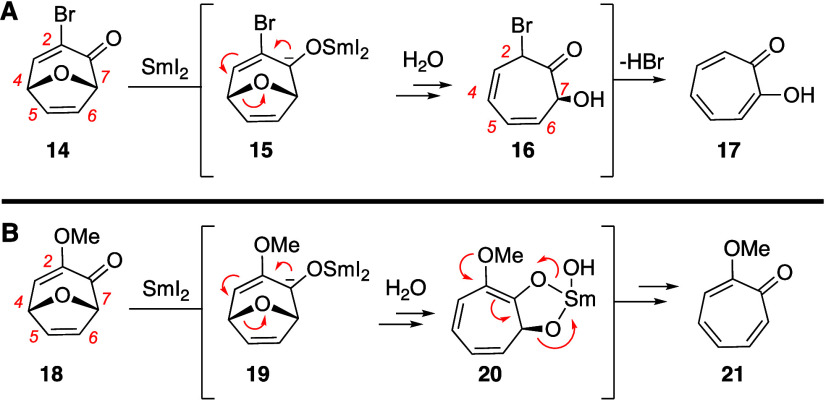
Potential Mechanistic Pathways for Production of Tropolone and Methoxytropolone

Finally, we wanted to highlight the method for the synthesis of tropone-functionalized AC rings. The AC ring of colchicine is directly tied to its tubulin-binding properties.^15^ However, synthetic strategies for its *de novo* construction have been almost universally tied to natural product synthesis.^16^ Outside of cross-coupling strategies from simple 5-halomethoxytropone,^17^ the only exception to this is the synthesis of chloro-AC ring-containing analogs reported by Banwell over 30 years ago (**23**, **24**; Scheme 4A).^18^ The synthesis and biological evaluation of these compounds were an important piece of a broader question on the conformation of the AC ring binding to tubulin. From a synthetic organic chemistry standpoint, the fact that 10 steps are required to convert **22** into **23** highlights the broader challenges to tropone synthesis. We thus felt that **26** and **27** represented close structural homologues of **23** and **24** but could be generated in far fewer steps using this newly established strategy. Dimer **13a** is considered a *de facto* starting material in our lab given its ease of synthesis and scalability.^19^ Meanwhile, alkyne **25** can be made in a single step from commercially available starting materials.^20^ The synthesis of **26** can then be achieved in two steps using the oxidopyrylium cycloaddition/reductive ring-opening sequence. Compound **27** can then be accessed through a demethylation and remethylation sequence followed by its chromatographic separation from isomer **26**.^21^ The antiproliferative activities of **26** and **27** against three different cancer cells matched closely with the activities of **23** and **24**, and the relative activity of these two compounds was consistent with their tubulin-binding ability.^22^ Current efforts are underway to use this chemistry in studies of other novel AC ring analogs.^23^

**Scheme 4:**
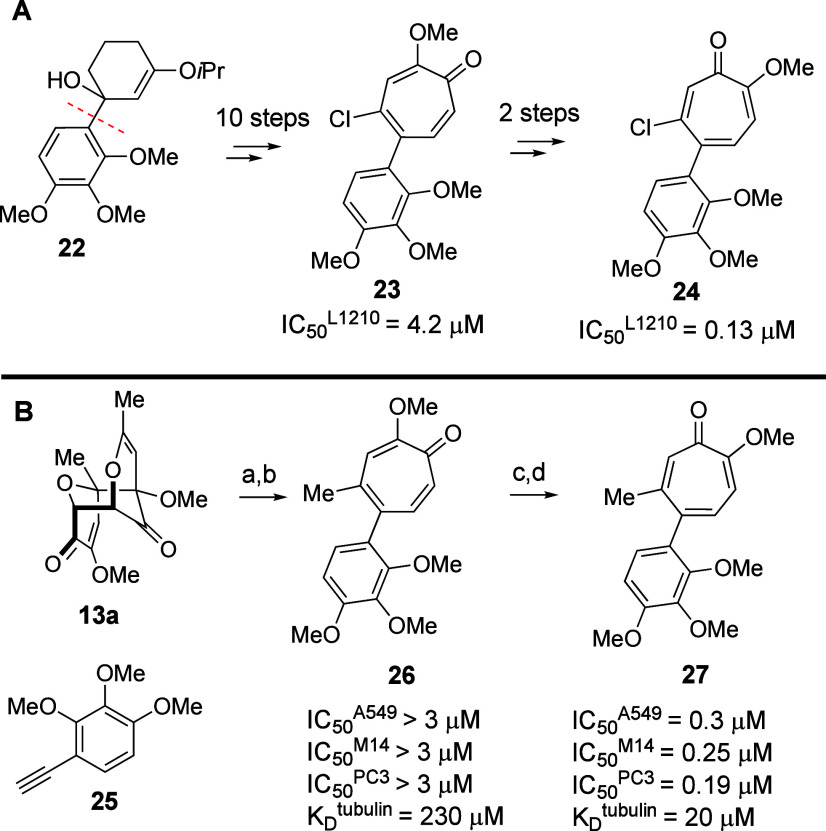
Synthesis of Tropone-Substituted AC Rings: (A) Previously Reported Synthesis by Banwell;^18^ (B) Synthesis of **26** and **27** Conditions: (a) CDCl_3_, 120 °C, 2 h, 50%; (b) SmI_2_ (5 equiv), rt, 2 min, then pH 3 workup, 3 h, 68%; (c) MeOH/HCl (2 N), reflux 16 h, 96%; (d) MeI (5 equiv), K_2_CO_3_ (3 equiv), dicyclohexyl-18-crown-6 (0.1 equiv), CH_3_CN, 82 °C, 24 h, 15%.

The structural versatility provided by 3-hydroxy-4-pyrone-based oxidopyrylium cycloaddition along with selective reduction of the bridging oxygen by samarium iodide provides a new and powerful strategy for tropone and tropolone synthesis. As such, this new strategy could find utility in accessing and studying troponoid-containing compounds.

## Data Availability

The data underlying this study are available in the published article and its Supporting Information.

## Supporting Information Available

Detailed experimental procedures with characterization data along ^1^H and ^13^C NMR spectra of all newly synthesized compounds (PDF)
