# Supplementary material for: Intermolecular Oxidopyrylium (5 + 2) Cycloaddition/Reductive Ring-Opening Strategy for the Synthesis of α-Methoxytropones
Source: J Org Chem. 2024 Nov 12;89(23):17813–7. doi: 10.1021/acs.joc.4c01989 (PMC11629294; doi:10.1021/acs.joc.4c01989)
Supplement: Supplementary file 1 — jo4c01989_si_001.pdf [file jo4c01989_si_001.pdf]

Intermolecular Oxidopyrylium (5+2) Cycloaddition / Reductive Ring-Opening Strategy for the Synthesis of α-Methoxytropones

Orugbani S. Eli,[a,b,‡] Lauren P. Bejcek,[a,b,‡] Anastasiya Lyubimova,[a,b] Dan L.Sackett,[c]

Ryan P. Murelli[a,b,d,*]

[a]*Department of Chemistry, Brooklyn College, The City University of New York, Brooklyn College, Brooklyn, NY, 11210, United States*

[b]*PhD Program in Chemistry, The Graduate Center, The City University of New York, New York, NY, 10016, United States*

[c]*Division of Basic and Translational Biophysics, Eunice Kennedy Shriver National Institute of Child Health and Human Development, National Institute of Health, Bethesda, MD, 20892, United States*

[d]*PhD Program in Biochemistry, The Graduate Center, The City University of New York, New York, NY, 10016, United States*

*Corresponding author, rpmurelli@brooklyn.cuny.edu

Supporting Information

Table of Contents

I. General Information…………………………………………………………………….s-3

II. Synthesis and Characterization of Oxidopyrilium Triflate Salts (4)…………………...s-3

General Procedure……………………………………………………………………………….s-3

5-hydroxy-4-methoxy-2-pyrylium triflate **(4a)**……………………………………………………s-3

3-hydroxy-4-methoxy-2-methylpyrylium triflate **(4b)**…………………………………………….s-3

3-hydroxy-4-methoxypyrylium triflate **(4c)**……………………………………………………..s-4

III. Synthesis and Characterization of Oxidopyrylium Dimers (13)…………………………...s-4

General Procedure……………………………………………………………………………….s-4

(±)-(1R,2S,6S,7R)-6,9-dimethoxy-4,7-dimethyl-3,11-dioxatricyclo[5.3.1.12,6]dodeca-4,8-diene-10,12-dione **(13a)**…………………………………………………………………………………………..s-4

(±)-(1R,2S,6S,7R)-6,9-dimethoxy-1,2-dimethyl-3,11-dioxatricyclo[5.3.1.12,6]dodeca-4,8-diene-10,12-dione **(13b)**…………………………………………………………………………………………..s-4

(±)-(1R,2R,6R,7R)-6,9-dimethoxy-2,7-dimethyl-3,11-dioxatricyclo[5.3.1.12,6]dodeca-4,9-diene-8,12-dione **(13b')**……………………………………………………………………………………………s-5

(1R,2R,6R,7R)-6,9-dimethoxy-3,11-dioxatricyclo[5.3.1.12,6]dodeca-4,9-diene-8,12-dione **(13c)**…………………………………………………………………………………………….s-5

IV. Synthesis and Characterization of Alkyne 25……...……………………...………………...s-6

1-ethynyl-2,3,4-trimethoxybenzene **(25)**…………………...…………………………………s-6

V. Synthesis and Characterization of 8-oxabicyclo[3.2.1]octa-3,6-dienones (6)……………s-6

General Procedure……………………………………………………………………………….s-6

(±)-3-methoxy-6-phenyl-8-oxabicyclo[3.2.1]octa-3,6-dien-2-one **(6c)**……………………………………s-7
(±)-3-methoxy-5-methyl-6-tosyl-8-oxabicyclo[3.2.1]octa-3,6-dien-2-one **(6g)**………………………………...s-7
(±)-(1S,5S)-3-methoxy-6-(4-methoxyphenyl)-5-methyl-8-oxabicyclo[3.2.1]octa-3,6-dien-2-one **(6h)**………..s-7
(±)-(1S,5S)-3-methoxy-6-(2,3,4-trimethoxyphenyl)-8-oxabicyclo[3.2.1]octa-3,6-dien-2-one **(6j)**…………….s-8
(±)-(1S,5S)-6-acetyl-5-methyl-3-((4-(trifluoromethyl)benzyl)oxy)-8-oxabicyclo[3.2.1]octa-3,6-dien-2-one **(6l)**……………………………………………………………………………………………………….s-8
(±)-(1S,5S)-3-methoxy-5-methyl-6-(2,3,4-trimethoxyphenyl)-8-oxabicyclo[3.2.1]octa-3,6-dien-2-one **(S1)**…s-8

VI. Synthesis and Characterization of 2-methoxycyclohepta-2,4,6-trien-1-ones (8)……...s-9

General Procedure………………………………………………………………………………………….s-9
2-methoxy-4-methyl-5-phenylcyclohepta-2,4,6-trien-1-one **(8a)**…………………………………………….s-9
7-methoxy-2-methyl-4-phenylcyclohepta-2,4,6-trien-1-one **(8b)**……………………………………………s-9
2-methoxy-5-phenylcyclohepta-2,4,6-trien-1-one **(8c)**…………………………………………………...s-10
5-acetyl-2-methoxy-4-methylcyclohepta-2,4,6-trien-1-one **(8d)**……………………………………………s-10
5-benzoyl-2-methoxy-4-methylcyclohepta-2,4,6-trien-1-one **(8e)**………………………………………….s-10
5-acetyl-2-methoxy-4-methyl-6-phenylcyclohepta-2,4,6-trien-1-one **(8f)**………………………………….s-10
2-methoxy-4-methyl-5-tosylcyclohepta-2,4,6-trien-1-one **(8g)**……………………………………………..s-11
2-methoxy-5-(4-methoxyphenyl)-4-methylcyclohepta-2,4,6-trien-1-one **(8h)**……………………………..s-11
2-methoxy-4-methyl-5-(4-(trifluoromethyl)phenyl)cyclohepta-2,4,6-trien-1-one **(8i)**………………………..s-11
2-methoxy-5-(2,3,4-trimethoxyphenyl)cyclohepta-2,4,6-trien-1-one **(8j)**…………………………………s-12
5-acetyl-2-(benzyloxy)-4-methylcyclohepta-2,4,6-trien-1-one **(8k)**………………………………………..s-12
5-acetyl-4-methyl-2-((4-(trifluoromethyl)benzyl)oxy)cyclohepta-2,4,6-trien-1-one **(8l)**……………………..s-12
2-methoxy-4-methyl-5-(2,3,4-trimethoxyphenyl)cyclohepta-2,4,6-trien-1-one **(26)**…………………………s-12

VII. Methoxytropone Demethylation Synthesis and Characterization…..…………………s-13

General Procedure………………………………………………………………………………………...s-13
2-hydroxy-4-methyl-5-(2,3,4-trimethoxyphenyl)cyclohepta-2,4,6-trien-1-one **(S2)**…………………………s-13

VIII. Methoxytropone Re-Methylation Synthesis and Characterization…………………..s-14

General Procedure………………………………………………………………………………………...s-14
2-methoxy-6-methyl-5-(2,3,4-trimethoxyphenyl)cyclohepta-2,4,6-trien-1-one **(27)**…………………………s-14

IX. Biological and Biochemical Evaluation of 26 and 27…………...………..………………..s-15

X. NMR Spectra……………………………………………………………………….…s-16

References…………………………………………………………………………………...s-67

I. General Information

All starting materials and reagents were purchased from commercially available sources and used without further purification, except for CH_2Cl_2 and benzene, which were purified on a solvent purification system prior to the reaction. ^{1}H NMR shifts are measured using the solvent residual peak as the internal standard ($CHCl_3$ δ 7.26, D_2O δ 4.79), and reported as follows: chemical shift, multiplicity (s = singlet, bs = broad singlet, d = doublet, t = triplet, dd = doublet of doublet, q = quartet, m = multiplet), coupling constant (Hz), and integration. ^{13}C{1H} NMR shifts are measured using the solvent residual peak as the internal standard ($CDCl_3$ δ 77.20) and reported as chemical shifts. Infrared (IR) spectral bands are characterized as broad (br), strong (s), medium (m), and weak (w). Microwave reactions were performed via the Biotage Initiator 2.5. Purification via reverse phase column chromatography was performed on the Biotage Isolera Prime, with Biotage SNAP 12g cartridges, in a solvent system of acetonitrile in water, each solvent containing 0.05% trifluoroacetic acid (TFA).

II. Synthesis and Characterization of Oxidopyrilium Triflate Salts (4)[1,2]

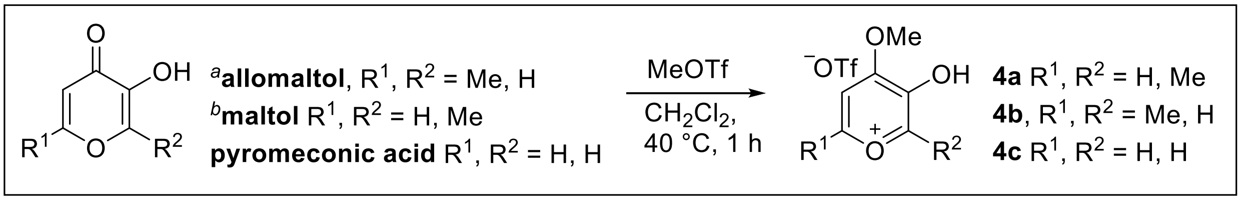

General Procedure: Oxidopyrylium salts were prepared as described in the supporting information.[1,2] To a solution of 3-hydroxy-4-pyrone derivative in CH_2Cl_2, methyl trifluoromethanesulfonate (MeOTf) was added. The mixture was stirred under reflux for 1 h, cooled to room temperature, then evaporated under reduced pressure to yield the crude oil. Crystallization from ethyl acetate (EtOAc) yielded pure solids **4a – c**.

5-hydroxy-4-methoxy-2-pyrylium triflate (4a)[1]

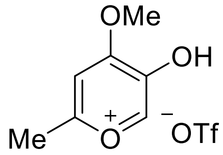

White solid **4a** (9.7 g, 84 %) was obtained from allomaltol[a] (5.0 g, 0.040 mol, 1 eq.). **^{1}H NMR (400 MHz, DMSO-*d6*)** δ 2.75 (s, 3H), 4.27 (s, 3H), 7.93 (s, 1H), 9.00 (s, 1H), 11.68 (br s, 1H); **^{1}H NMR (400 MHz, MeCN-*d3*)** δ 2.73 (s, 3H), 4.28 (s, 3H), 7.55 (s, 1H), 8.76 (s, 1H), 9.11 (s, 1H);

3-hydroxy-4-methoxy-2-methylpyrylium triflate (4b)[3,4]

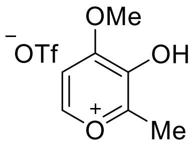

White solid **4b** (6.5 g, 54% yield) obtained from maltol[b] (5.0 g, 0.040 mol, 1 eq.) and methyl trifluoromethanesulfonate (6.5 mL, 0.059 mol, 1.5 eq.) in CH_2Cl_2 (10 mL) for 4 hours. **m.p.**: 99 – 102 °C. **IR (thin film, KBr)**: 3088 (w), 1634 (s), 1554 (w), 1497 (m), 1438 (w), 1258 (b), 1164 (s), 1069 (w), 1033 (s), 962 (w), 903 (w), 827 (w), 750 (b), 636 (s) cm^{-1}. **^{1}H NMR (400 MHz, CD_3CN)** δ 8.80 (d, J = 5.2 Hz, 1H), 7.60 (d, J = 5.2 Hz, 1H), 4.31 (s, 3H), 2.68 (s, 3H).

[a]For preparation of **allomaltol** see: *Org. Synth.* **2019**, *96*, 494-510
[b]**maltol** was commercially sourced from AmBeed

3-hydroxy-4-methoxypyrylium triflate (4c)

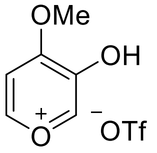

White solid **4c** (1.1 g, 0.040 mol, 89 %) was obtained from pyromeconic acid (0.5 g, 0.004 mol, 1 eq.). Spectra corresponds with previously reported characterization.[5] **^{1}H NMR (400 MHz, DMSO-*d6*):** δ 8.07 (d, *J* = 1.0 Hz, 1H), 8.05 (d, *J* = 1.0 Hz, 1H), 8.04 (d, *J* = 1.0 Hz, 1H), 6.38 (d, *J* = 5.5 Hz, 1H), 3.16 (s, 3H)

III. Synthesis and Characterization of Oxidopyrylium Dimers (13)

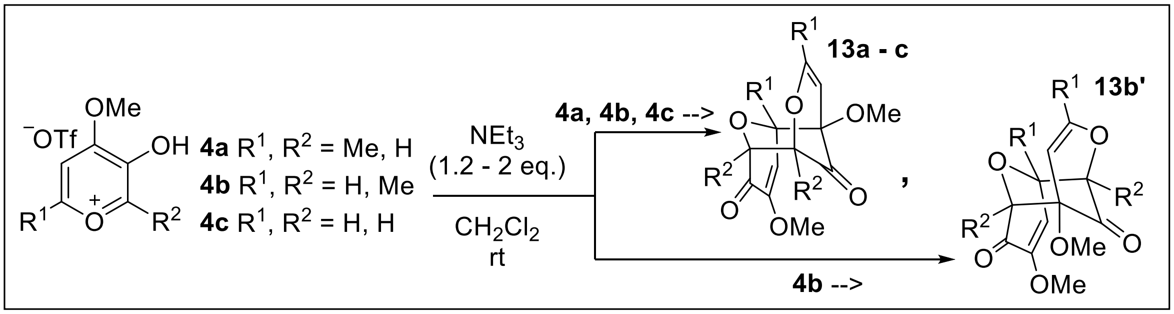

General Procedure: To a solution of triflate salt **4** (1 eq.) in CH_2Cl_2 (0.25 M), triethylamine (1.2 – 2 eq.) was added to the mixture in the flask, dissolving the solid, and left to mix at room temperature for 15 minutes to 2 hours. The reaction was quenched with aqueous ammonium chloride, and the mixture extracted with CH_2Cl_2, (2x). The combined organics were dried over Na_2SO_4, filtered, and concentrated under reduced pressure to yield solid **13**.[6]

(±)-(1*R*,2*S*,6*S*,7*R*)-6,9-dimethoxy-4,7-dimethyl-3,11-dioxatricyclo[5.3.1.1^{2,6}]dodeca-4,8-diene-10,12-dione (13a)

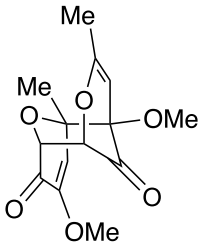

Pale orange solid **13a** (24.4 mg, 73% yield) that decomposes at 161°C was obtained from **4a** (69.8 mg, 0.24 mmol, 1 eq.) and Et_3N (29.1 mg, 0.29 mmol, 1.2 eq.) in $CHCl_3$ (1.2 mL, 0.2 M) at room temperature for 15 minutes. **R$_f$** = 0.29 in 30% ethyl acetate (EtOAc) in hexanes. Spectra corresponds with previously reported characterization data.[2] **^{1}H NMR (400 MHz, CDCl$_3$)** δ 5.94 (s, 1H), 4.79 (d, *J* = 2.7 Hz, 1H), 4.74 (s,1H), 4.47 (d, *J* = 2.7 Hz, 1H), 3.64 (s, 3H), 3.45 (s, 3H), 2.00 (s, 3H), 1.47 (s, 3H).

(±)-(1R,2S,6S,7R)-6,9-dimethoxy-1,2-dimethyl-3,11-dioxatricyclo[5.3.1.1^{2,6}]dodeca-4,8-diene-10,12-dione (13b)*

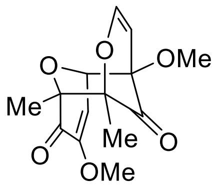

White solid **13b** (22 mg, 0.08 mmol, 45% yield) obtained from a mixture of triflate salt **4b** (100 mg, 0.35 mmol, 1 eq.) and Et_3N (96 µL, 0.35 mmol, 2 eq.) in CH_2Cl_2 (3 mL, 0.1 M) at room temperature for 2 hours. **m.p.:** 129 – 132 °C. **Rf** = 0.14 in 20 % ethyl acetate in hexanes. **IR (ATR, ZnSe)** 3090 (w), 3002 (w), 2941 (br), 2834 (br), 1742 (s), 1699 (s), 1626 (s), 1455

(w), 1358 (m), 1258 (m), 1170 (s), 1139 (s), 1066 (s), 1036 (m), 914 (m), 841 (w), 789 (w). **^{1}H NMR (400 MHz, CDCl$_3$)** δ 6.67 (d, *J* = 5.9 Hz, 1H), 6.01 (d, *J* = 5.1 Hz, 1H), 5.02 (d, *J* = 5.9 Hz, 1H), 4.49 (d, *J* = 5.1 Hz, 1H), 3.63 (s, 3H), 3.49 (s, 3H), 1.55 (s, 3H), 1.25 (s, 3H).

(±)-(1R,2R,6R,7R)-6,9-dimethoxy-2,7-dimethyl-3,11-dioxatricyclo[5.3.1.12,6]dodeca-4,9-diene-8,12-dione (13b')*

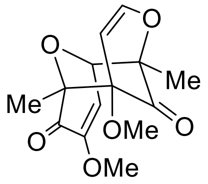

White solid **13b'** (12 mg, 0.04 mmol, 25% yield) obtained from a mixture of triflate salt **4b** (100 mg, 0.35 mmol, 1 eq.) and Et_3N (96 µL, 0.35 mmol, 2 eq.) in CH_2Cl_2 (3 mL, 0.1 M) at room temperature for 2 hours. **m.p. =** 159 – 159 °C. **Rf** = 0.14 in 25 % ethyl acetate. **^{1}H NMR (400 MHz, CDCl$_3$)** δ 6.69 (d, *J* = 6.0 Hz, 1H), 5.64 (d, *J* = 5.2 Hz, 1H), 4.92 (d, *J* = 6.0 Hz, 1H), 4.74 (d, *J* = 5.2 Hz, 1H), 3.60 (s, 3H), 3.43 (s, 3H), 1.45 (s, 3H), 1.39 (s, 3H).

**The stereochemistry of 13b and 13b' was determined by X-ray crystal analysis and has been reported in the reference.*[3]

(±)-(1R,2R,6R,7R)-6,9-dimethoxy-3,11-dioxatricyclo[5.3.1.12,6]dodeca-4,9-diene-8,12-dione (13c)

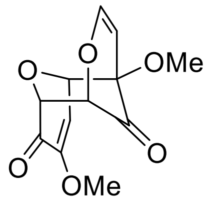

Pale yellow solid **13c** (1.9 g, 0.036 mol, 82% yield) was obtained from **4c** (5.0 g, 0.018 mol, 1 eq.) and Et_3N (5 mL, 36 mmol, 2 eq.) in CH_2Cl_2 (37.5 mL, 0.25 M) at room temperature for 15 minutes. **Rf** = 0.8 in 20% ethyl acetate/hexanes. **m.p.**: 124 -128 °C. **IR (ATR, ZnSe)** 3385 (w), 3072 (w), 2983 (br), 2840 (br), 1748 (s), 1702 (s), 1614 (s), 1453 (w), 1364 (m), 1261 (m), 1206 (m),1158 (s), 1139 (s), 917 (m), 875 (m), 820 (m). **^{1}H NMR (400 MHz, CDCl$_3$)** δ 6.71 (d, *J* = 5.9 Hz, 1H), 6.00 (m, 1H), 4.99 (d, *J* = 5.9 Hz, 1H), 4.89 – 4.80 (m, 1H), 4.61 – 4.54 (m, 1H), 4.48 (m, 1H), 3.62 (s, 3H), 3.46 (s, 3H). **^{13}C{^{1}H} NMR (101 MHz, CDCl$_3$)** δ 197.3, 185.1, 150.8, 149.3, 112.9, 100.5, 86.9, 86.7, 82.9, 79.2, 55.4, 54.2. **HRMS (ESI+ TOF) *m/z*:** $(M+H)^+$ Calc'd for $C_{12}H_{13}O_6^+$: 253.0712. Found: 253.0701. *The stereochemistry of 13c was determined by X-ray crystal analysis and has been reported in the reference.*[7]

IV. Synthesis and Characterization of Alkyne 25

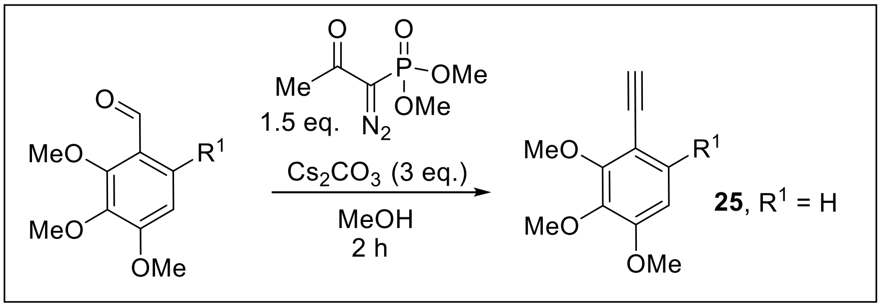

1-ethynyl-2,3,4-trimethoxybenzene (25)

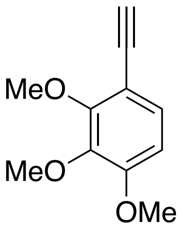

Clear oil **25** (272 mg, 82% yield) was obtained from 2,3,4-trimethoxybenzaldehyde (340 mg, 1.73 mmol, 1 eq.), cesium carbonate (1.7 g, 5.21 mmol, 3 eq.), and dimethyl (1-diazo-2-oxopropyl) phosphonate solution (500 mg, 2.60 mmol, 1.5 eq.) in methanol (4 mL) after 2 hours. **R_f =** 0.65, 20% EtOAc–hexanes. **^{1}H NMR (400 MHz, $CDCl_3$)** δ 7.19 (d, J = 8.4 Hz, 1H), 6.63 (d, J = 8.8 Hz, 1H), 4.00 (s, 3H), 3.89 (s, 3H), 3.89 (s, 3H), 3.22 (s, 1H). Spectra corresponds with previously reported characterization.[8,9]

V. Synthesis and Characterization of 8-oxabicyclo[3.2.1]octa-3,6-dienones (6)

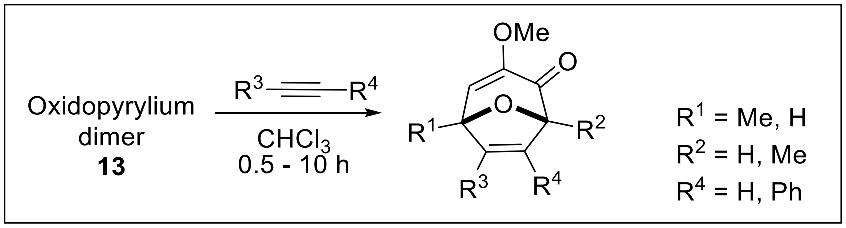

General Procedure: To a solution of oxidopyrylium dimer **13** (1 eq.) in $CDCl_3$ (0.2 M) in a microwave vial was added (10 – 17 eq). The reaction was subjected to microwave irradiation at 120 °C – 150 °C for 30 minutes to 10 hours. The resulting solution was immediately subjected to purification via column chromatography (Biotage Isolera Prime, SiliCycle SiliaSep 10 g silica gel, 40-63 μm 60 Å, solvent gradient: 0-100% EtOAc in hexanes (500 mL).

Cycloadducts **6a**, **6b**, **6d**, **6e**, **6f**, **6i,** and **6k** were synthesized using previously reported procedures.[2,3,10–12] The synthesis and characterization of the other cycloadducts are reported as follows:

(±)-3-methoxy-6-phenyl-8-oxabicyclo[3.2.1]octa-3,6-dien-2-one (6c)

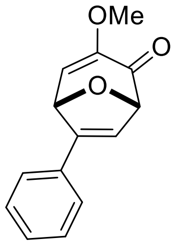

White solid **6c** (106 mg, 29% yield) obtained from oxidopyrylium dimer **13c** (200 mg, 0.79 mmol, 1 eq.) and commercially sourced phenylacetylene (1.2 g, 11.90 mmol, 15 eq.) at 120 °C for 30 minutes. **m.p.** = 150 – 151 °C. **R_f** = 0.76 in 40% ethyl acetate/hexane. **IR (thin film, KBr):** 3587 (w), 3536 (w), 2033 (w), 1704 (m), 1438 (m), 1396 (w), 1235 (w), 1083 (s), 1071 (s), 1060 (s), 1017 (m), 998 (m), 939 (w), 801 (w), 750 (w), 716 (w) 710 (w) 691 (s) 674 (w) cm^{-1}. **^{1}H NMR (400 MHz; $CDCl_3$)** δ 7.43 – 7.35 (m, 5H), 6.52 (d, *J* = 2.5 Hz, 1H), 6.29 (d, *J* = 4.8 Hz, 1H), 5.60 (d, *J* = 4.8 Hz, 1H), 5.12 (d, *J* = 2.5 Hz, 1H), 3.56 (s, 3H). **^{13}C{^{1}H} NMR (101 MHz; $CDCl_3$)** δ 189.6, 156.0, 147.5, 131.7, 129.5, 129.2, 126.1, 119.6, 114.6, 88.2, 79.3, 54.9. **HRMS (ESI+ TOF) *m/z*:** $(M+H)^+$ Calc'd for $C_{14}H_{13}O_3^+$: 229.0865. Found: 229.0862.

(±)-3-methoxy-5-methyl-6-tosyl-8-oxabicyclo[3.2.1]octa-3,6-dien-2-one (6g)

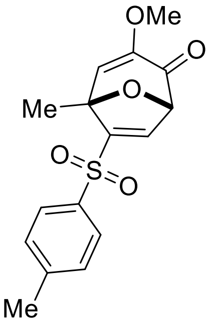

Pale yellow solid **6g** (180 mg, 79% yield) was obtained from oxidopyrylium dimer **13a** (100 mg, 0.36 mmol, 1 eq.) and commercially sourced 1-(ethynylsulfonyl)-4-methylbenzene (965 mg, 5.35 mmol, 15 eq.) in $CDCl_3$ (3.3 mL, 0.1 M) for 2 hours. **m.p.** = 155 – 156 °C. **R_f** = 0.22 in 30 % ethyl acetate/hexane. **IR (thin film, KBr):** 2147 (m), 2138 (w), 2017 (w), 2002 (w), 1969 (m), 1944 (w), 1749 (w), 1435 (w), 1252 (w), 1236 (w), 1180 (w), 1155 (w), 1083 (s), 748 (m), 708 (w), 691 (s) cm^{-1}. **^{1}H NMR (400 MHz; $CDCl_3$)** δ 7.77 (d, *J* = 8.3 Hz, 2H), 7.39 – 7.37 (dd, *J* = 8.0, 0.6 Hz, 2H), 6.89 (dd, *J* = 2.5, 0.3 Hz, 1H), 5.81 (s, 1H), 5.02 (d, *J* = 2.5 Hz, 1H), 3.45 (s, 3H), 2.47 (s, 3H), 1.66 (s, 3H). **^{13}C{^{1}H} NMR (101 MHz; $CDCl_3$)** δ 187.3, 158.8, 145.8, 145.4, 138.1, 135.9, 130.3, 128.6, 119.1, 86.2, 85.7, 54.9, 21.9, 21.5. **HRMS (ESI+ TOF) *m/z*:** $(M+H)^+$ Calc'd for $C_{16}H_{17}O_5S^+$: 321.0791. Found: 321.0796.

(±)-(1*S*,5*S*)-3-methoxy-6-(4-methoxyphenyl)-5-methyl-8-oxabicyclo[3.2.1]octa-3,6-dien-2-one (6h)

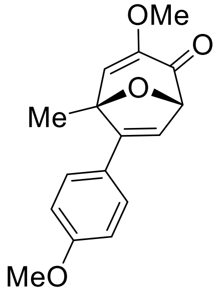

Yellow oil **6h** (137 mg, 48% yield) was obtained from **13a** (150 mg, 0.53 mmol, 1 eq.) in $CDCl_3$ (1 mL, 0.5 M) and 4-ethynylanisole (354 μL, 2.71 mmol, 5 eq.) at 120 °C for 2 hours. **Rf** = 0.12 in 60 % ethyl acetate/pentane. **IR (thin film, KBr):** 2936 (w), 2838 (w), 1844 (m), 1710 (s), 1607 (s), 1510 (s), 1459 (w), 1380 (w), 1254 (s), 1177 (w), 1132 (m), 10 59 (w), 1032 (w), 989 (w), 863 (w), 830 (w), 797 (w) cm^{-1}. **^{1}H NMR (400 MHz, $CDCl_3$)** δ 7.23 (d, *J* = 8.9 Hz, 2H), 6.91 (d, *J* = 8.9 Hz, 2H), 6.16-6.17 (m 2H), 4.96 (m, 1H), 3.83 (s, 3H), 3.59 (s, 3H), 1.68 (s, 3H). **^{13}C{^{1}H} NMR (101 MHz, $CDCl_3$)** δ 190.1, 160.1, 158.4, 146.2, 127.5, 125.6, 121.0, 119.2, 114.3, 86.4, 85.8, 55.5, 54.8, 22.4. **HRMS (ESI+ TOF) *m/z***: $(M+H)^+$ Calc'd for $C_{16}H_{17}O_4^+$: 273.1121. Found: 273.1122.

(±)-(1*S*,5*S*)-3-methoxy-6-(2,3,4-trimethoxyphenyl)-8-oxabicyclo[3.2.1]octa-3,6-dien-2-one (6j)

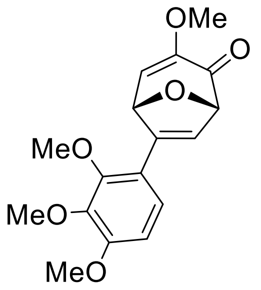

Pale yellow oil **6j** (8.6 mg, 10% yield) was obtained from **13c** (35 mg, 0.14 mmol, 1 eq.) in $CDCl_3$ (500 µL, 0.2 M) and alkyne **25** (350 mg, 1.82 mmol, 13 eq.) at 150 °C for 10 hours. **Rf** = 0.8 in 60% ethyl acetate/pentane. **IR (thin film, KBr):** 2938 (w), 1708 (s), 1611 (w), 1493 (m), 1464 (w), 1415 (w), 1343 (w), 1291 (m), 1241 (w), 1135 (w), 1084 (s), 1005 (w), 914 (s), 815 (w), 742 (m) cm^{-1}. **^{1}H NMR (400 MHz, $CDCl_3$)** δ 6.94 (d, *J* = 8.8 Hz, 1H), 6.68 (d, *J* = 8.8 Hz, 1H), 6.63 (d, *J* = 2.6 Hz, 1H), 6.28 (d, *J* = 4.8 Hz, 1H), 5.60 (d, *J* = 4.8 Hz, 1H), 5.09 (d, *J* = 2.6 Hz, 1H), 3.90 (s, 3H), 3.89 (s, 3H), 3.88 (s, 3H), 3.56 (s, 3H). **^{13}C{^{1}H} NMR (101 MHz, $CDCl_3$)** δ 190.0, 154.8, 152.4, 152.2, 147.1, 142.9, 122.7, 120.5, 119.2, 115.7, 107.5, 87.9, 80.2, 61.0, 60.7, 56.2, 54.8. **HRMS (ESI+ TOF) *m/z*:** $(M+H)^+$ Calc'd for $C_{17}H_{19}O_6^+$: 319.1176. Found: 319.1180.

(±)-(1*S*,5*S*)-6-acetyl-5-methyl-3-((4-(trifluoromethyl)benzyl)oxy)-8-oxabicyclo[3.2.1]octa-3,6-dien-2-one (6l)

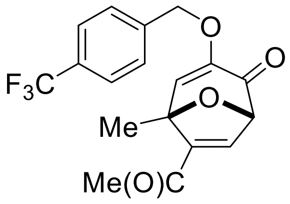

White solid **6l** (66.4 mg, 53%) was obtained from dimer **13a** (100 mg, 0.35 mmol, 1 eq.) and 4-(trifluoromethyl)benzyl alcohol (469 µL, 9.6 eq.) in CH_2Cl_2 (1.37 mL, 0.26 M) stirred at 60 °C in an oil bath for 5 hours, followed by addition of 3-butyn-2-one (242.9 mg, 3.6 mmol, 10 eq.) and the mixture further stirred at 120 °C under microwave for 30 minutes. **m.p.** = 116 – 119 °C. **Rf** = 0.23 in 20% ethyl acetate/hexane. **IR (thin film, KBr):** 1708 (m), 1669 (m), 1606 (m), 1419 (w), 1364 (w), 1328 (s), 1274 (m), 1221 (m), 1167 (s), 1110 (s), 1065 (s), 1020 (m), 984 (m), 958 (m), 922 (w), 877 (s), 847 (m), 830 (m), 795 (m), 784 (w), 772 (m), 702 (m), 678 (m) cm^{-1}. **^{1}H NMR (400 MHz; $CDCl_3$)** *δ* 7.61 (d, *J* = 8.0 Hz, 2H), 7.45 (dd, *J* = 8.6, 0.6 Hz, 2H), 7.05 (dd, *J* = 2.6, 0.4 Hz, 1H), 6.20 (s, 1H), 5.08 (d, *J* = 2.6 Hz, 1H), 4.77 (q, *J* = 10.2 Hz, 2H), 2.37 (s, 3H), 1.71 (s, 3H). **^{13}C{^{1}H} NMR (101 MHz; $CDCl_3$)** *δ* 194.4, 188.5, 156.5, 143.9, 139.5, 138.9, 127.8, 125.8, 125.8, 125.7, 125.68, 121.9, 86.2, 86.1, 68.8, 27.9, 21.4. **HRMS (ESI+ TOF) *m/z*:** $(M+H)^+$ Calc'd for $C_{18}H_{16}F_3O_4^+$: 353.1002. Found: 353.0997.

(±)-(1*S*,5*S*)-3-methoxy-5-methyl-6-(2,3,4-trimethoxyphenyl)-8-oxabicyclo[3.2.1]octa-3,6-dien-2-one (S1)

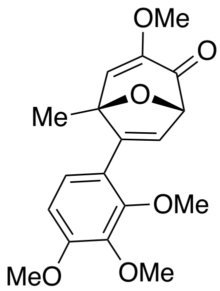

Yellow oil **S1** (15 mg, 50% yield) obtained from oxidopyrylium dimer **13a** (13 mg, 0.047 mmol, 1 eq.) and alkyne **25** (136 g, 0.71 mmol, 15 eq.) at 120 °C for 2 hours. **Rf** = 0.68 in 60 % ethyl acetate/hexane. **IR (thin film, KBr):** 3092 (w), 2968 (m), 2937 (m), 2841 (w), 1699 (s), 1597 (s), 1492 (s), 1461 (s), 1408 (m), 1345 (w), 1280 (m), 1093 (s), 1045 (m), 982 (s), 853 (m), 804 (m) cm^{-1}. **^{1}H NMR (400 MHz; $CDCl_3$)** δ 6.84 (d, *J* = 8.6 Hz, 1H), 6.68 (s, 1H), 6.30 (d, *J* = 2.7 Hz, 1H), 6.26 (s, 1H), 4.93 (d, *J* = 2.7 Hz, 1H), 3.89 (s, 3H), 3.87 (s, 3H), 3.76 (s, 3H), 3.63 (s, 3H), 1.56 (s, 3H). **^{13}C{^{1}H} NMR (101 MHz, $CDCl_3$)** δ 190.8, 156.7, 154.4, 151.1, 145.1, 142.6, 124.5, 123.8, 121.9, 120.7, 107.7, 87.2, 86.2, 61.2, 61.1, 56.2, 54.8, 21.8. **HRMS (ESI+ TOF) *m/z*:** $(M+H)^+$ Calc'd for $C_{18}H_{20}O_6^+$: 333.1338. Found: 333.1336.

VI. Synthesis and Characterization of 2-methoxycyclohepta-2,4,6-trien-1-ones (8)

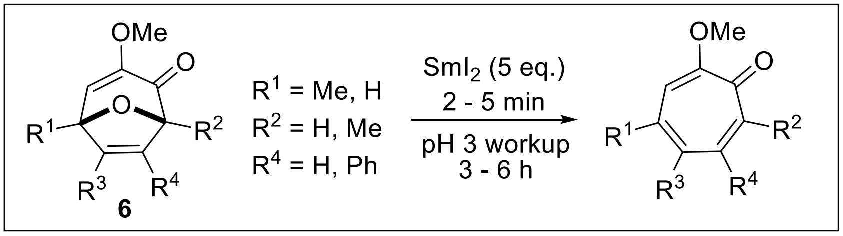

General Procedure: To a flame-dried microwave vial equipped with a stir bar, was added the 8-oxabicyclo[3.2.1]octa-3,6-dienone cycloadduct (1 eq) in THF (0.2 M). The reaction vessel was purged for 2 – the 5 minutes with argon, and a 0.1 M solution of samarium iodide in THF was added via syringe (5 eq.). The resulting solution was allowed to stir at room temperature for 2 minutes before being quenched with an equivalent volume of pH 3 phosphate buffer. The cloudy mixture was then stirred at room temperature for 3 – 6 hours after which the THF was removed *en vacuo*. The mixture was then diluted with deionized water, extracted with Et_2O (3x), water (1x), and brine (1x), then dried with Na_2SO_4, filtered and concentrated, and then purified using chromatography. (Biotage Isolera Prime, SiliCycle, SiliaSep 40 g silica gel, 40 – 63 µm 60 Å, solvent gradient: 0-100% acetonitrile in dichloromethane (500 mL).

2-methoxy-4-methyl-5-phenylcyclohepta-2,4,6-trien-1-one (8a)

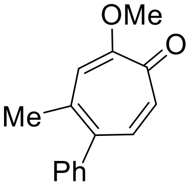

Pale-yellow oil **8a** (7.5 mg, 80%) was obtained from **6a** (10 mg, 0.04 mmol). **Rf** = 0.10 in 60% ethyl acetate/pentane. **IR (thin film, KBr):** 2882 (w), 2843 (w), 2363 (w), 1621 (m), 1571 (s), 1473 (w), 1456 (w), 1440 (w), 1303 (m), 1268 (m), 1190 (w), 1159 (s), 974 (w), 879 (w), 852 (w), 777 (w), 709 (w) cm^{-1}. **^{1}H NMR (400 MHz, CDCl$_3$)** δ 7.46 – 7.40 (m, 2H), 7.39 – 7.33 (m, *J* = 5.1, 3.7 Hz, 1H), 7.24 – 7.19 (m, 3H), 7.11 – 7.06 (m, *J* = 12.8 Hz, 1H), 6.77 (s, 1H), 3.97 (s, 3H), 2.25 (s, 3H). **^{13}C{^{1}H} NMR (101 MHz, CDCl$_3$)** δ 179.3, 162.8, 143.4, 140.9, 140.7, 133.7, 128.8, 128.6, 127.6, 118.0, 56.3, 26.9. **HRMS (ESI+ TOF) *m/z*:** $(M+H)^+$ Calc'd for $C_{15}H_{15}O_2^+$: 227.1067. Found: 227.1063.

7-methoxy-2-methyl-4-phenylcyclohepta-2,4,6-trien-1-one (8b)

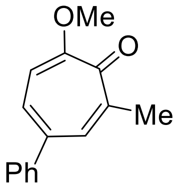

Clear oil **8b** (4.5 mg, 20%) was obtained from **6b** (25 mg, 0.10 mmol) as a clear oil. **Rf** = 0.18 in 60% ethyl acetate/pentane. **IR (thin film, KBr):** 3357 (br), 2917 (br), 1717 (w), 1531 (m), 1558 (m), 1491 (w), 1461 (w), 1366 (m), 1285 (w), 1247 (s), 1181 (m), 1081 (m), 1049 (m), 981 (w), 700 (m) cm^{-1}. **^{1}H NMR (400 MHz, CDCl$_3$)** δ 7.75 (s, 1H), 7.53 – 7.35 (m, 6H), 7.19 (dd, *J* = 10.2, 1.5 Hz, 1H), 6.85 (d, *J* = 10.2 Hz, 1H), 3.97 (s, 3H), 2.46 (s, 3H). **^{13}C{^{1}H} NMR (101 MHz, CDCl$_3$)** δ 179.4, 162.3, 146.5, 143.3, 140.9, 137.7, 129.0, 128.0, 127.7, 112.7, 56.4, 24.5. **HRMS (ESI+ TOF) *m/z*:** $(M+H)^+$ Calc'd for $C_{15}H_{15}O_2^+$: 227.1067. Found: 227.1070.

2-methoxy-5-phenylcyclohepta-2,4,6-trien-1-one (8c)

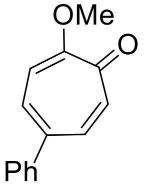

Yellow solid **8c** (15 mg, 71% yield) was obtained from cycloadduct **6c** (46 mg, 0.20 mmol, 1 eq.) and samarium iodide (407 mg, 1.007 mmol, 5 eq.). **m.p.** = 141 – 143 °C. **Rf** = 0.11 in 50 % ethyl acetate/hexane. **IR (thin film, KBr):** 2933 (w), 2195 (w), 2161 (w), 1709 (m), 1698 (m), 1602 (w), 1568 (w), 1541 (w), 1437 (m), 1397 (m), 1234 (m), 1074 (s), 1000 (m), 932 (w), 846 (w), 807 (w), 756 (m), 745 (m), 715 (w), 695 (m), 684 (m), 678 (m) cm^{-1}. **^{1}H NMR (400 MHz; $CDCl_3$)** δ 7.54 (dd, *J* = 12.6, 2.0 Hz, 1H), 7.49 – 7.36 (m, 5H), 7.32 (dd, *J* = 12.6, 0.4 Hz, 1H), 7.28 – 7.25 (ddd, *J* = 10.4, 2.1, 0.4 Hz, 1H), 6.85 (dd, *J* = 10.4, 0.5 Hz, 1H), 3.99 (d, *J* = 0.5 Hz, 3H). **^{1}H NMR (400 MHz; CD_3CN)** δ 7.58 – 7.53 (m, 3H), 7.49 – 7.44 (m, 2H), 7.42 – 7.38 (m, 1H), 7.33 (ddd, *J* = 10.4, 2.1, 0.5 Hz, 1H), 7.15 (dd, *J* = 12.6, 0.5 Hz, 1H), 6.97 (dd, *J* = 10.4, 0.5 Hz, 1H), 3.91 (d, *J* = 0.6 Hz, 3H). **^{13}C{^{1}H} NMR (101 MHz; $CDCl_3$)** δ 180.0, 164.4, 142.3, 141.8, 138.1, 137.2, 131.4, 129.1, 128.3, 127.5, 113.0, 56.5. **HRMS (ESI+ TOF) *m/z*:** $(M+H)^+$ Calc'd for $C_{14}H_{13}O_2^+$: 213.0916. Found: 213.0913.

5-acetyl-2-methoxy-4-methylcyclohepta-2,4,6-trien-1-one (8d)

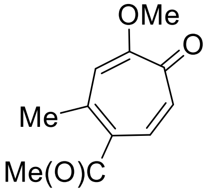

Clear oil **8d** (4.8 mg, 42%) was obtained from **6d** (12.1 mg, 0.06 mmol). **Rf** = 0.10 in 60 % ethyl acetate/ pentane. **IR (thin film, KBr):** 2929 (br), 1696 (m), 1619 (s), 1578 (s), 1481 (m), 1357 (m), 1311 (w), 1270 (m), 1229 (s), 1161 (s), 976 (w), 873 (w), 850 (w), 516 (w) cm^{-1}. **^{1}H NMR (400 MHz, $CDCl_3$)** δ 7.17 (d, *J* = 12.7 Hz, 1H), 7.10 (d, *J* = 12.7 Hz, 1H), 6.62 (s, 1H), 3.97 (s, 3H), 2.51 (s, 3H), 2.43 (s, 3H). **^{13}C{^{1}H} NMR (101 MHz, $CDCl_3$)** δ 203.9, 179.3, 164.0, 142.0, 139.2, 134.3, 134.2, 117.6, 56.5, 30.7, 25.7. **HRMS (ESI+ TOF) *m/z*:** $(M+H)^+$ Calc'd for $C_{11}H_{13}O_3^+$: 193.0859. Found: 193.0862.

5-benzoyl-2-methoxy-4-methylcyclohepta-2,4,6-trien-1-one (8e)

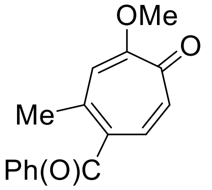

Brown oil **8e** (7.3 mg, 55% yield) was obtained from **6e** (14 mg, 0.05 mmol). **R_f** = 0.36 in 80% ethyl acetate/hexane. **IR (thin film, KBr):** 3447 (br), 1668 (s), 1621 (s), 1594 (s), 1578 (s), 1483 (m), 1449 (m), 1310 (m), 1248 (s), 1161 (s), 979 (w), 914 (w), 873 (w), 850 (w), 714 (m), 689 (w) cm^{-1}. **^{1}H NMR (400 MHz; $CDCl_3$)** *δ* 7.84 – 7.81 (m, 2H), 7.66 – 7.61 (m, 1H), 7.52 – 7.43 (m, 2H), 7.11 (q, *J* = 10.0 Hz, 2H), 6.70 (s, 1H), 4.00 (s, 3H), 2.31 (s, 3H). **^{13}C{^{1}H} NMR (101 MHz; $CDCl_3$)** *δ* 197.8, 179.5, 164.1, 142.1, 137.6, 136.2, 135.2, 134.4, 134.1, 130.1, 129.2, 117.3, 56.6, 25.6. **HRMS (ESI+ TOF) *m/z*:** $(M+H)^+$ Calc'd for $C_{16}H_{15}O_3^+$: 255.1021. Found: 255.1019.

5-acetyl-2-methoxy-4-methyl-6-phenylcyclohepta-2,4,6-trien-1-one (8f)

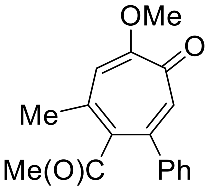

Beige solid **8f** (14.3 mg, 42% yield) was obtained from **6f** (37 mg, 0.13 mmol). **m.p.** = 123 – 126 °C. **R_f** = 0.31 in 80% ethyl acetate/hexane. **IR (thin film, KBr):** 3391 (br), 2934 (w), 2847 (w), 1776 (w), 1705 (m), 1662 (m), 1637 (m), 1609 (s), 1588 (s), 1447 (m), 1381 (m), 1354 (m), 1300 (m), 1284 (m), 1227 (m), 1196 (s), 1173 (s), 1052 (w), 981 (w), 899 (w), 774 (w),

732 (w), 705 (m), 599 (w), 518 (w), 460 (w) cm^{-1}. **^{1}H NMR (400 MHz; CDCl$_3$)** *δ* 7.38 – 7.27 (m, 5H), 7.16 (d, *J* = 0.4 Hz, 1H), 6.66 (s, 1H), 3.97 (d, *J* = 0.6 Hz, 3H), 2.34 (d, *J* = 0.5 Hz, 3H), 1.83 (s, 3H). **^{13}C{^{1}H} NMR (101 MHz; CDCl$_3$)** *δ* 205.5, 178.6, 163.2, 147.5, 142.0, 141.3, 138.8, 137.4, 129.5, 128.9, 128.6, 117.3, 56.5, 32.0, 25.9. **HRMS (ESI+ TOF) *m/z***: (M+H)$^+$ Calc'd for $C_{17}H_{17}O_3^+$: 269.1179. Found: 269.1176.

2-methoxy-4-methyl-5-tosylcyclohepta-2,4,6-trien-1-one (8g)

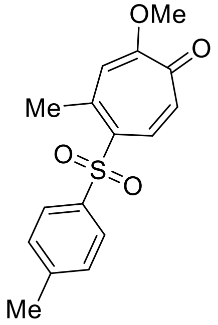

White solid **8g** (38 mg, 80% yield) was obtained from cycloadduct **6g** (50 mg, 0.16 mmol, 1 eq.) and samarium iodide (315 mg, 0.780 mmol, 5 eq.). **m.p** = 166 – 168 °C. **Rf** = 0.14 in 60 % ethyl acetate/hexane. **IR (thin film, KBr):** 2961 (w), 2932 (w), 1715 (w), 1642 (w), 1437 (w), 1362 (w), 1063 (s), 993 (m), 950 (m), 807 (w), 749 (m), 695 (s), cm^{-1}. **^{1}H NMR (400 MHz; CDCl$_3$)** δ 8.25 (d, *J* = 13.3 Hz, 1H), 7.7 – 7.70 (m, 2H), 7.33 (dd, *J* = 8.5, 0.6 Hz, 2H), 7.15 (d, *J* = 13.3 Hz, 1H), 6.53 (s, 1H), 3.96 (d, *J* = 0.4 Hz, 3H), 2.60 (s, 3H), 2.43 (s, 3H). **^{13}C{^{1}H} NMR (101 MHz; CDCl$_3$)** δ 178.5, 165.5, 148.2, 144.8, 138.3, 137.8, 134.3, 133.0, 130.1, 127.6, 117.7, 56.8, 26.7, 21.8. **HRMS (ESI+ TOF) *m/z*:** (M+H)$^+$ Calc'd for $C_{16}H_{17}O_4S^+$: 305.0848. Found: 305.0846.

2-methoxy-5-(4-methoxyphenyl)-4-methylcyclohepta-2,4,6-trien-1-one (8h)

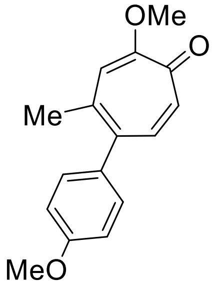

Yellow oil **8h** (12 mg, 63%) was obtained from **6h** (20 mg, 0.07 mmol). **Rf** = 0.12 in 60% ethyl acetate/pentane. **IR (thin film, KBr):** 2935 (br), 2837(w), 2359 (w), 1607 (s), 1575 (s), 1512 (s), 1457 (m), 1288 (m), 1252 (s), 1178 (m), 1158 (m), 1033 (m), 976 (w), 834 (m) cm^{-1}. **^{1}H NMR (400 MHz, CDCl$_3$)** δ 7.24 (d, *J* = 12.8 Hz, 1H), 7.17 (d, 2H), 7.10 (d, *J* = 12.8 Hz, 1H), 6.97 (d, *J* = 8.8 Hz, 2H), 6.79 (s, 1H), 3.99 (s, 3H), 3.88 (s, 3H), 2.29 (s, 3H). **^{13}C{^{1}H} NMR (101 MHz, CDCl$_3$)** δ 179.3, 162.6, 159.0, 141.1, 140.8, 140.7, 135.8, 133.6, 129.9, 118.1, 114.1, 56.2, 55.5, 27.0. **HRMS (ESI+ TOF) *m/z*:** (M+H)$^+$ Calc'd for $C_{16}H_{17}O_3^+$: 257.1172. Found: 257.1178.

2-methoxy-4-methyl-5-(4-(trifluoromethyl)phenyl)cyclohepta-2,4,6-trien-1-one (8i)

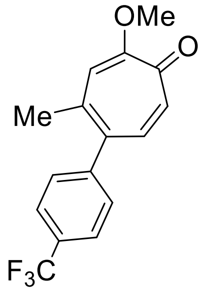

Clear oil **8i** (22 mg, 52%) was obtained from **6i** (45 mg, 0.15 mmol) as a. **Rf** = 0.11 in 60% ethyl acetate/ pentane. **IR (thin film, KBr):** 2930 (w), 2838 (w), 1629 (m), 1571 (s), 1464 (w), 1325 (s), 1271 (m), 1160 (m), 1107 (m), 1067 (m), 1017 (w), 977 (w), 879 (w) cm^{-1}. **^{1}H NMR (400 MHz, CDCl$_3$)** δ 7.70 (d, *J* = 8.0 Hz, 2H), 7.36 (d, *J* = 7.9 Hz, 2H), 7.14 (d, *J* = 12.7 Hz, 1H), 7.08 (d, *J* = 12.8 Hz, 1H), 6.75 (s, 1H), 3.98 (s, 3H), 2.23 (s, 3H). **^{13}C{^{1}H} NMR (101 MHz, CDCl$_3$)** δ 179.2, 163.1, 146.9, 140.9, 139.8, 139.1, 133.9, 130.0 (q, *J* = 32.2 Hz), 129.2, 125.8 (q, 3.7 Hz), 124.2 (q, *J* = 345.5 Hz), 117.6, 56.34, 26.9. **HRMS (ESI+ TOF) *m/z*:** (M+H)$^+$ Calc'd for $C_{16}H_{14}F_3O_2^+$: 295.0940. Found: 295.0941.

2-methoxy-5-(2,3,4-trimethoxyphenyl)cyclohepta-2,4,6-trien-1-one (8j)

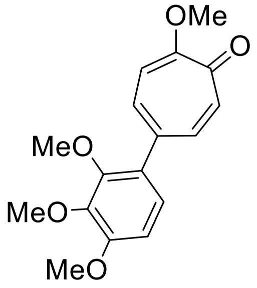

Clear oil **8j** (6.1 mg, 41%) was obtained from **6j** (15 mg, 0.05 mmol). **^{1}H NMR (400 MHz, CDCl$_3$)** δ 7.45 (m, 1H), 7.26 (m, 1H), 7.16 (m, 1H), 6.96 (m, 1H), 6.80 (m, 1H), 6.73 (m, 1H), 4.00 (s, 3H), 3.92 (s, 3H), 3.90 (s, 3H), 3.72 (s, 3H). Spectra is consistent with previously reported characterization.[13]

5-acetyl-2-(benzyloxy)-4-methylcyclohepta-2,4,6-trien-1-one (8k)

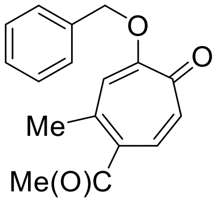

Yellow oil **8k** (9.2 mg, 33% yield) was obtained from **6k** (29 mg, 0.10 mmol). **Rf** = 0.35 in 50% ethyl acetate/hexane. **IR (thin film, KBr):** 2922 (w), 1696 (s), 1618 (s), 1580 (s), 1482 (m), 1454 (m), 1381 (w), 1356 (m), 1309 (m), 1268 (m), 1222 (s), 1154 (s), 1029 (w), 964 (w), 914 (w), 851 (w), 739 (m), 699 (m), 514 (w) cm^{-1}. **^{1}H NMR (400 MHz; CDCl$_3$)** *δ* 7.44 – 7.34 (m, 5H), 7.19 (d, *J* = 2.0 Hz, 2H), 6.79 (s, 1H), 5.27 (s, 2H), 2.49 (s, 3H), 2.35 (s, 3H). **^{13}C{^{1}H} NMR (101 MHz; CDCl$_3$)** *δ* 203.9, 179.5, 163.0, 141.9, 139.5, 135.1, 134.7, 134.0, 129.0, 128.7, 127.5, 120.4, 71.3, 30.7, 25.6. **HRMS (ESI+ TOF) *m/z***: (M+H)$^+$ Calc'd for $C_{17}H_{17}O_3^+$: 269.1178. Found: 269.1173.

5-acetyl-4-methyl-2-((4-(trifluoromethyl)benzyl)oxy)cyclohepta-2,4,6-trien-1-one (8l)

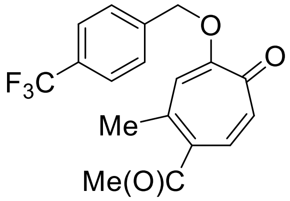

Beige solid **8l** (12.6 mg, 44% yield) was obtained from **6l** (30 mg, 0.09 mmol). **m.p.** = 105 – 110 °C. **Rf** = 0.38 in 50% ethyl acetate/hexane. **IR (thin film, KBr):** 2927 (w), 1697 (m), 1619 (s), 1582 (s), 1483 (m), 1421 (m), 1382 (w), 1357 (m), 1326 (s), 1269 (m), 1223 (s), 1162 (s), 1123 (s), 1055 (s), 1019 (m), 871 (m), 849 (m), 826 (m), 663 (w), 595 (w), 514 (w) cm^{-1}. **^{1}H NMR (400 MHz; CDCl$_3$)** *δ* 7.66 (d, *J* = 8.1 Hz, 2H), 7.57 (d, *J* = 8.1 Hz, 2H), 7.19 (d, *J* = 1.8 Hz, 2H), 6.75 (s, 1H), 5.29 (s, 2H), 2.50 (s, 3H), 2.37 (s, 3H). **^{13}C{^{1}H} NMR (101 MHz; CDCl$_3$)** *δ* 203.8, 179.4, 162.6, 141.9, 140.2, 139.1, 134.9, 134.4, 127.7, 126.1, 126.0, 126.0, 120.8, 70.4, 30.7, 25.6. **HRMS (ESI+ TOF) *m/z***: (M+H)$^+$ Calc'd for $C_{18}H_{16}F_3O_3^+$: 337.1053. Found: 337.1048.

2-methoxy-4-methyl-5-(2,3,4-trimethoxyphenyl)cyclohepta-2,4,6-trien-1-one (26)

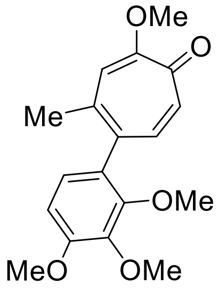

Off-white solid **26** (61 mg, 68% yield) was obtained from cycloadduct **S1** (70 mg, 0.21 mmol, 1 eq.) and samarium diiodide (10.5 mL, 5 eq.). **m.p.** = 145-146 °C. **R$_f$** = 0.24 in 60% ethyl acetate/hexanes. **IR (ATR, ZnSe):** 2937 (w), 1655 (w), 1623 (w), 1568 (s), 1462 (m), 1410 (m), 1335 (w), 1310 (m), 1293 (m), 1258 (s), 1204 (m), 1154 (s), 1107 (s), 1092 (s), 1066 (m), 1018 (m), 1003 (s), 978 (w), 881 (w), 864 (w), 844 (m), 816 (w), 790 (w) cm^{-1}. **^{1}H NMR (400 MHz; CDCl$_3$)** δ 7.16 (d, *J* = 12.7 Hz, 1H), 7.06 (dd, *J* = 12.7, 0.5 Hz, 1H), 6.78 (d, *J* = 8.6 Hz, 1H) 6.77 (s, 1H), 6.71 (d, *J* = 8.6 Hz, 1H), 3.96 (d, *J* = 0.6 Hz, 3H), 3.90 (d, *J* = 3.2 Hz, 6H), 3.67 (s, 3H), 2.21 (s, 3H). **^{13}C{^{1}H} NMR (101 MHz, CDCl$_3$)** δ 179.4, 162.8, 153.7, 150.9, 142.6, 142.1, 141.2, 137.1, 133.4, 130.0,

124.2, 117.8, 107.6, 100.2, 61.2, 56.3, 56.2, 26.7. **HRMS (ESI+ TOF) *m/z*:** $(M+H)^+$ Calc'd for $C_{18}H_{20}O_5^+$: 317.1389. Found: 317.1387.

VII. Methoxytropone Demethylation Synthesis and Characterization

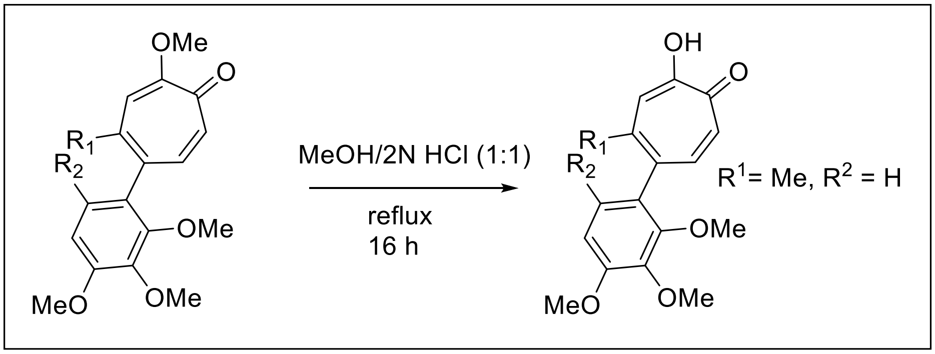

General Procedure:[14] In a microwave vial, the 2-methoxy(2,3,4-trimethoxyphenyl)cyclohepta-2,4,6-trienone (1 eq.) was dissolved in a 1:1 mixture of MeOH:HCl (2N) (0.02M) and the mixture let to stir under reflux for 16 hours. The reaction was left to cool to room temperature and diluted with methylene chloride and water. The aqueous layer neutralized with 5% sodium bicarbonate (aq.) and the layers were separated. The aqueous layer was then extracted three times with methylene chloride, and the combined extract dried with anhydrous sodium sulfate, filtered and evaporated to yield the dark oil.

2-hydroxy-4-methyl-5-(2,3,4-trimethoxyphenyl)cyclohepta-2,4,6-trien-1-one (S2)

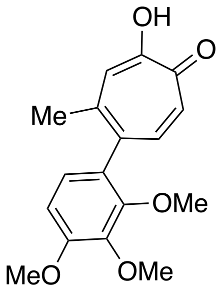

Dark brown oil **S2** (16.5 mg, 96% yield) was obtained from methoxytropone **26** (18 mg, 0.057 mmol, 1 eq.) in 1:1 mixture of MeOH: 2N HCl (3.2 mL). $\mathbf{R_f}$ = 0.35 in 80 % ethyl acetate: hexane. **IR (ATR, ZnSe):** 3203 (br), 2939 (w), 1598 (m), 1546 (m), 1493 (m), 1456 (m), 1441 (s), 1408 (s), 1330 (w), 1290 (m), 1259 (s), 1229 (m), 1165 (m), 1106 (m), 1088 (s), 997 (s), 815 (m) cm^{-1}. **^{1}H-NMR (400 MHz; $CDCl_3$)** δ 7.42 (s, 1H), 7.29 (d, J = 11.5 Hz, 1H), 7.19 (d, J = 11.5 Hz, 1H), 6.78 (d, J = 8.5 Hz, 1H), 6.72 (d, J = 8.5 Hz, 1H), 3.91 (s, 3H), 3.90 (s, 3H), 3.66 (s, 3H), 2.22 (s, 3H). **$^{13}C\{^1H\}$ NMR (101 MHz; $CDCl_3$)** δ 171.2, 168.8, 153.8, 150.8, 149.0, 142.5, 140.4, 139.5, 130.3, 126.0, 124.1, 122.6, 107.6, 61.23, 61.17, 56.3, 27.0. **HRMS (ESI+ TOF) *m/z*:** $(M+H)^+$ Calc'd for $C_{17}H_{19}O_5^+$: 303.1233. Found: 303.1233.

VIII. Methoxytropone Re-Methylation Synthesis and Characterization

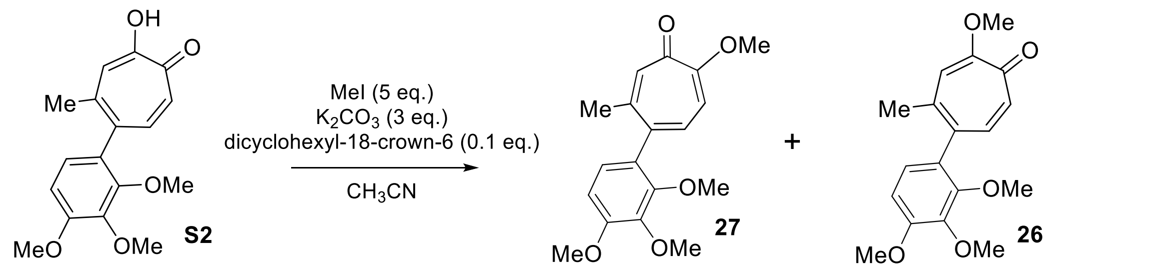

General Procedure:[15] To a flame dried microwave vial equipped with stir bar was added **18c** (1 eq.) in CH_3CN (0.2 M), K_2CO_3 (3 eq.), and dicylohexyl-18-crown-6 (0.1 eq.). The reaction vessel is sealed and purged with argon. Under argon, iodomethane is added via a syringe (5 eq.). The reaction mixture is heated at 82 °C for 24 hours in an oil bath. Upon completion, the reaction mixture is diluted with DCM, and washed with sodium hydroxide (2x), sodium carbonate (1x), water (1x), and brine (1x). The combined organics were dried with Na_2SO_4, filtered, and concentrated *en vacuo*. The resulting oil is then purified by chromatography (Biotage Isolera Prime, SiliCycle SiliaSep 10 g silica gel, 40-63 μm 60 Å, solvent gradient: 0-100% acetonitrile in dichloromethane (500 mL). Product fractions were concentrated *en vacuo* to yield a mixture of tropones **27** and **26**. A chiral Diacel IA column in 2-propanol/hexanes (10-100%) was used for resolution of the enantiomers.

2-methoxy-6-methyl-5-(2,3,4-trimethoxyphenyl)cyclohepta-2,4,6-trien-1-one (27)

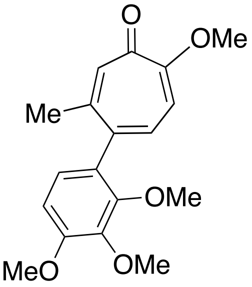

Off-white solid **27** (2.6 mg, 15% yield) was obtained from tropolone **S2** (16 mg, 0.055 mmol, 1 eq.), K_2CO_3 (22.8 mg, 0.165 mmol, 3 eq), and dicylohexyl-18-crown-6 (2 mg, 0.005 mmol, 0.10 eq.) and MeI (17.1 μL, 0.275 mmol, 5 eq.) in CH_3CN (291 μL, 0.2 M) at 82 °C after 24 hours to yield a mixture of **27** and **26** as oil (5.9 mg, 34% combined yield). A chiral Daicel IA column in Hex/2-propanol (10 – 100%) separated the isomers to yield **27** as a solid (2.6 mg, 15% yield). **m.p.** = 196 –198 °C. $\mathbf{R_f}$ = 0.20 in 60 % ethyl acetate: hexane. **IR (ATR, ZnSe):** 2941 (w), 2837 (w), 1659 (w), 1618 (w), 1589 (m), 1567 (s), 1491 (m), 1461 (m), 1432 (m), 1409 (s), 1301 (m), 1250 (s), 1205 (m), 1159 (m), 1093 (s), 1068 (s), 1028 (w) 1011 (m), 996 (m), 977 (m), 916 (m), 850 (m), 808 (s), 795 (m) cm^{-1}. **^{1}H NMR (400 MHz, CDCl$_3$)** δ 7.34 (d, *J* = 0.2 Hz, 1H), 6.93 (d, *J* = 10.4 Hz, 1H), 6.80 (d, *J* = 8.5 Hz, 1H), 6.71 (d, *J* = 8.6 Hz, 1H), 6.65 (d, *J* = 10.5 Hz, 1H), 3.95 (s, 3H), 3.90 (s, 6H), 3.69 (s, 3H), 2.09 (d, *J* = 0.8 Hz, 3H). **^{13}C{^{1}H} NMR (101 MHz, CDCl$_3$)** δ 179.4, 163.7, 153.9, 150.9, 148.7, 142.3, 141.1, 137.8, 132.7, 130.2, 124.1, 111.3, 107.4, 61.2, 61.1, 56.3, 56.2, 26.8. **HRMS (ESI+ TOF) *m/z*:** $(M+H)^+$ Calc'd for $C_{18}H_{20}O_5^+$: 317.1389. Found: 317.1386.

IX. Biological and Biochemical Assessment of Analogs 26 and 27

1. Binding affinity to the colchicine site of bovine brain tubulin (PurSolutions, LLC (puresoluble.com)) was assayed by competition of test compounds with MDL ((E)-1-(2,5-dimethoxyphenyl)-3-[4-(dimethylamino)phenyl]-2-methylprop-2-en-1-one) as described in [PMID 35712668]. MDL fluorescence increases many-fold upon binding to the colchicine site of tubulin. Inhibition of this fluorescence increase was measured and converted to a Kd for the test compound as described.

2. Inhibition of tubulin polymerization was determined as described in [PMID 23973075]. In brief, 10 μM bovine brain tubulin (PurSolutions, LLC (puresoluble.com)) was incubated under conditions strongly promoting polymerization of tubulin (1M NaGlutamate, 0.1 M Mes (Morpholinoethanesulfonic acid), 1 mM MgCl2, 0.5 mM GTP, pH 6.9), in the absence or presence of 3.3 or 33 μM test compound, incubated for 30 min at 37 °C, then centrifuged at 100,000 x g for 8', and the top ¾ of the supernatant removed. Protein concentration was measured, and polymerization was determined as pelletable protein lost from the supernatant.

3. Inhibition of cell growth was determined using standard procedures. Cell lines were obtained from the NCI anticancer drug screen, and maintained in DMEM medium supplemented with 10% fetal bovine serum. Growing cells were exposed to serial dilutions of each compound for 3 days. Cell growth was determined with CellTiter Assay Reagent (Promega), and growth parameters, including inhibition of growth, were measured using the methods specified by the manufacturer.

X. NMR Spectra

^{1}H NMR (400 MHz, CD_3CN) of 4b

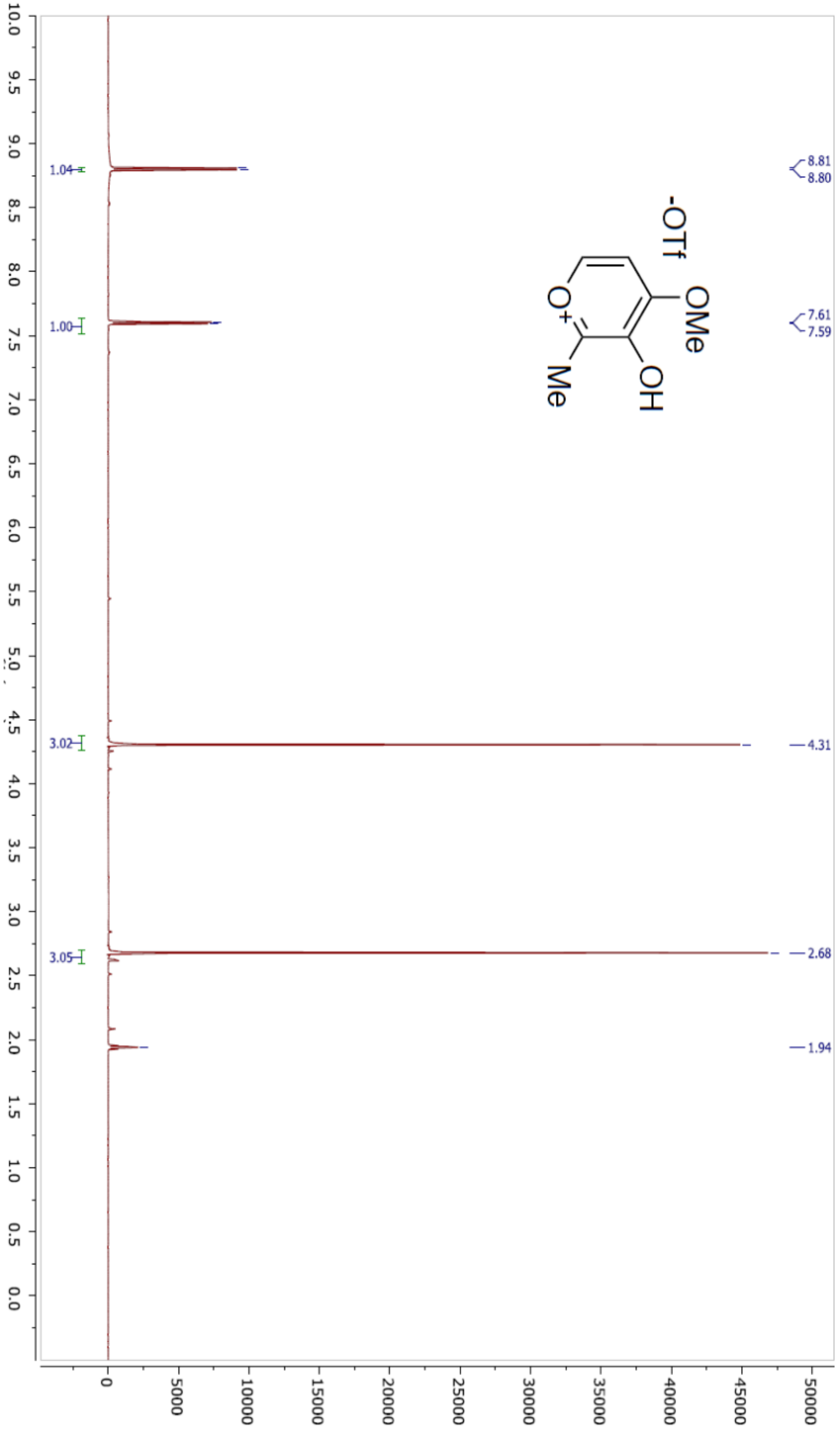

$^{13}C\{^1H\}$ NMR (101 MHz, CD_3CN) of 4b

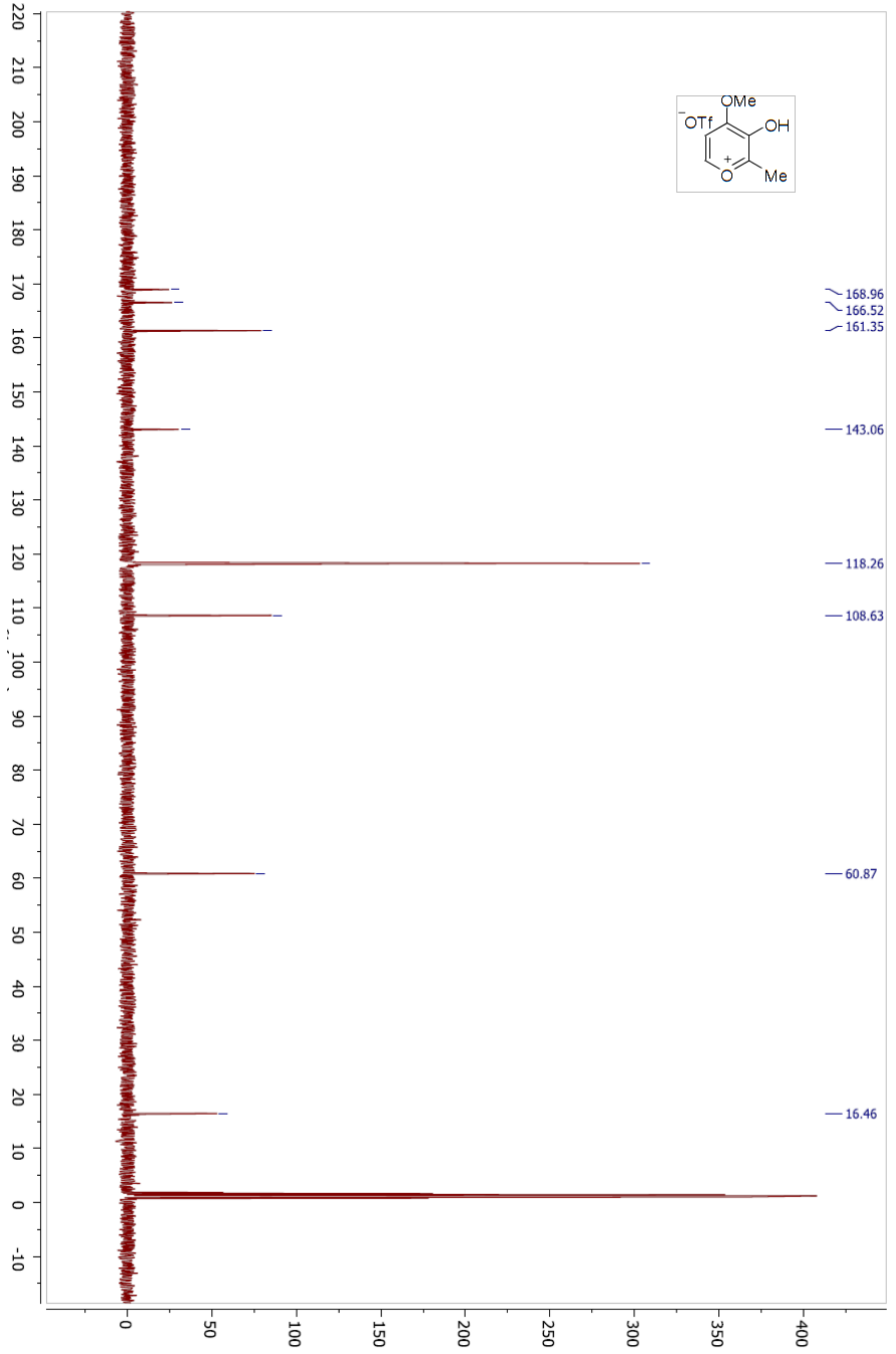

^{1}H NMR (400 MHz, DMSO-*d6*) of 4c

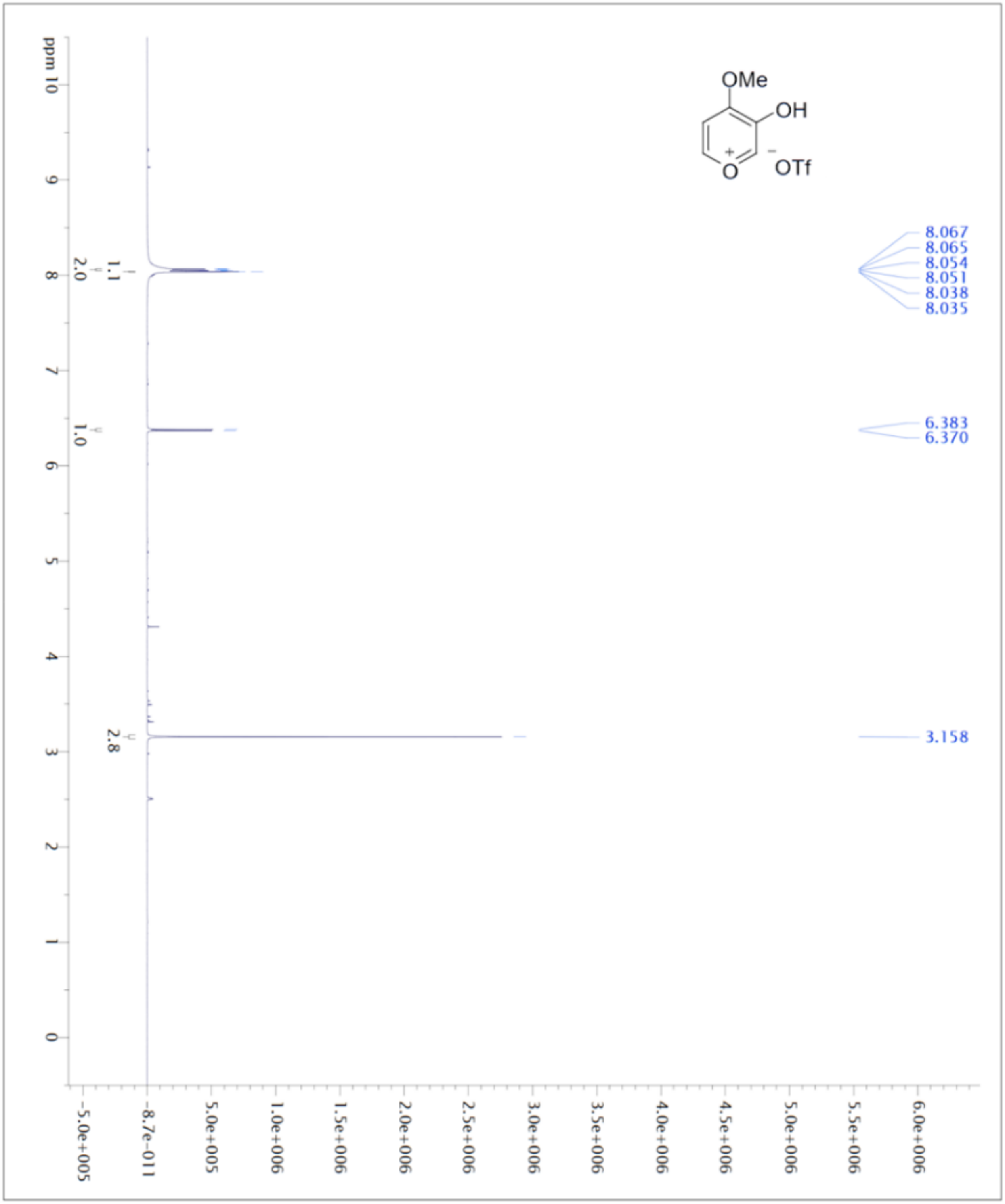

^{1}H NMR (400 MHz, CDCl$_3$) of 13b

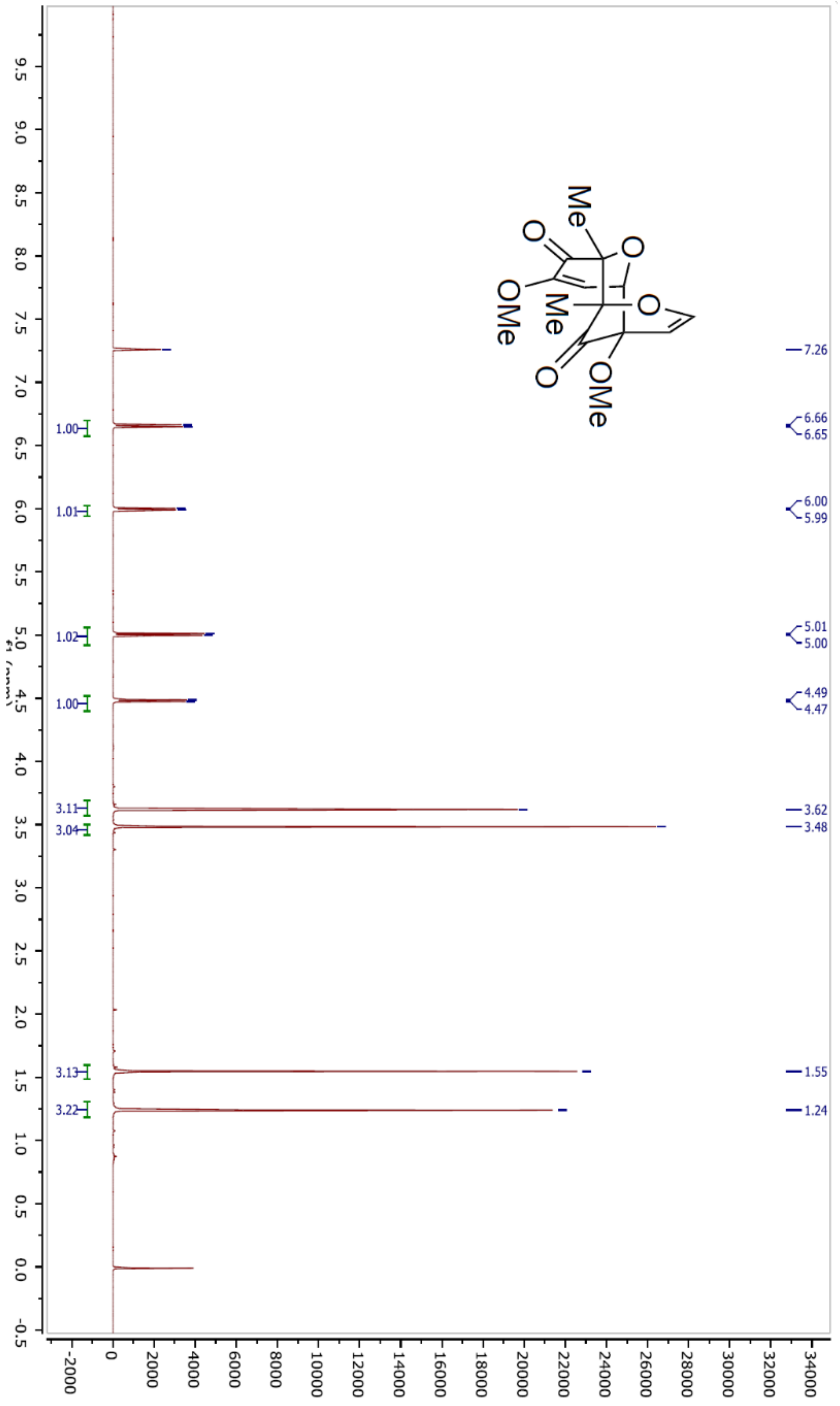

$^{13}C\{^1H\}$ NMR (101 MHz, $CDCl_3$) of 13b

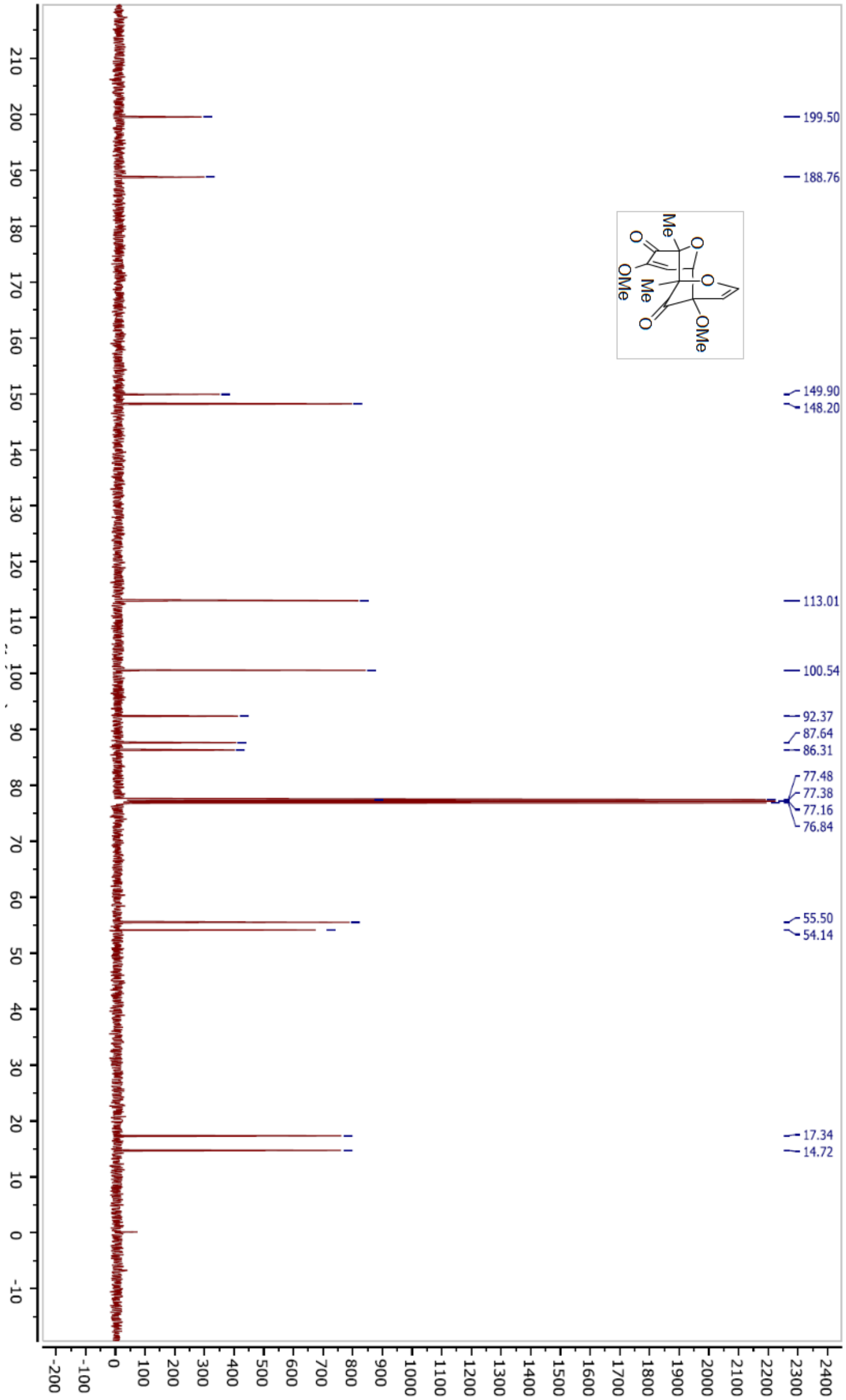

^{1}H NMR (400 MHz, $CDCl_3$) of 13b’

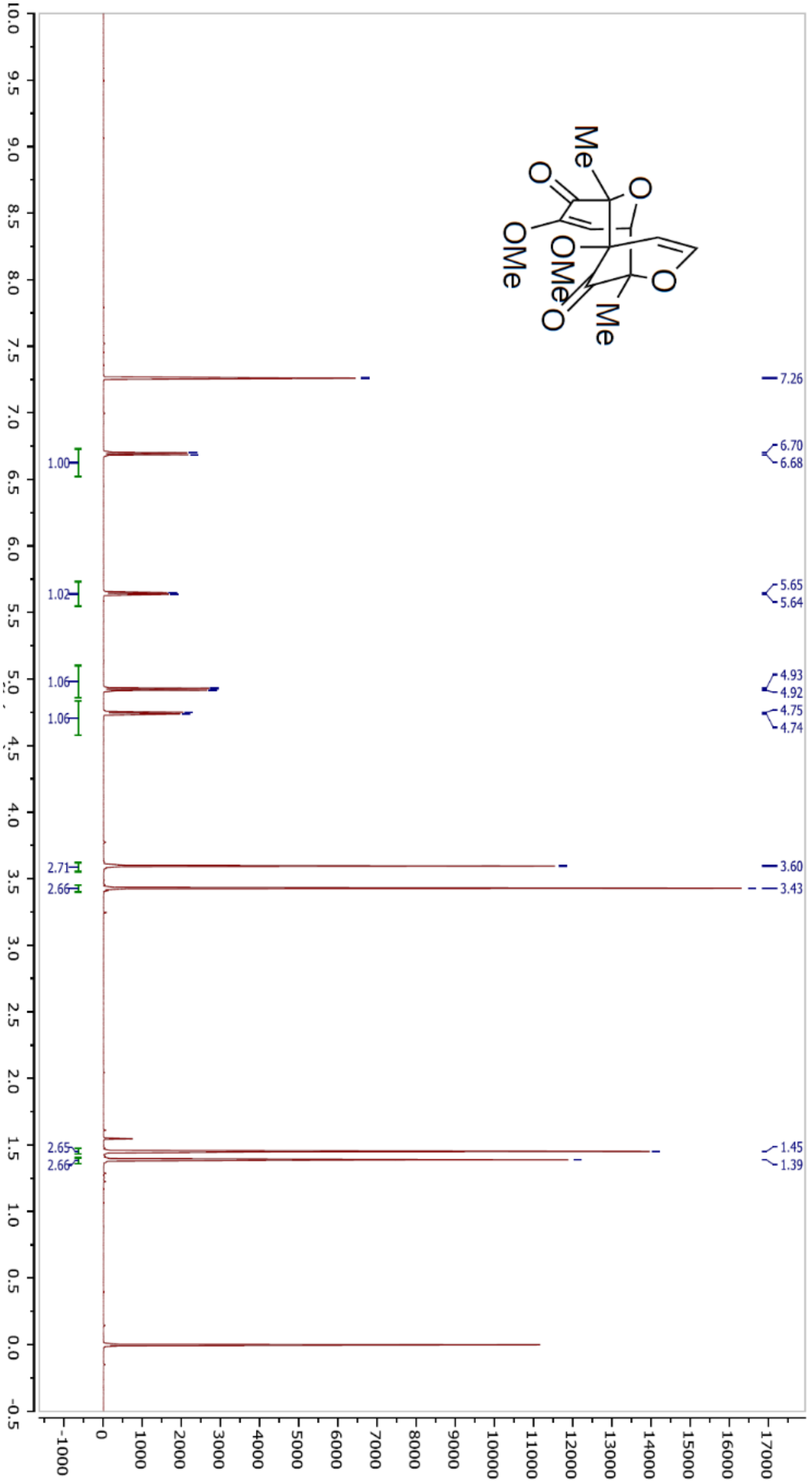

$^{13}C\{^{1}H\}$ NMR (101 MHz, $CDCl_3$) of 13b’

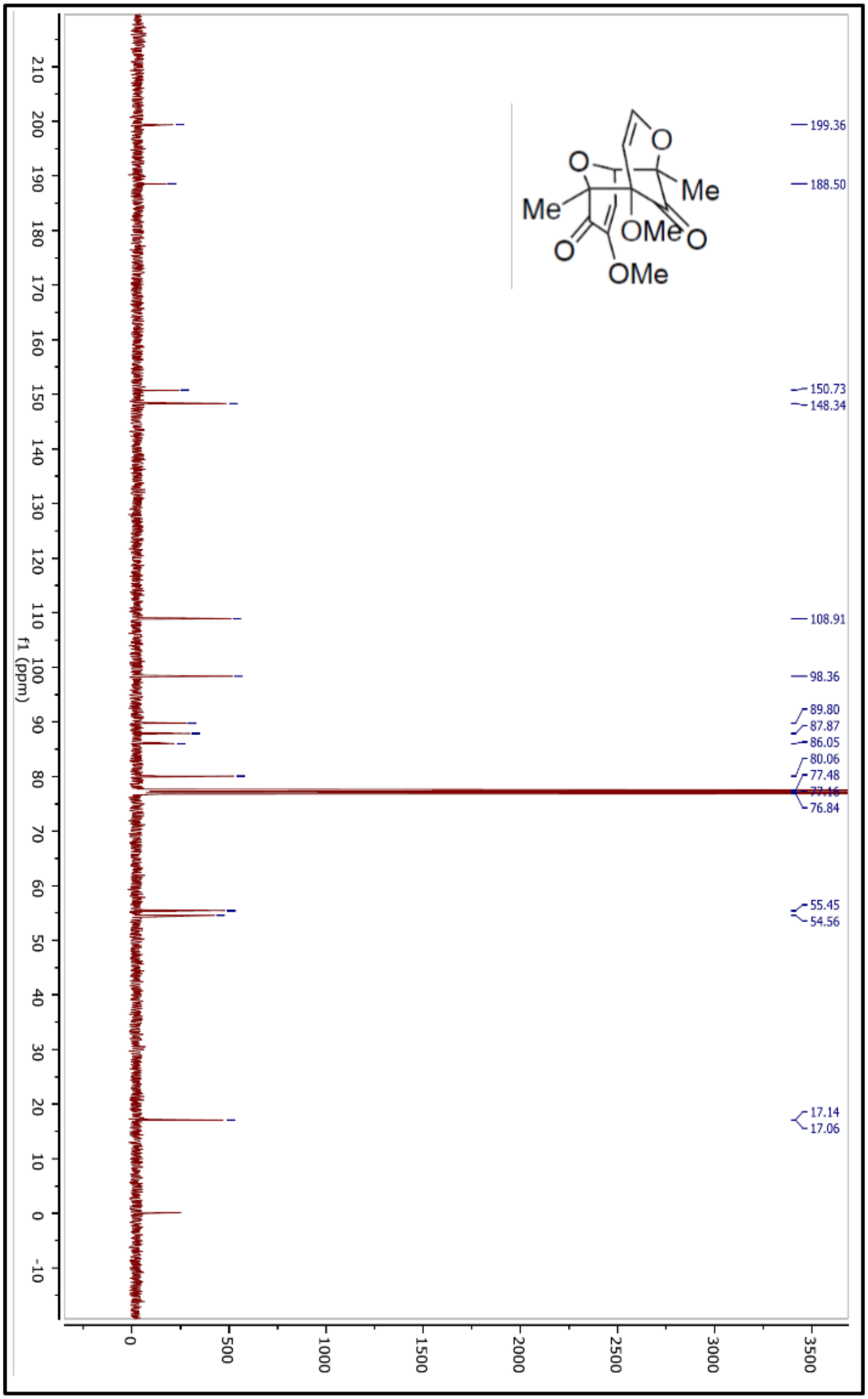

^{1}H NMR (400 MHz, $CDCl_3$) of 13c

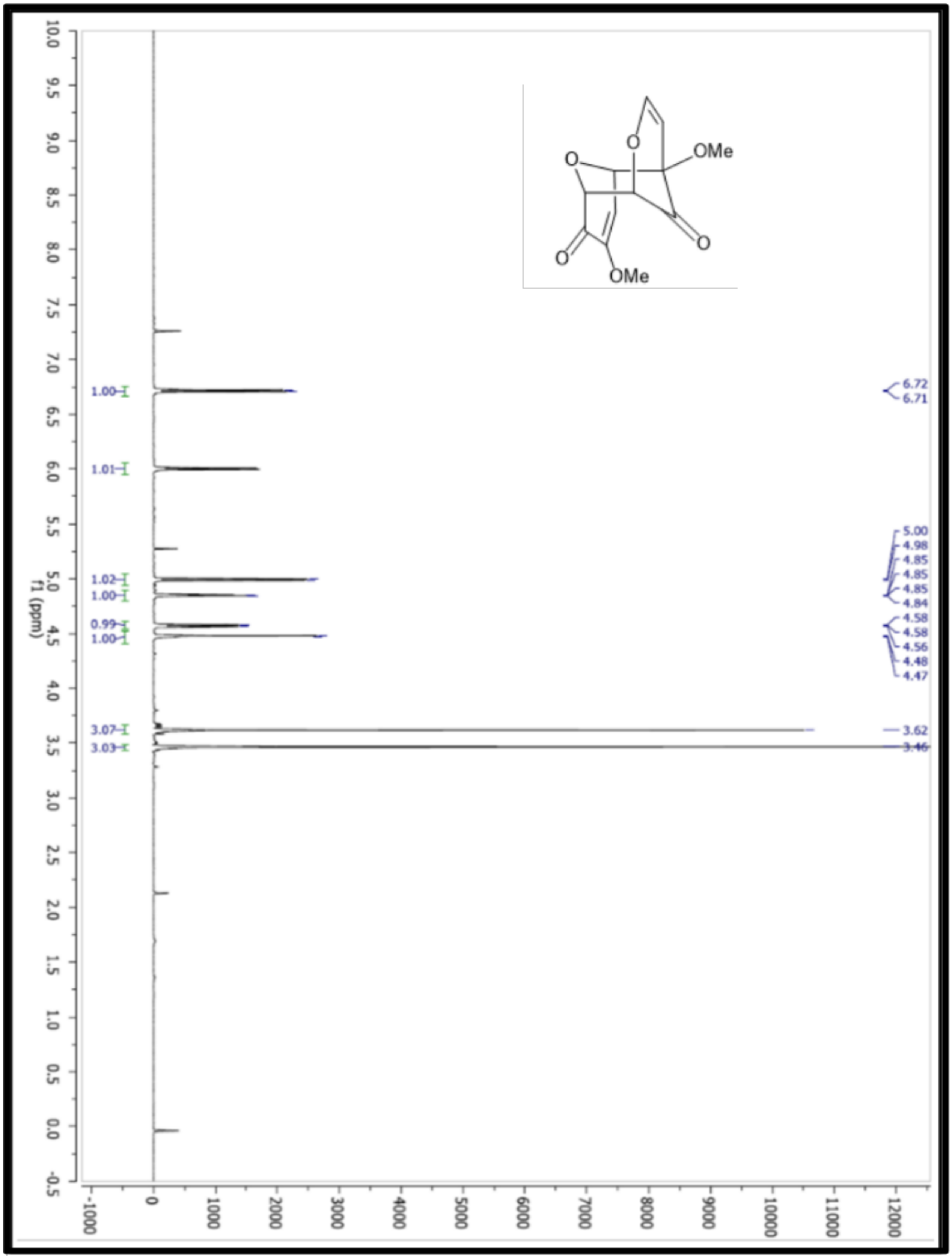

$^{13}C\{^1H\}$ NMR (101 MHz, $CDCl_3$) of 13c

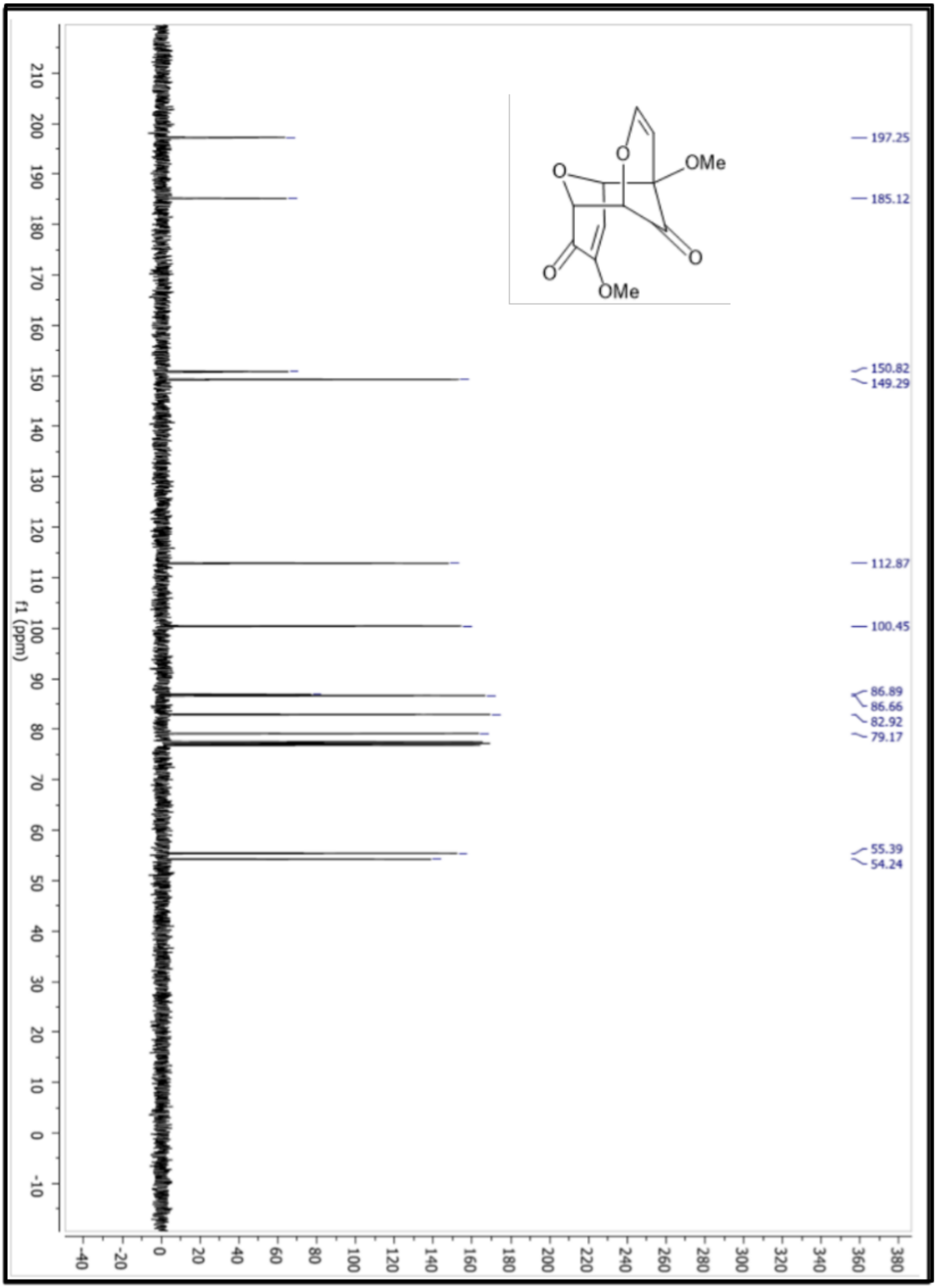

^{1}H NMR (400 MHz, $CDCl_3$) of 6c

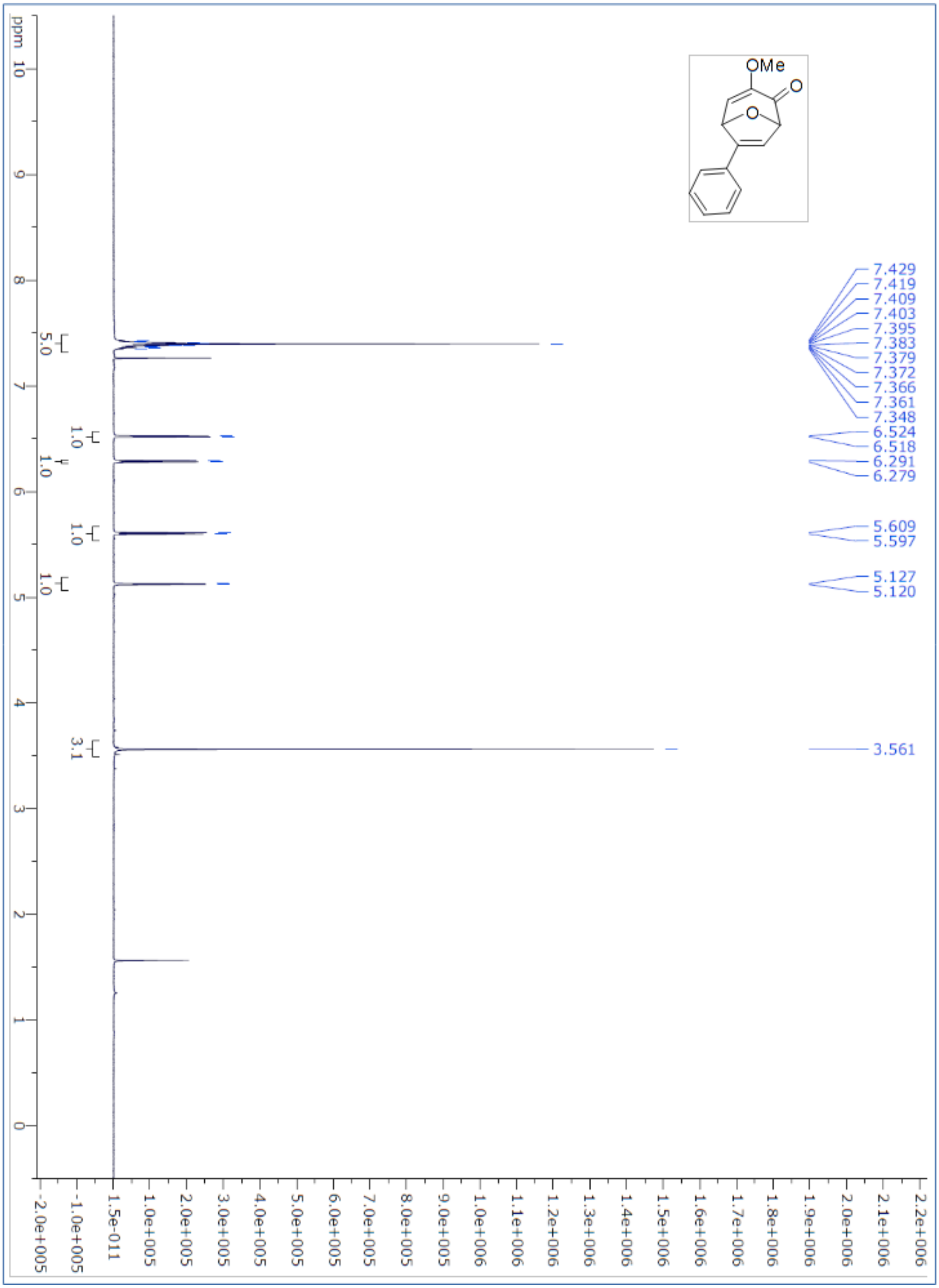

$^{13}C\{^{1}H\}$ NMR (101 MHz, $CDCl_3$) of 6c

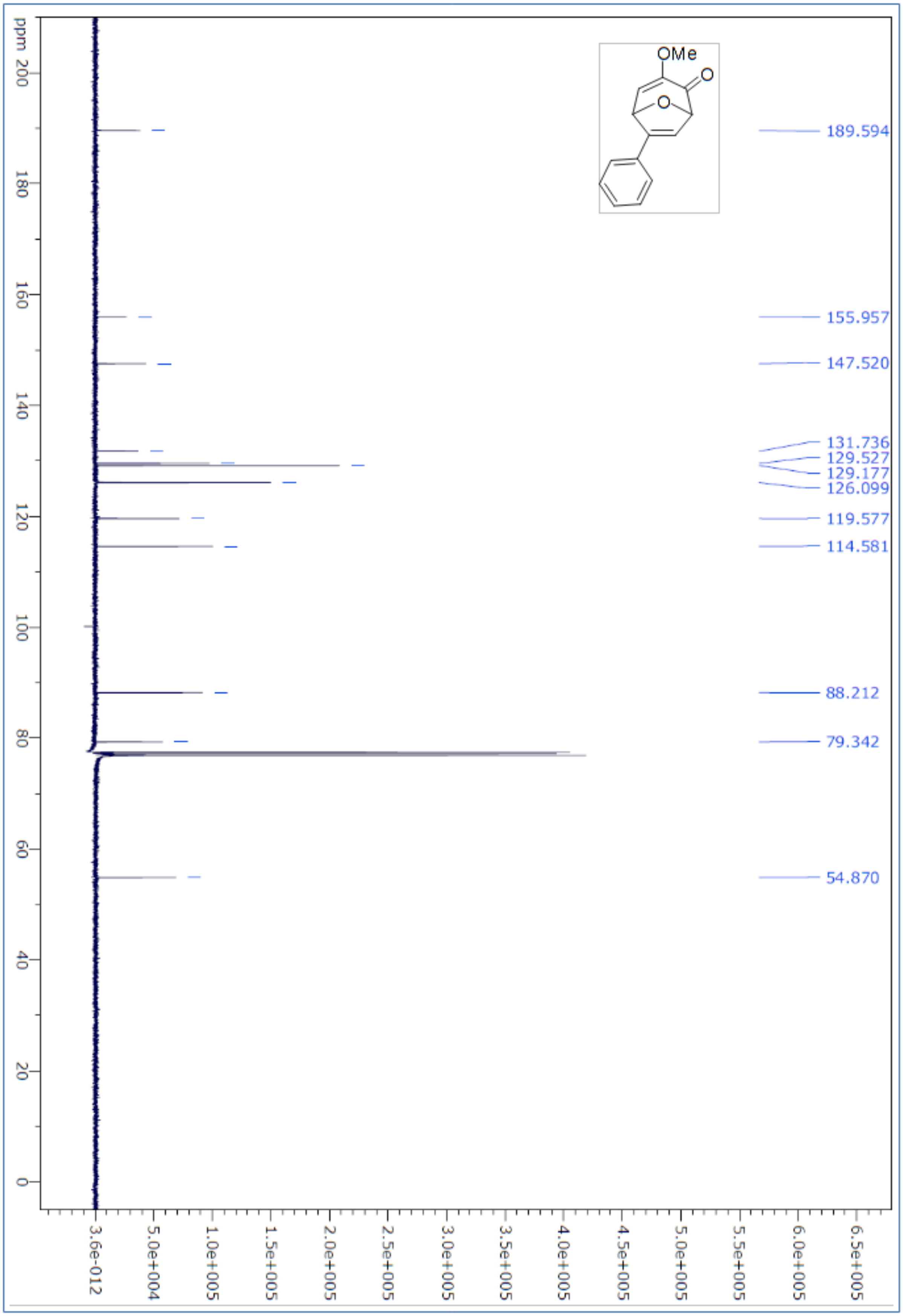

^{1}H NMR (400 MHz, $CDCl_3$) of 6g

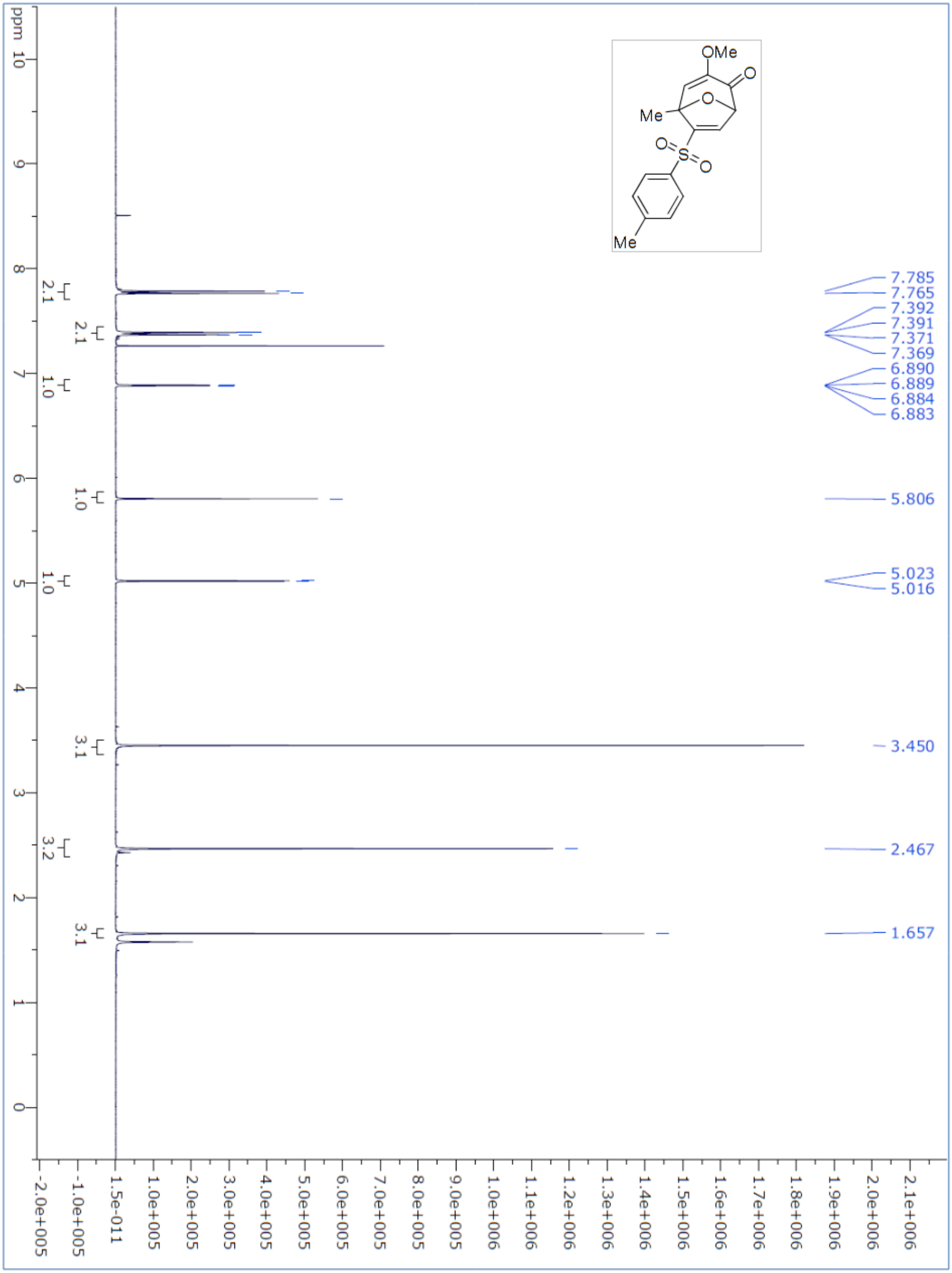

$^{13}C\{^{1}H\}$ NMR (101 MHz, $CDCl_3$) of 6g

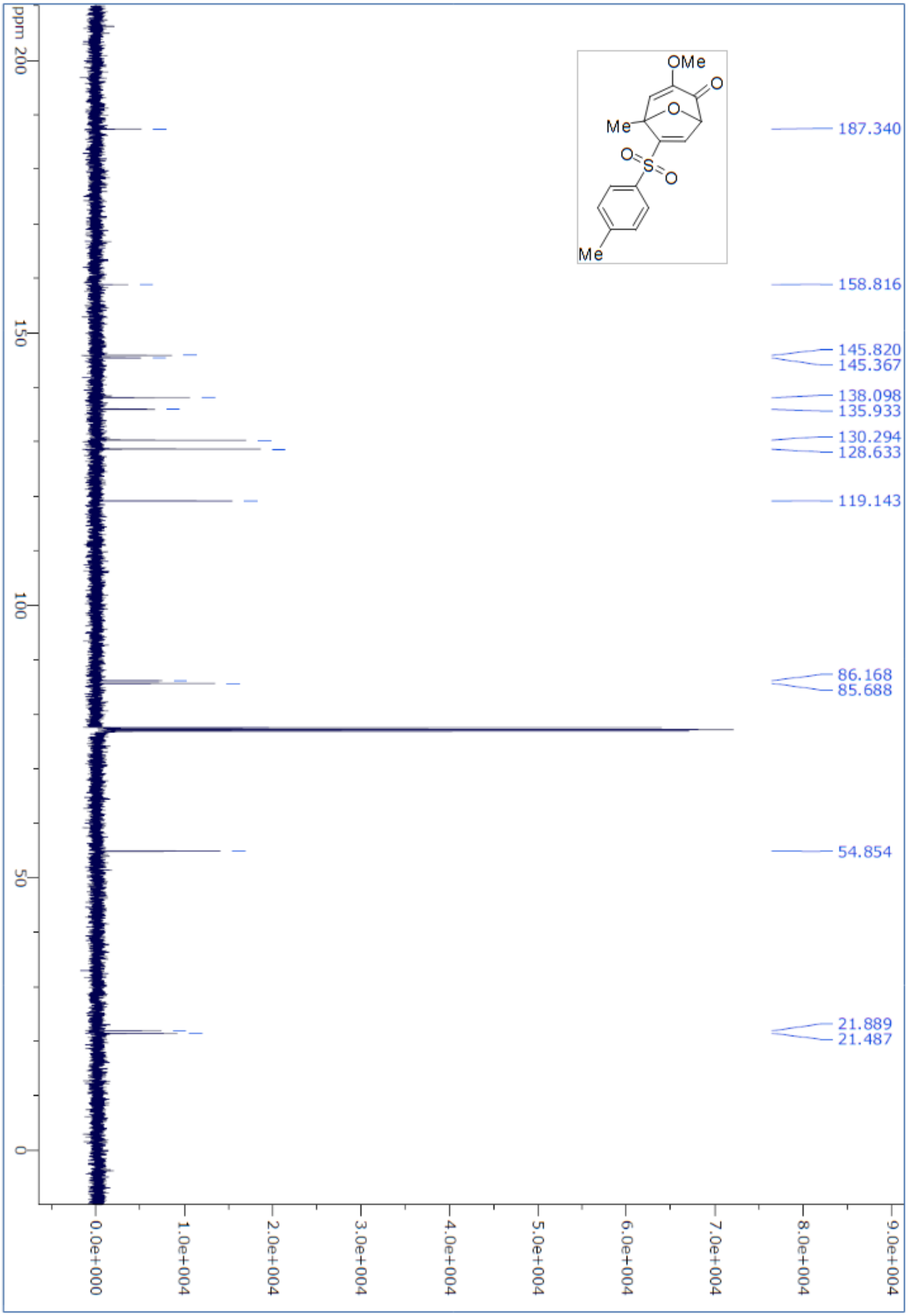

^{1}H NMR (400 MHz, $CDCl_3$) of 6h

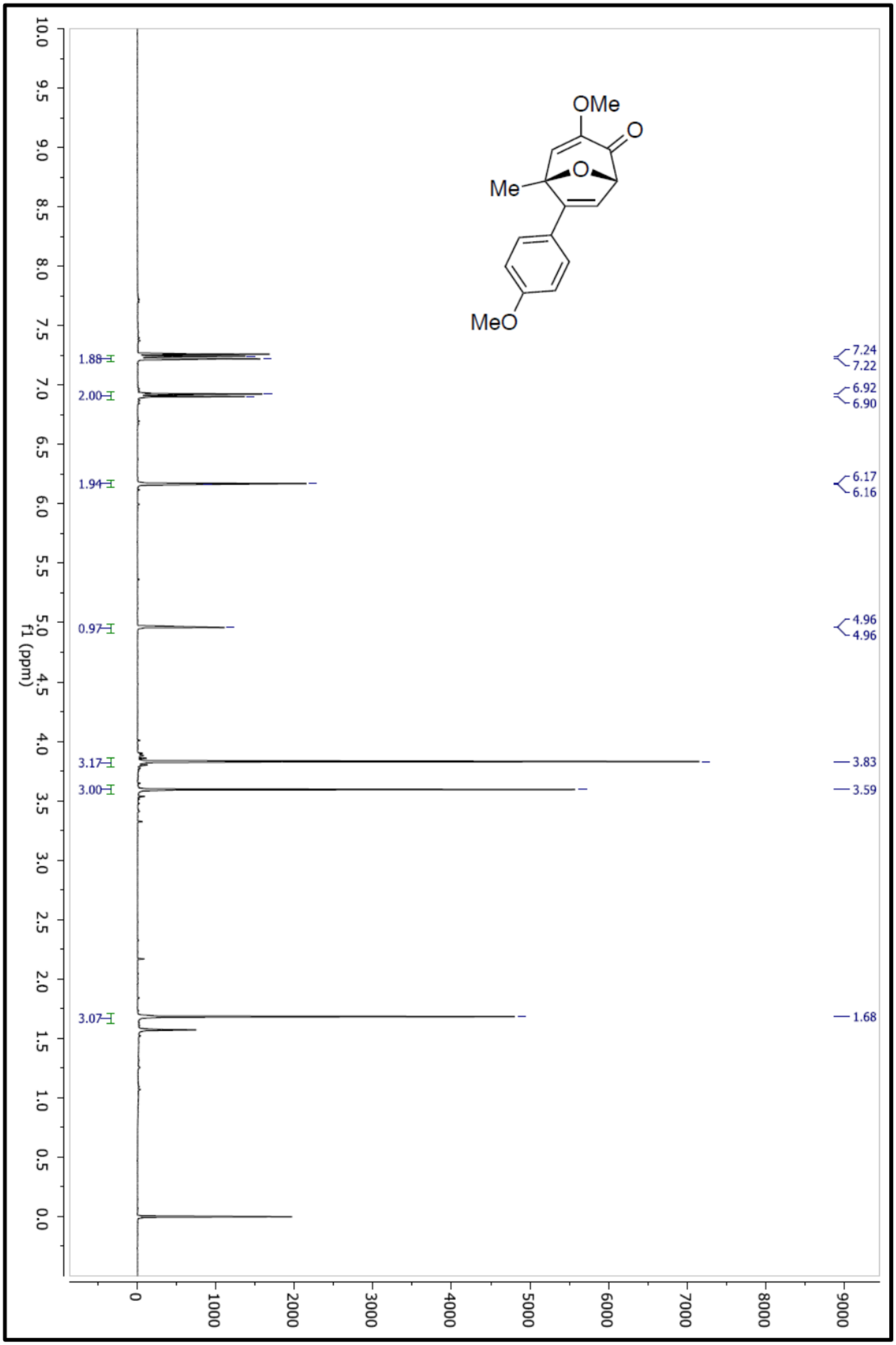

$^{13}C\{^{1}H\}$ NMR (101 MHz, $CDCl_3$) of 6h

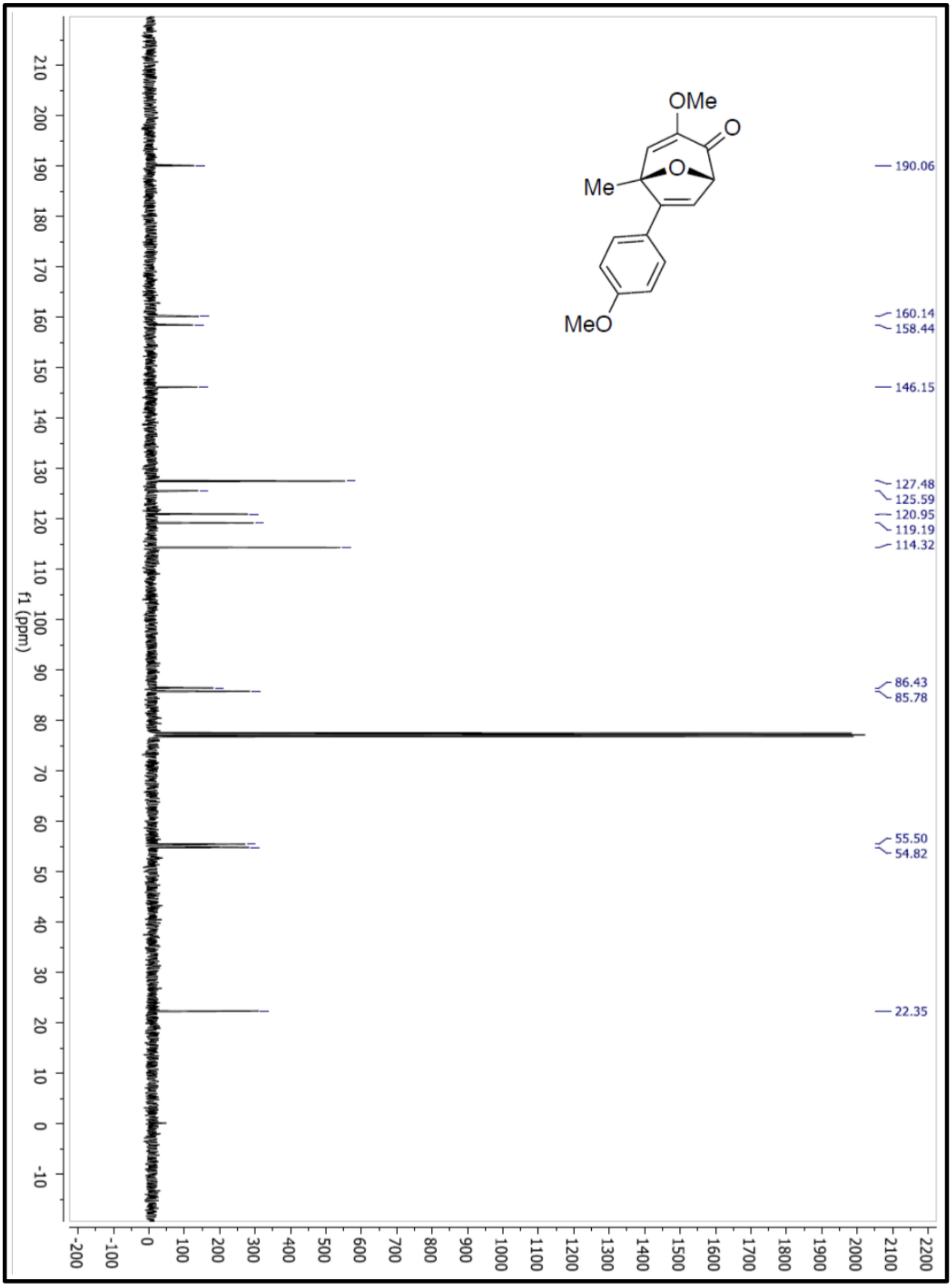

^{1}H NMR (400 MHz, $CDCl_3$) of 6j

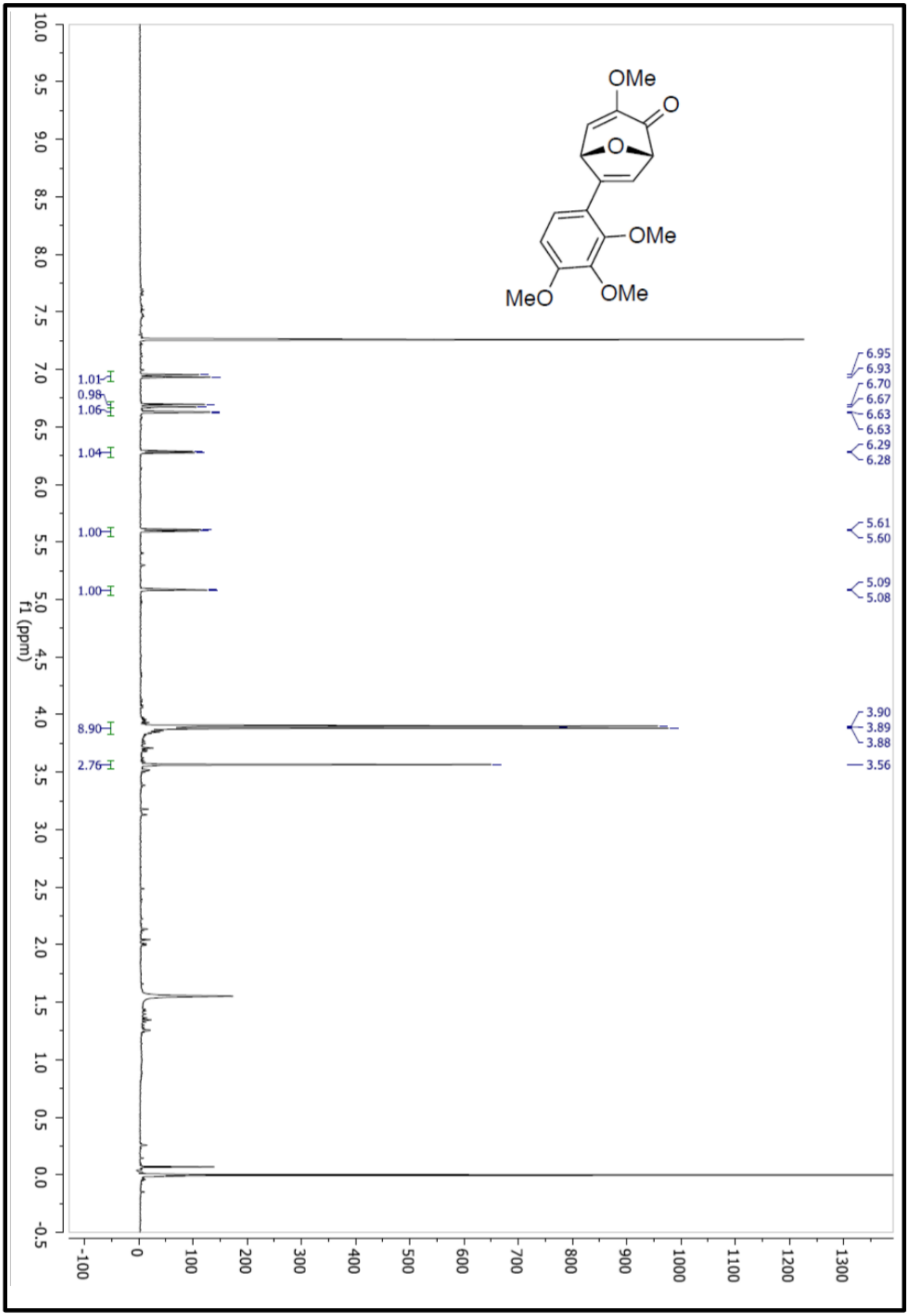

$^{13}C\{^1H\}$ NMR (101 MHz, $CDCl_3$) of 6j

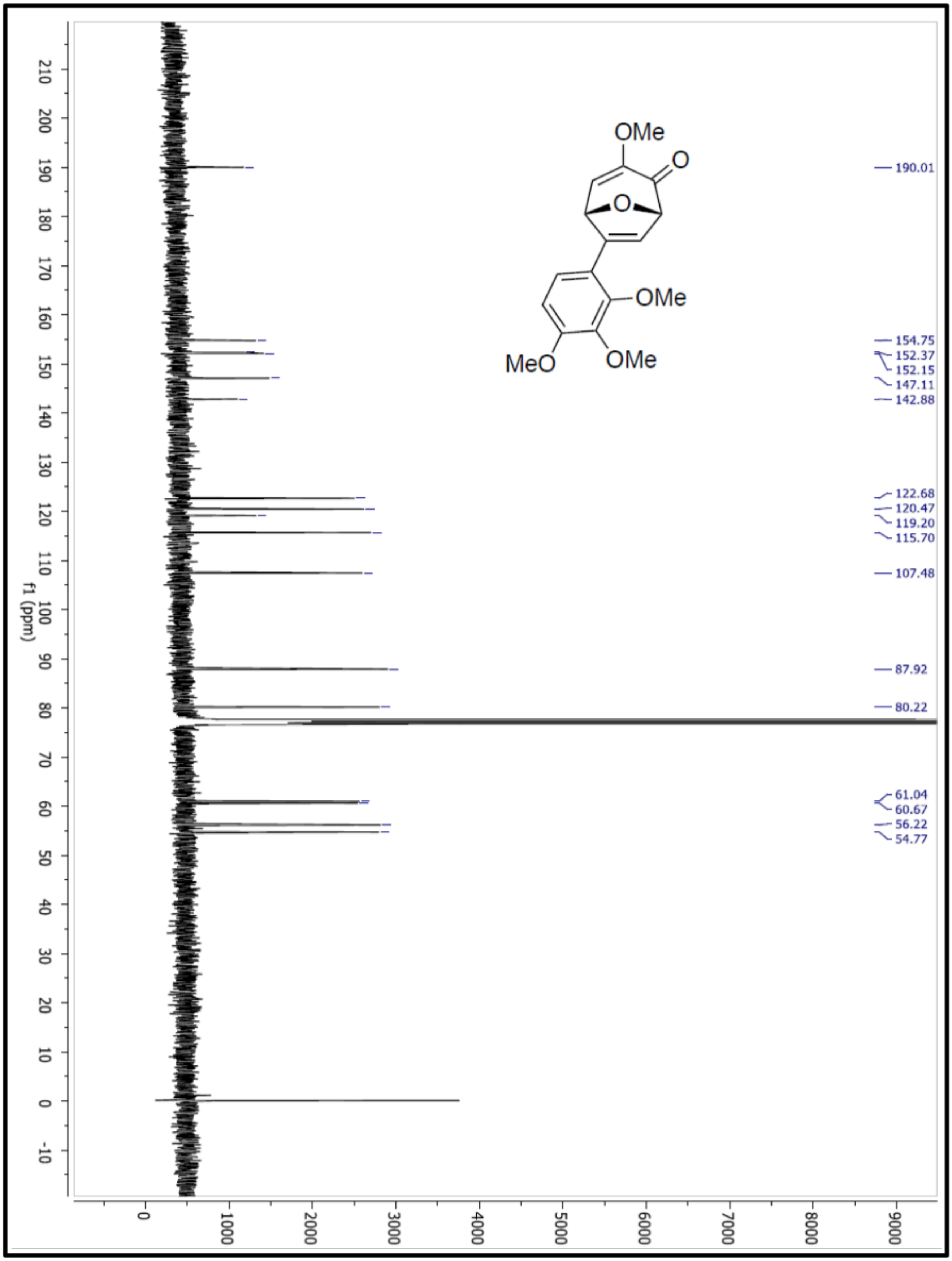

^{1}H NMR (400 MHz, CDCl$_3$) of 6l

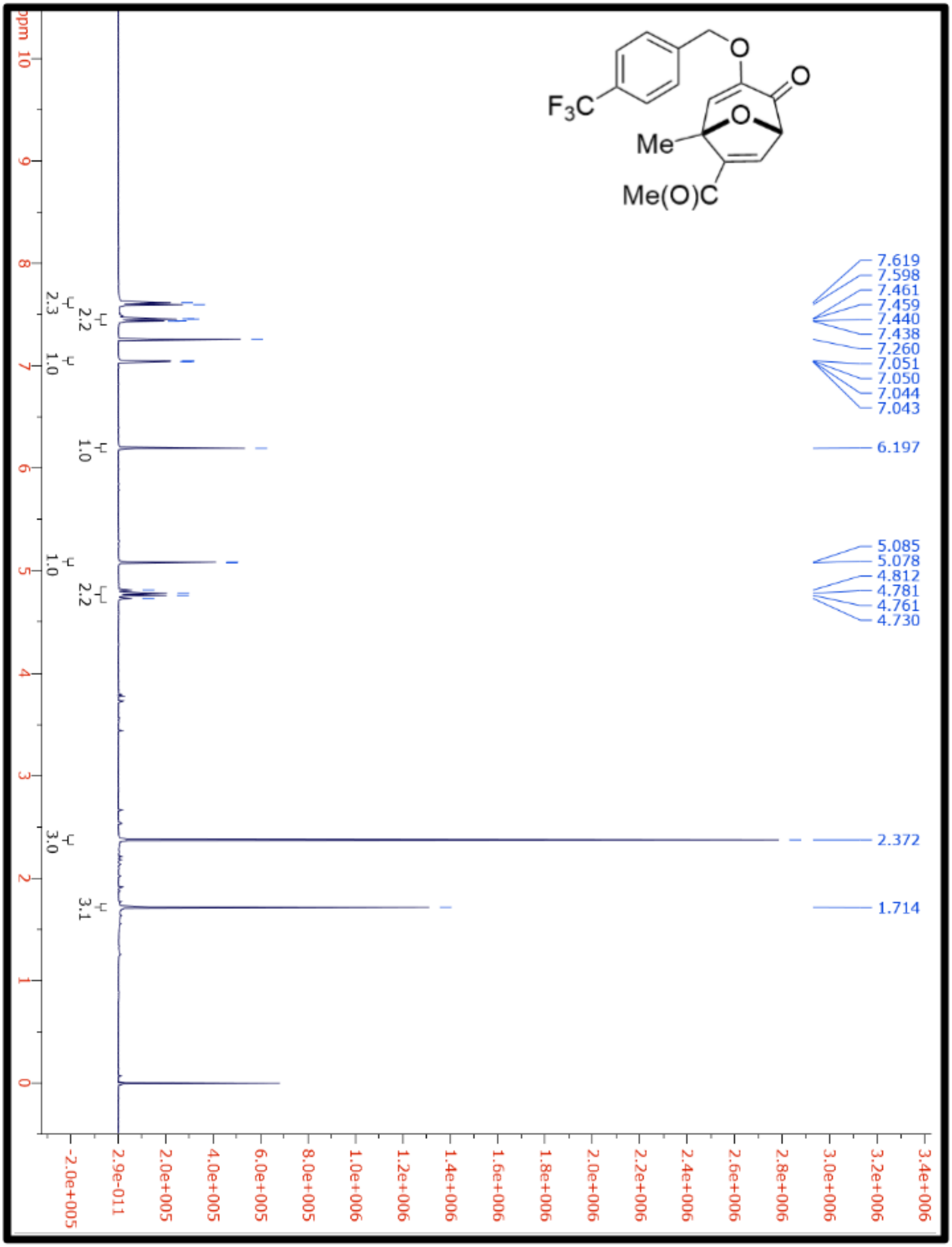

$^{13}C\{^{1}H\}$ NMR (101 MHz, $CDCl_3$) of 6l

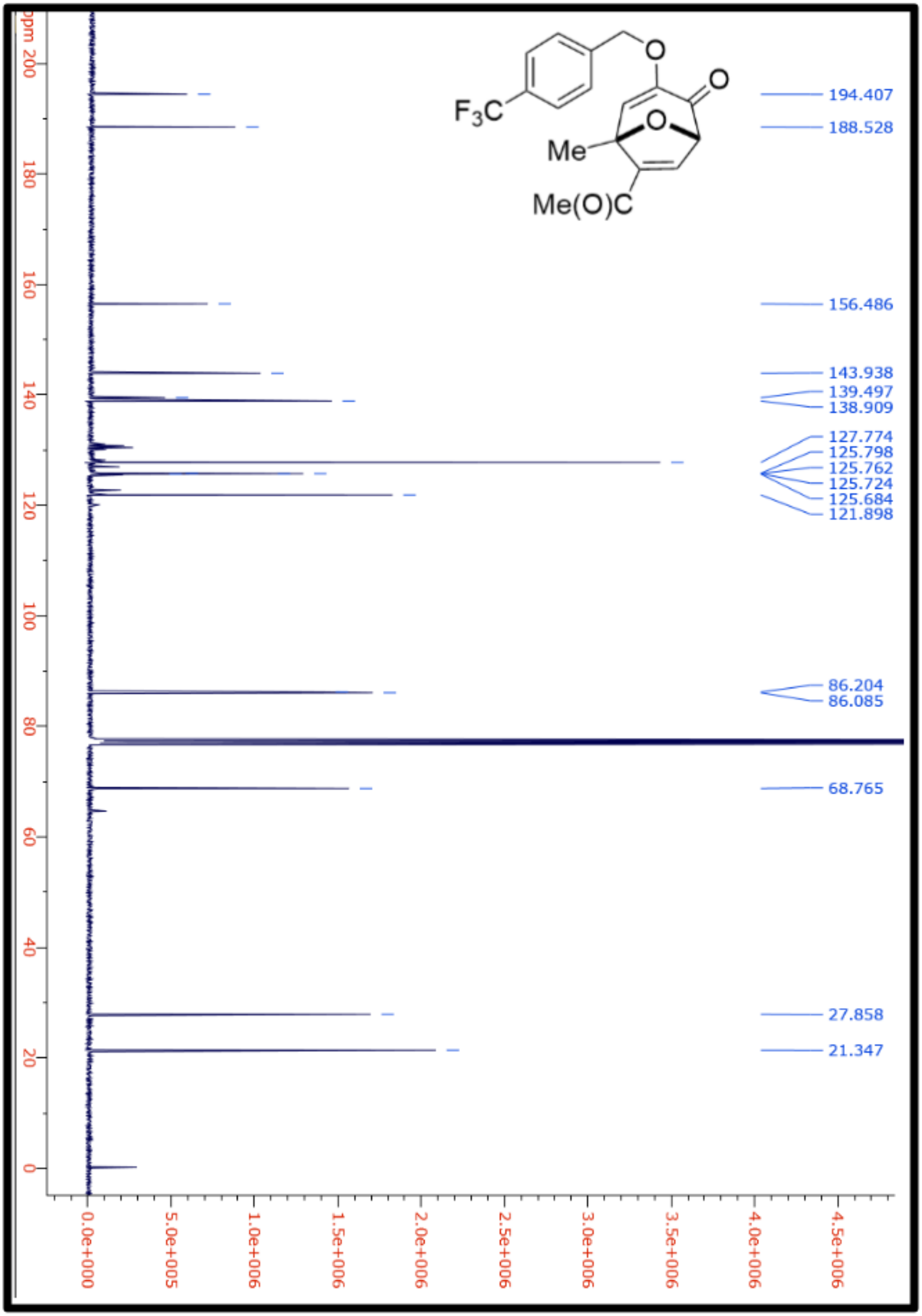

^{1}H NMR (400 MHz, CDCl3) of 8a

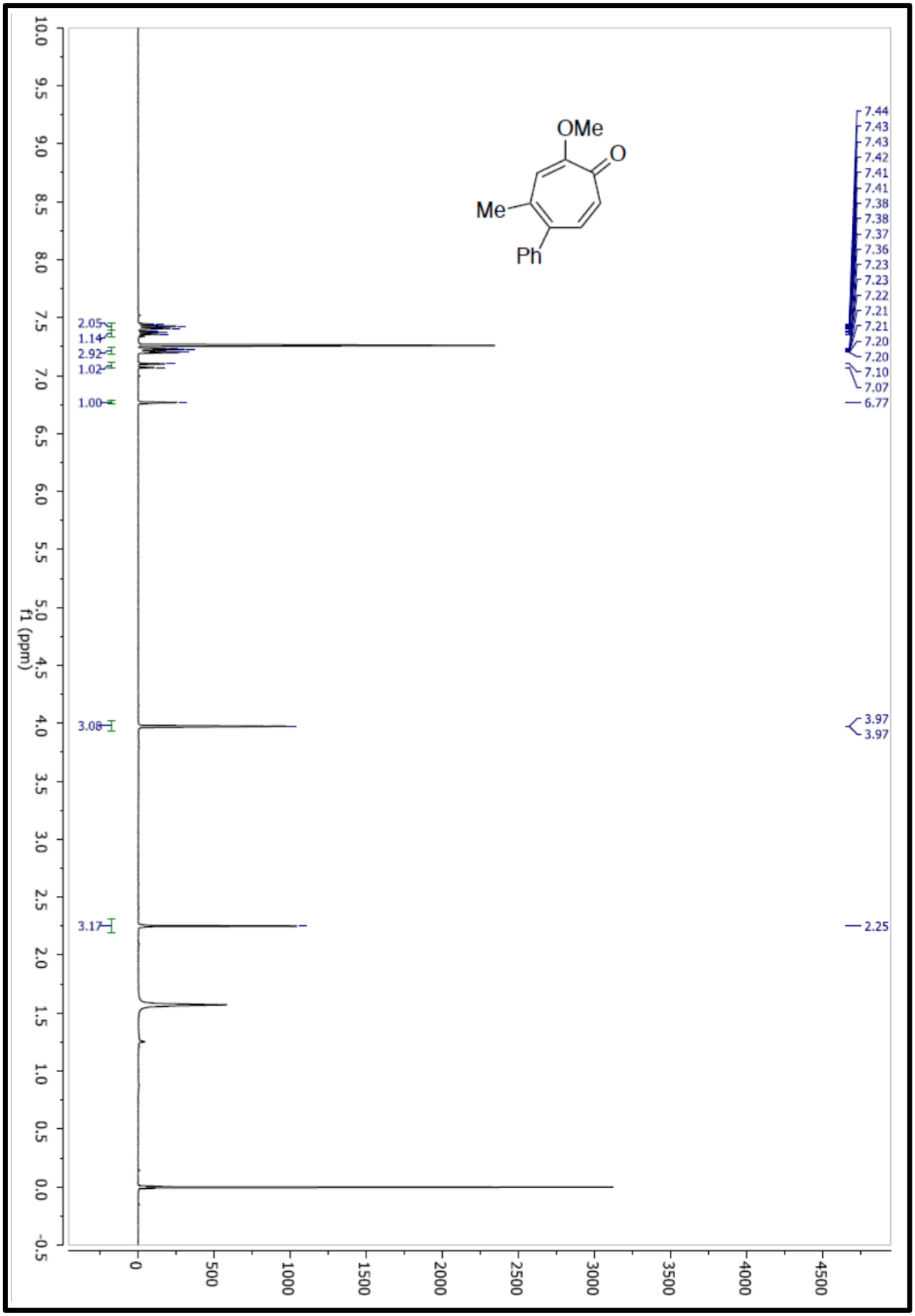

$^{13}C\{^1H\}$ NMR (101 MHz, $CDCl_3$) of 8a

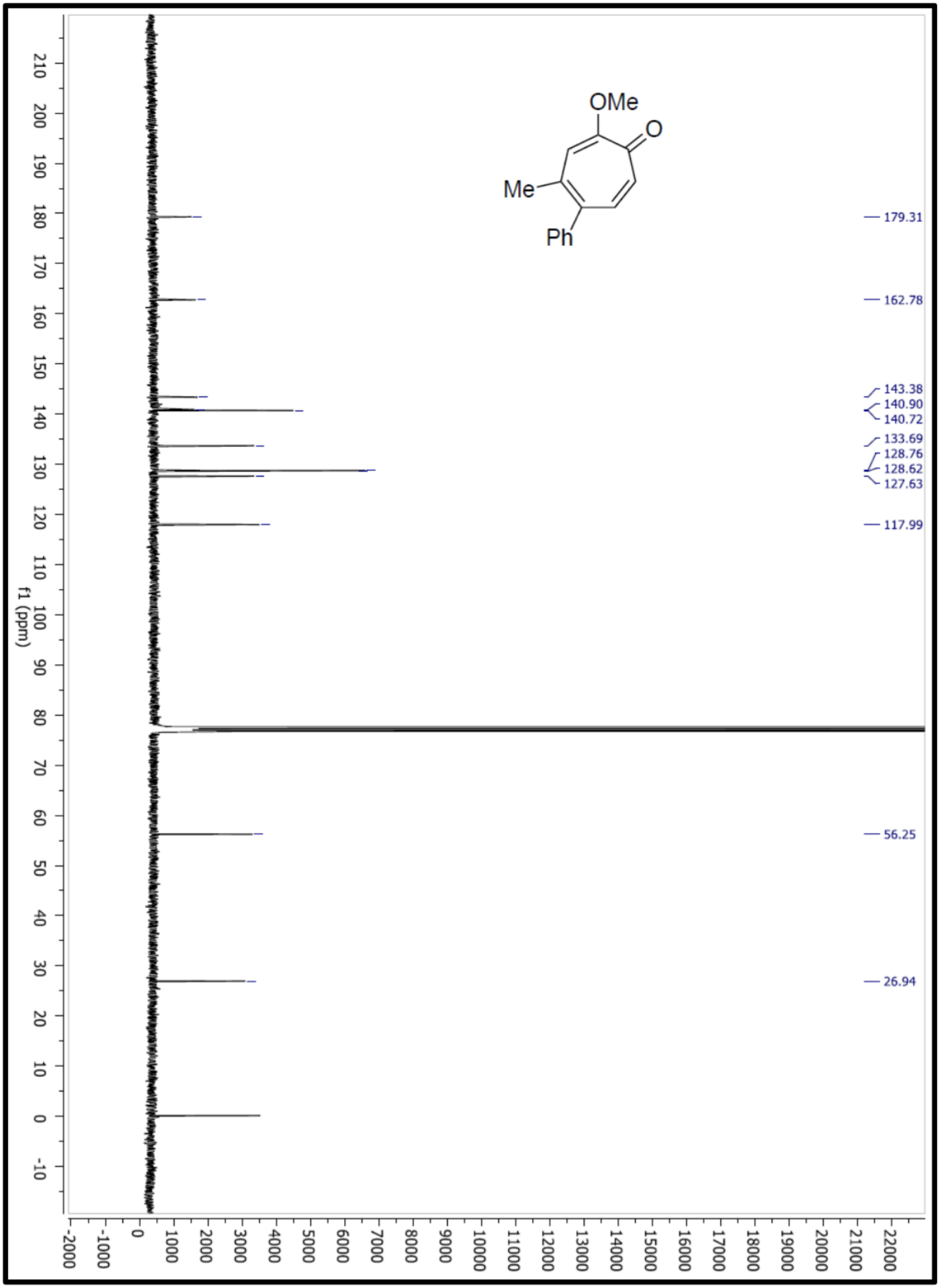

^{1}H NMR (400 MHz, $CDCl_3$) of 8b

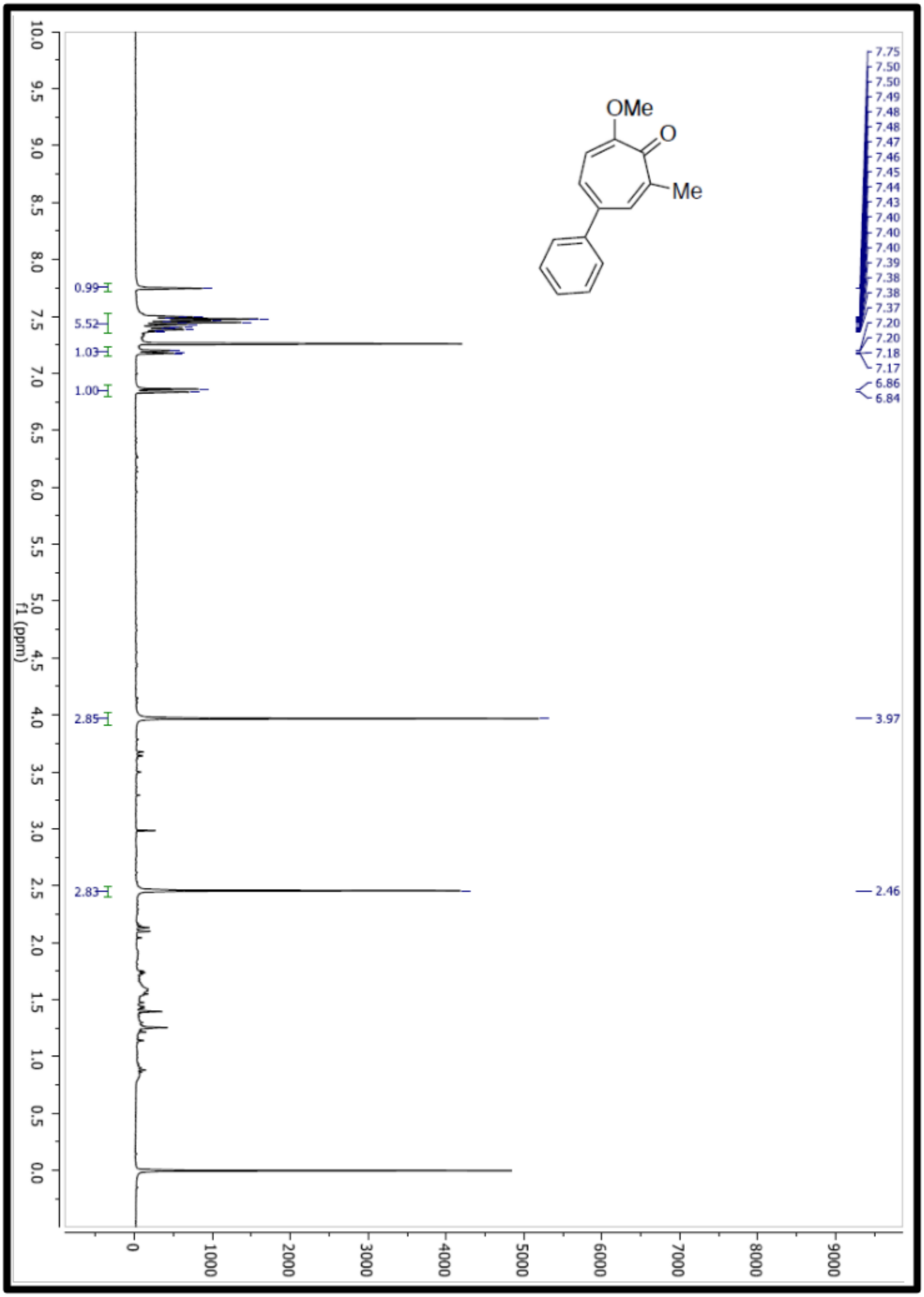

$^{13}C\{^{1}H\}$ NMR (101 MHz, $CDCl_3$) of 8b

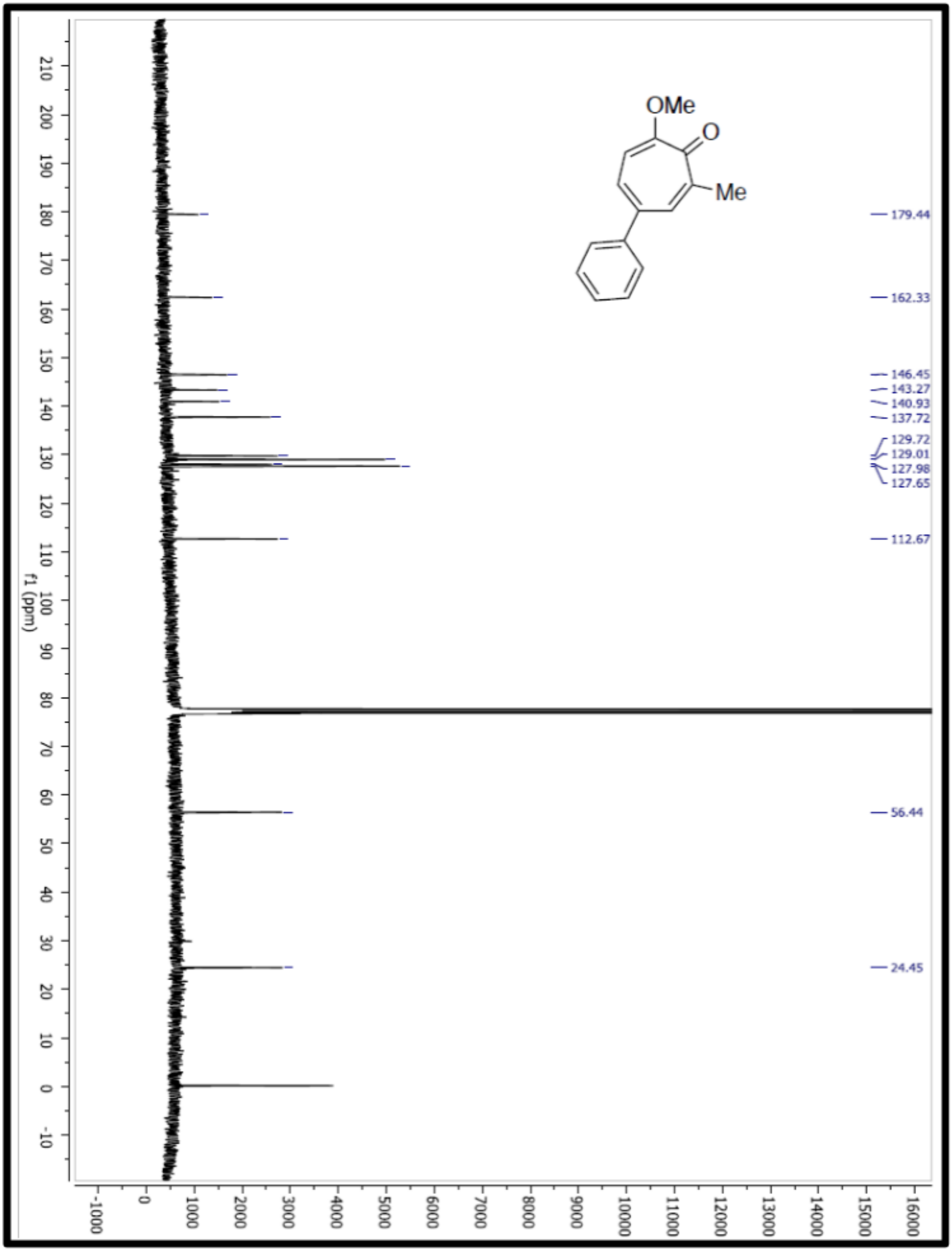

^{1}H NMR (400 MHz, $CDCl_3$) of 8c

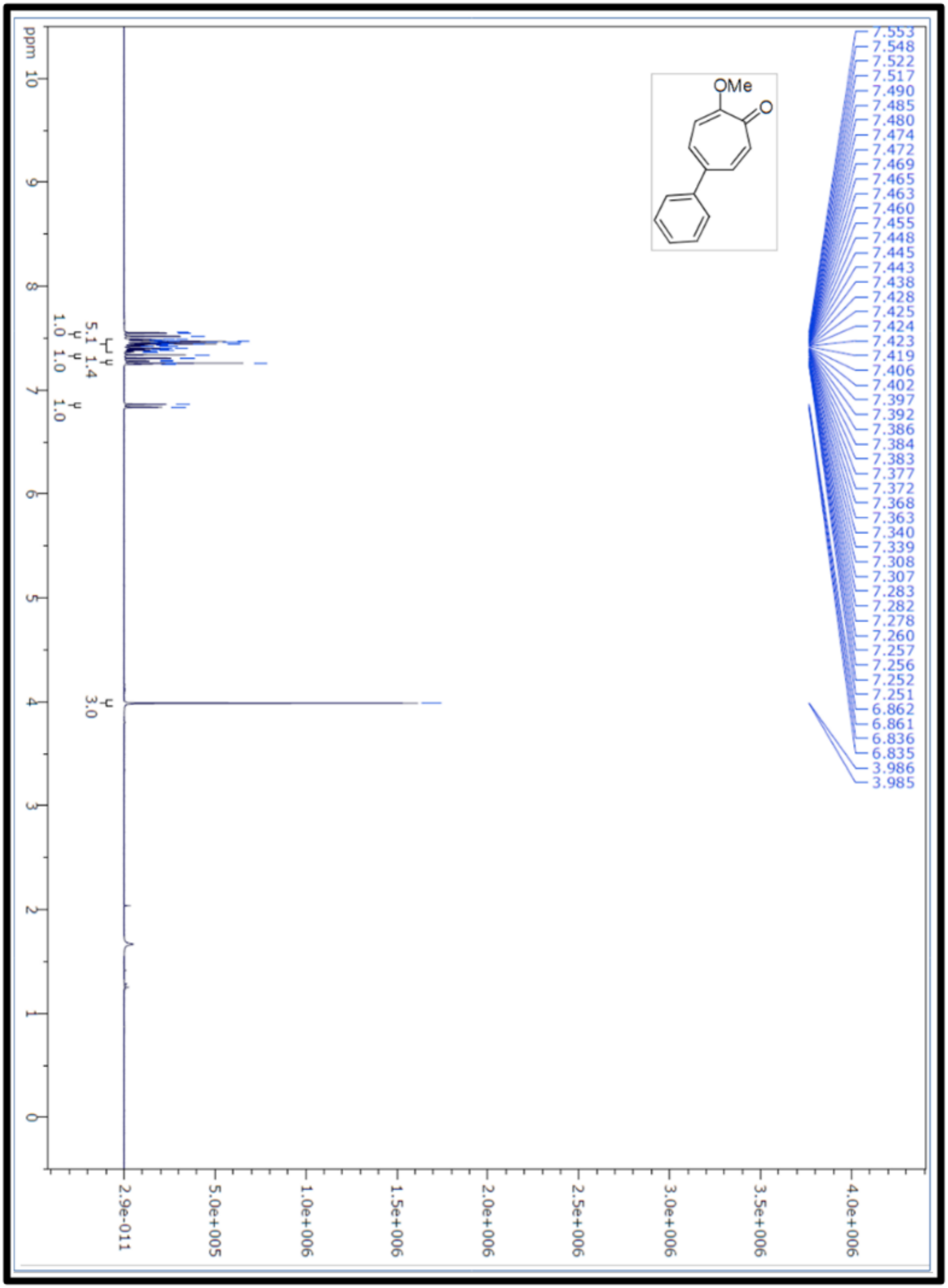

$^{13}C\{^{1}H\}$ **NMR (101 MHz, $CDCl_3$) of 8c**

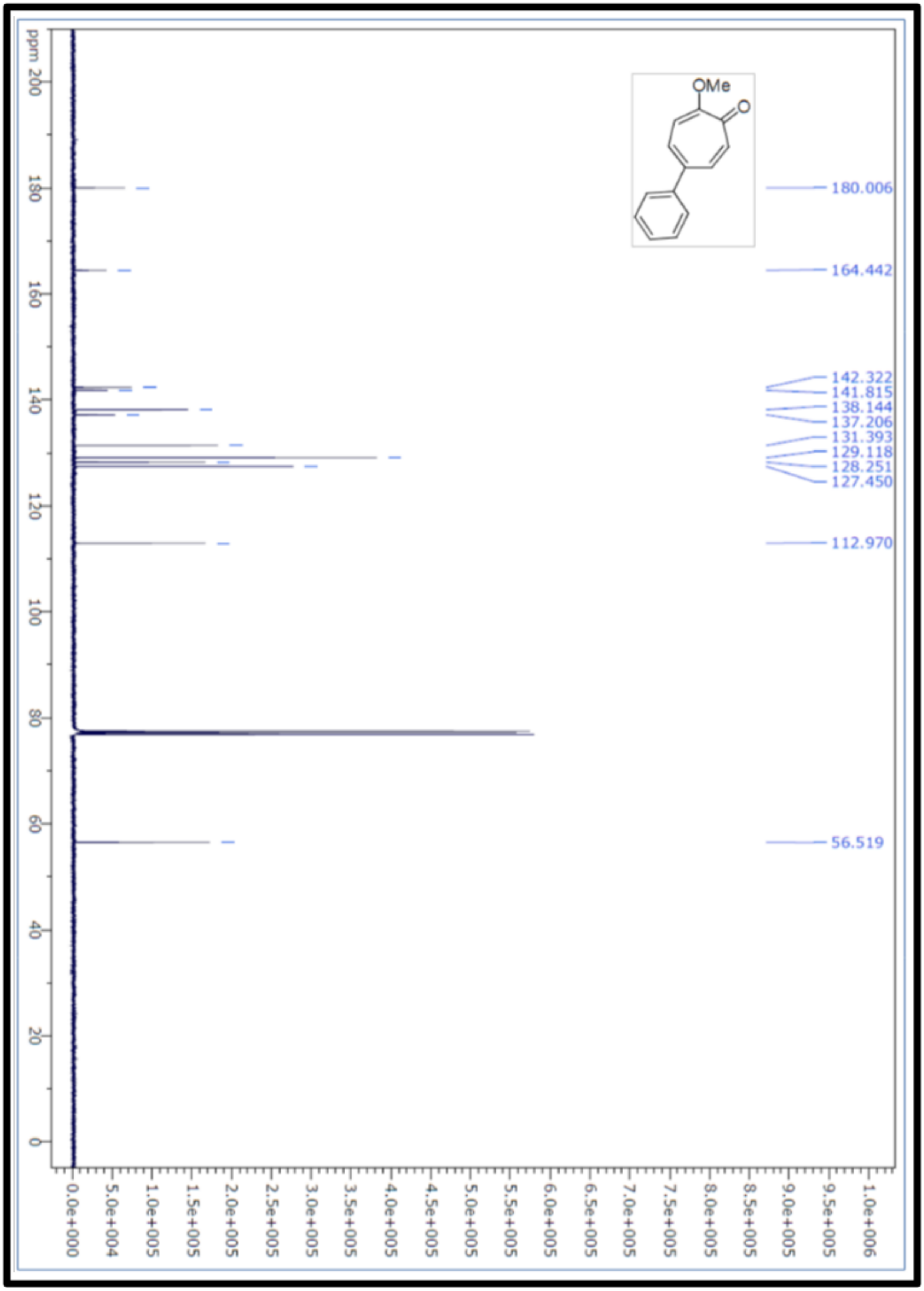

^{1}H NMR (400 MHz, CD_3CN) of 8c

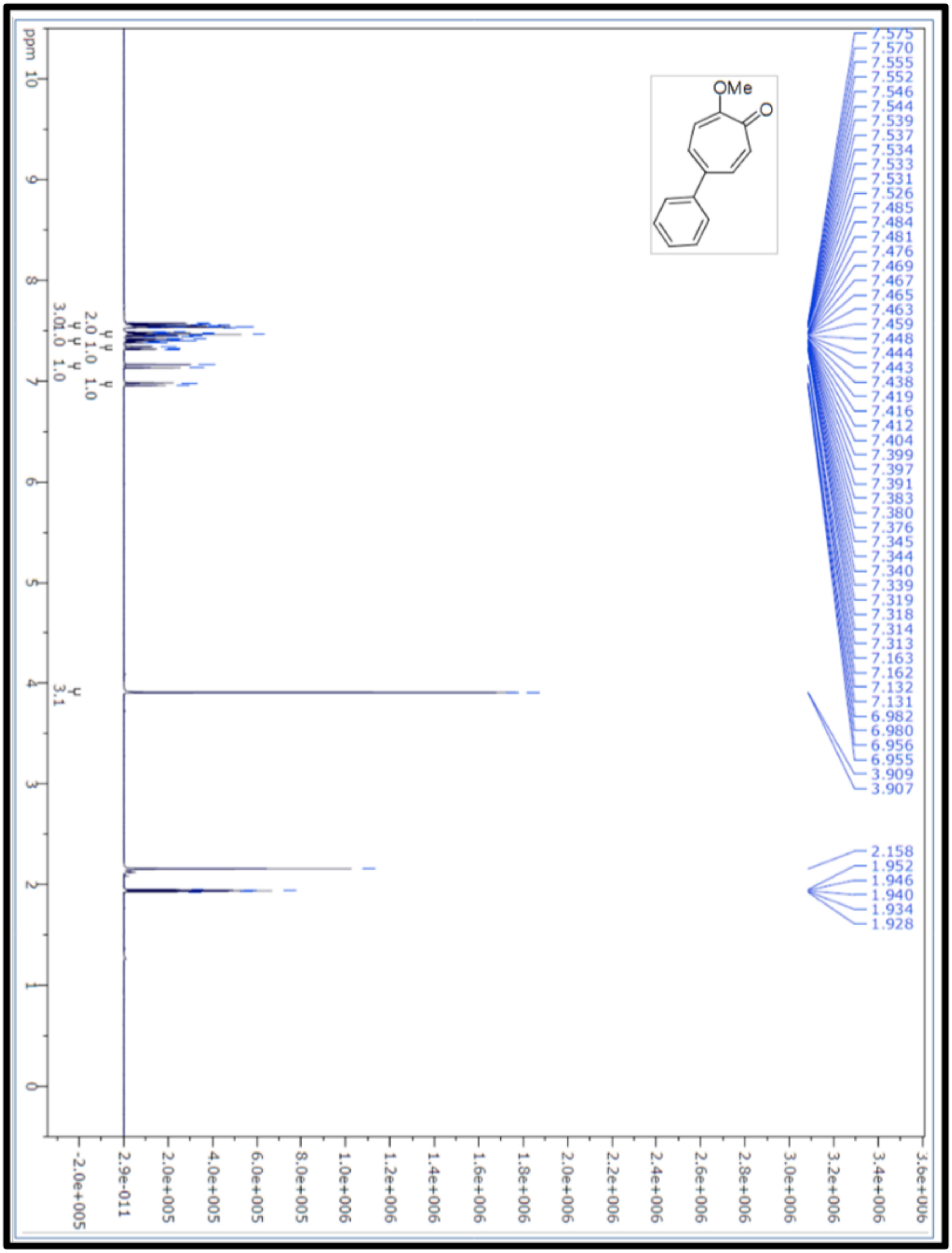

^{1}H NMR (400 MHz, CDCl3) of 8d

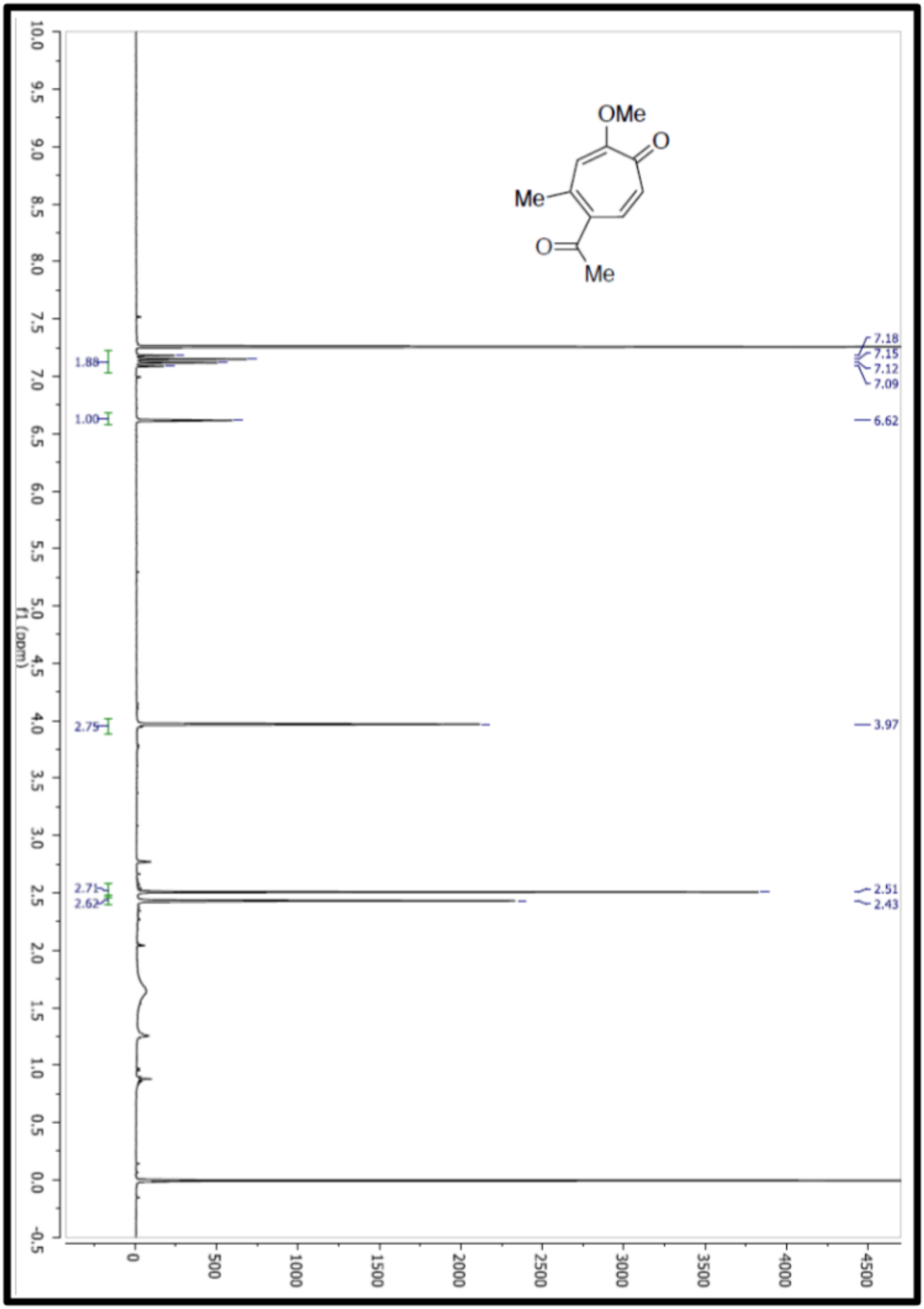

$^{13}C\{^{1}H\}$ NMR (101 MHz, $CDCl_3$) of 8d

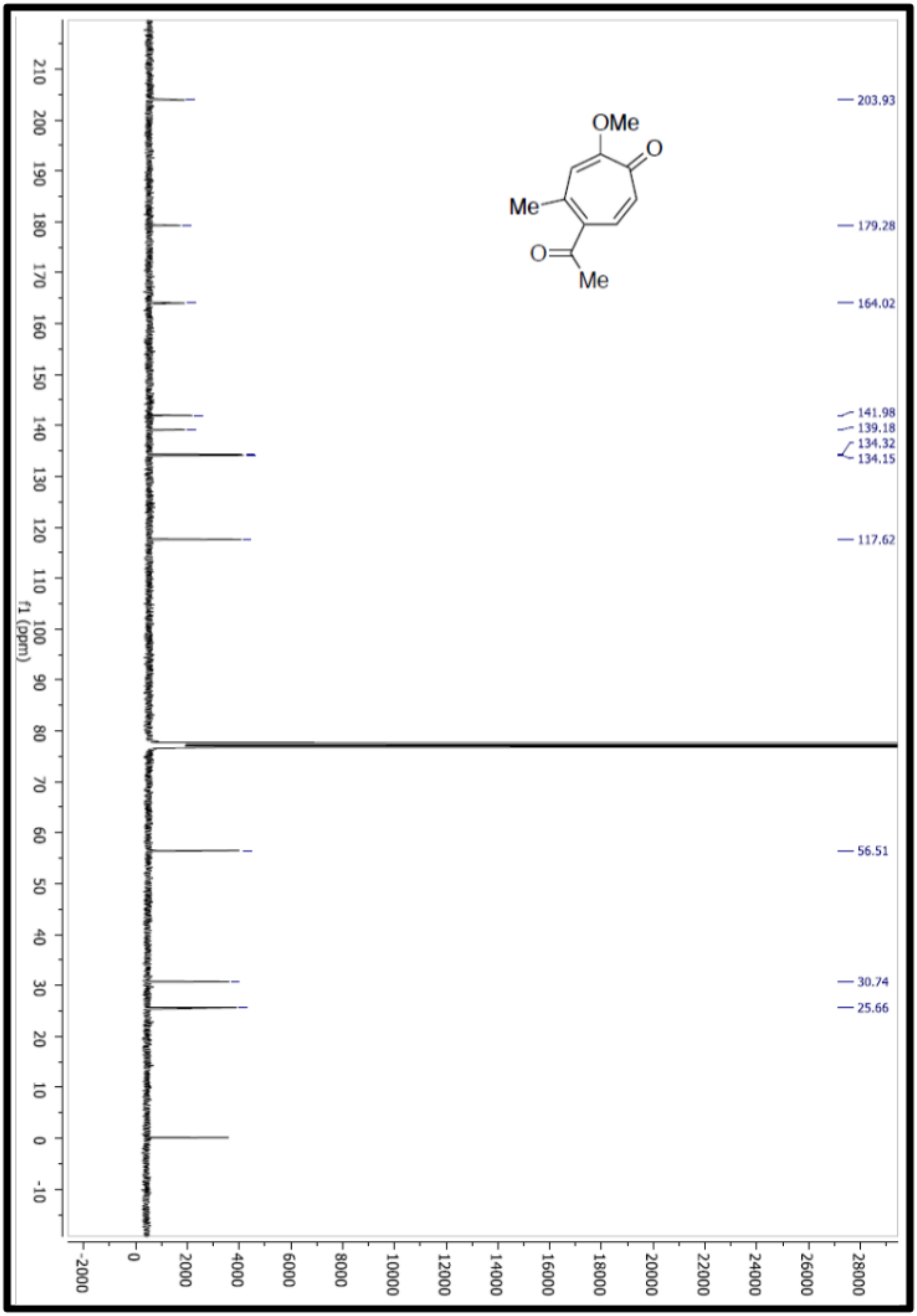

^{1}H NMR (400 MHz, $CDCl_3$) of 8e

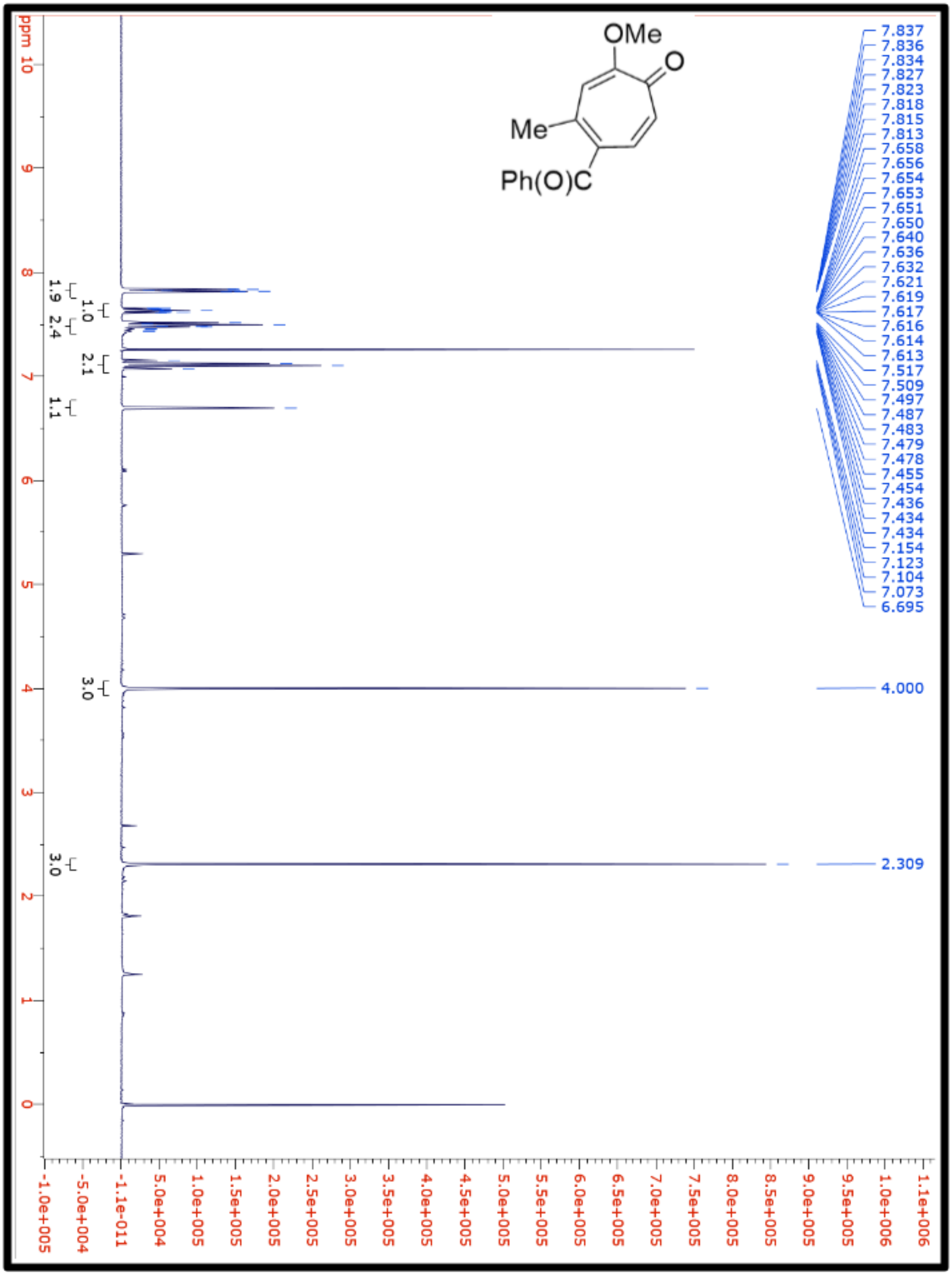

$^{13}C\{^{1}H\}$ NMR (101 MHz, $CDCl_3$) of 8e

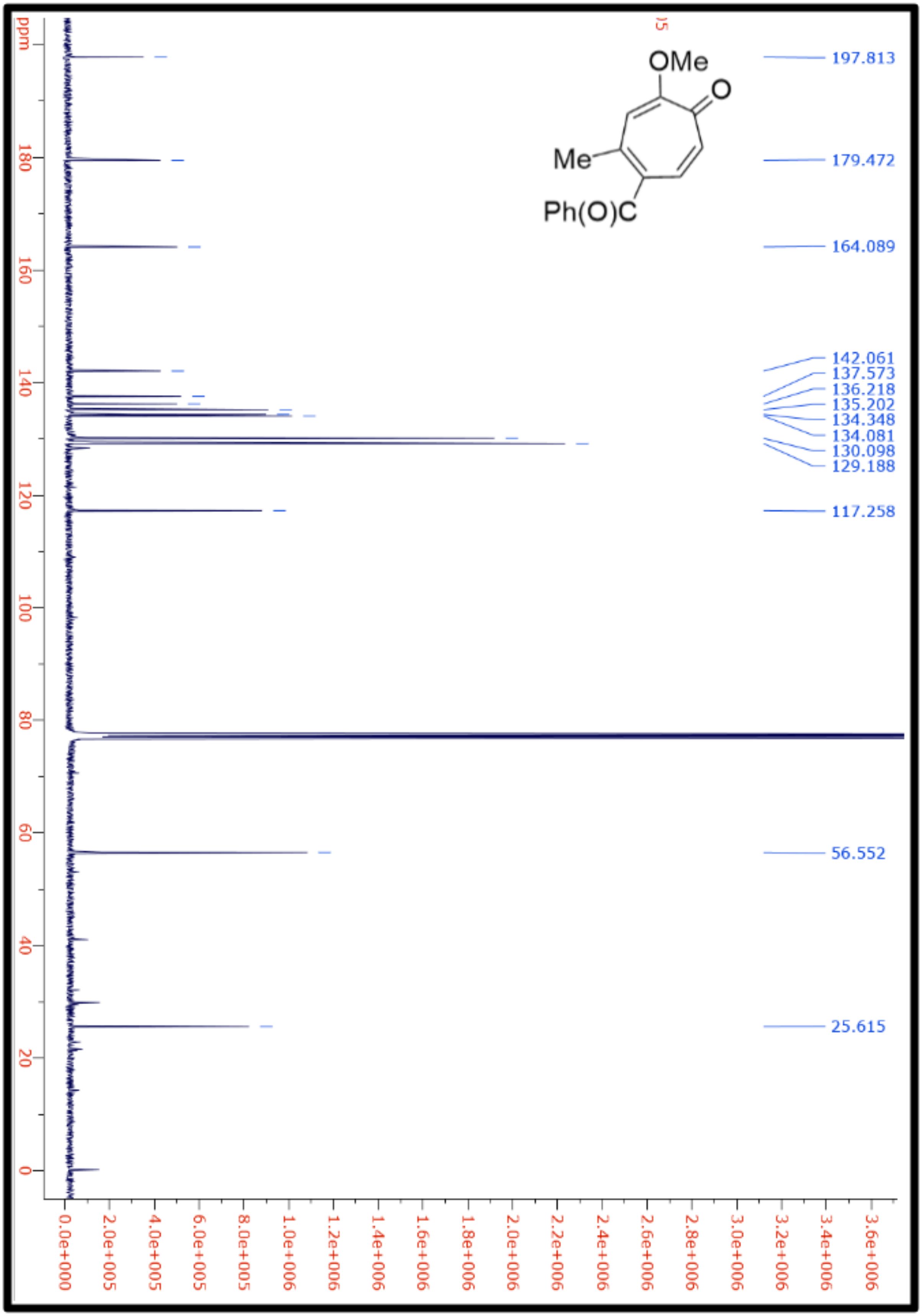

^{1}H NMR (400 MHz, CDCl3) of 8f

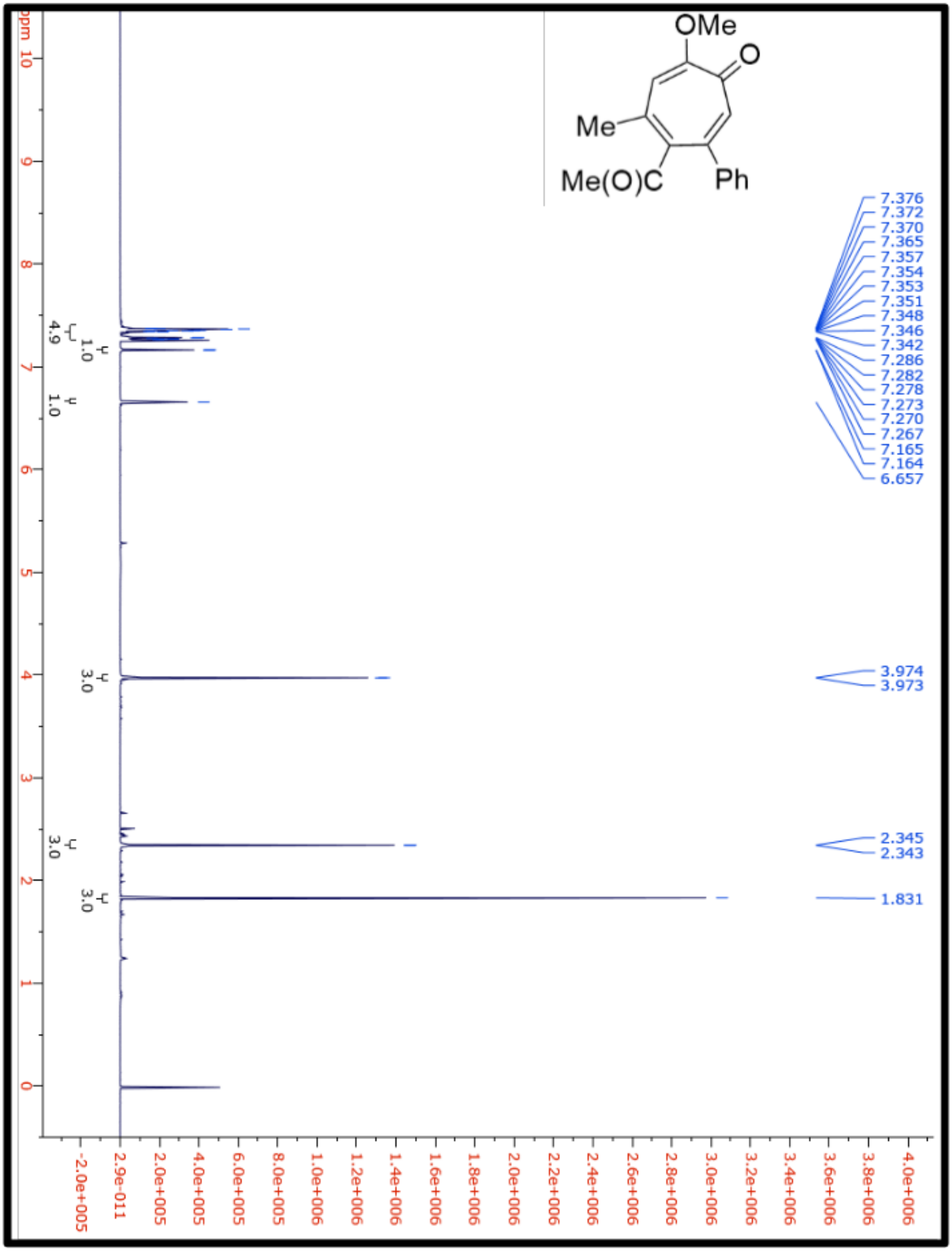

$^{13}C\{^{1}H\}$ NMR (101 MHz, $CDCl_3$) of 8f

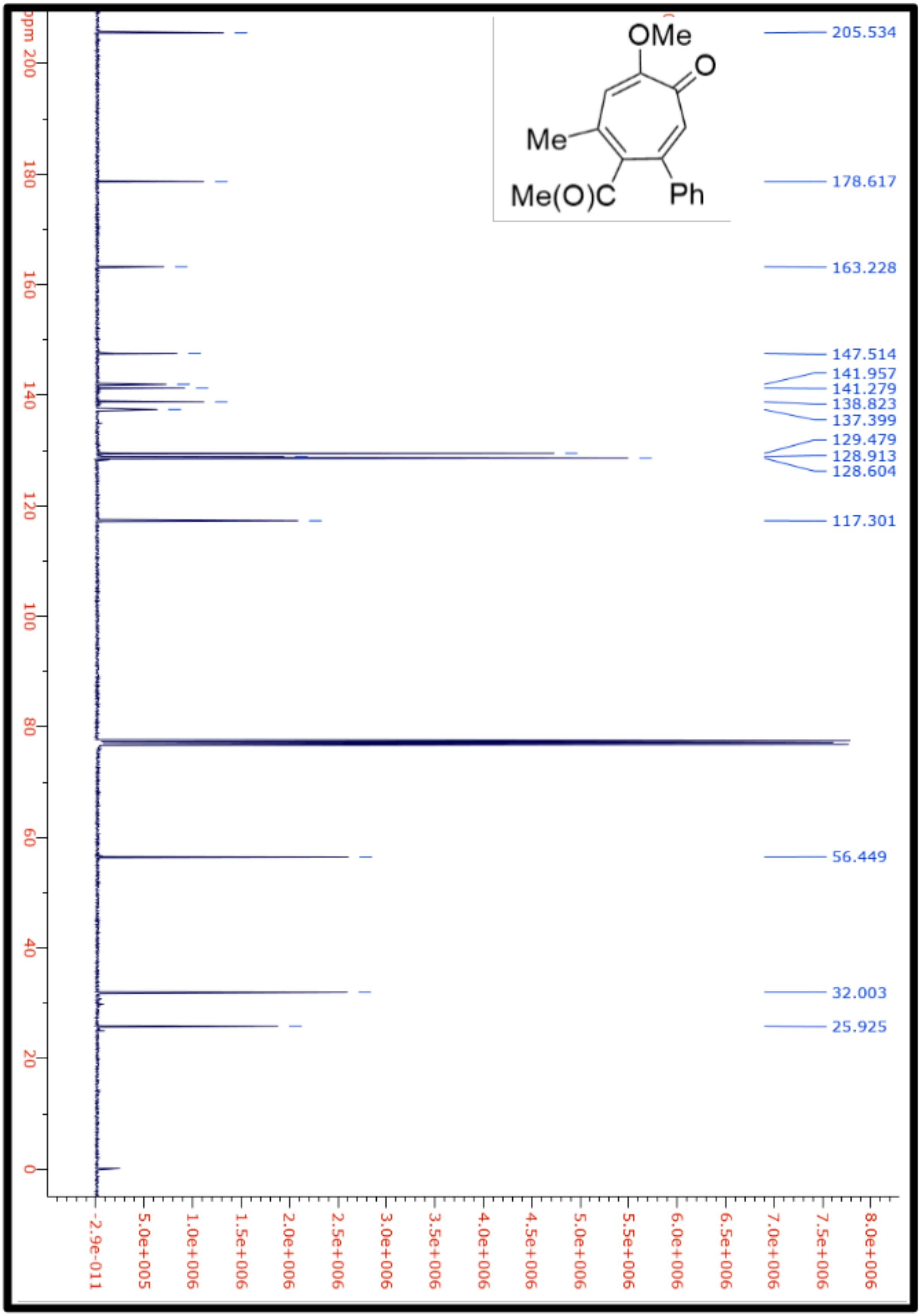

^{1}H NMR (400 MHz, $CDCl_3$) of 8g

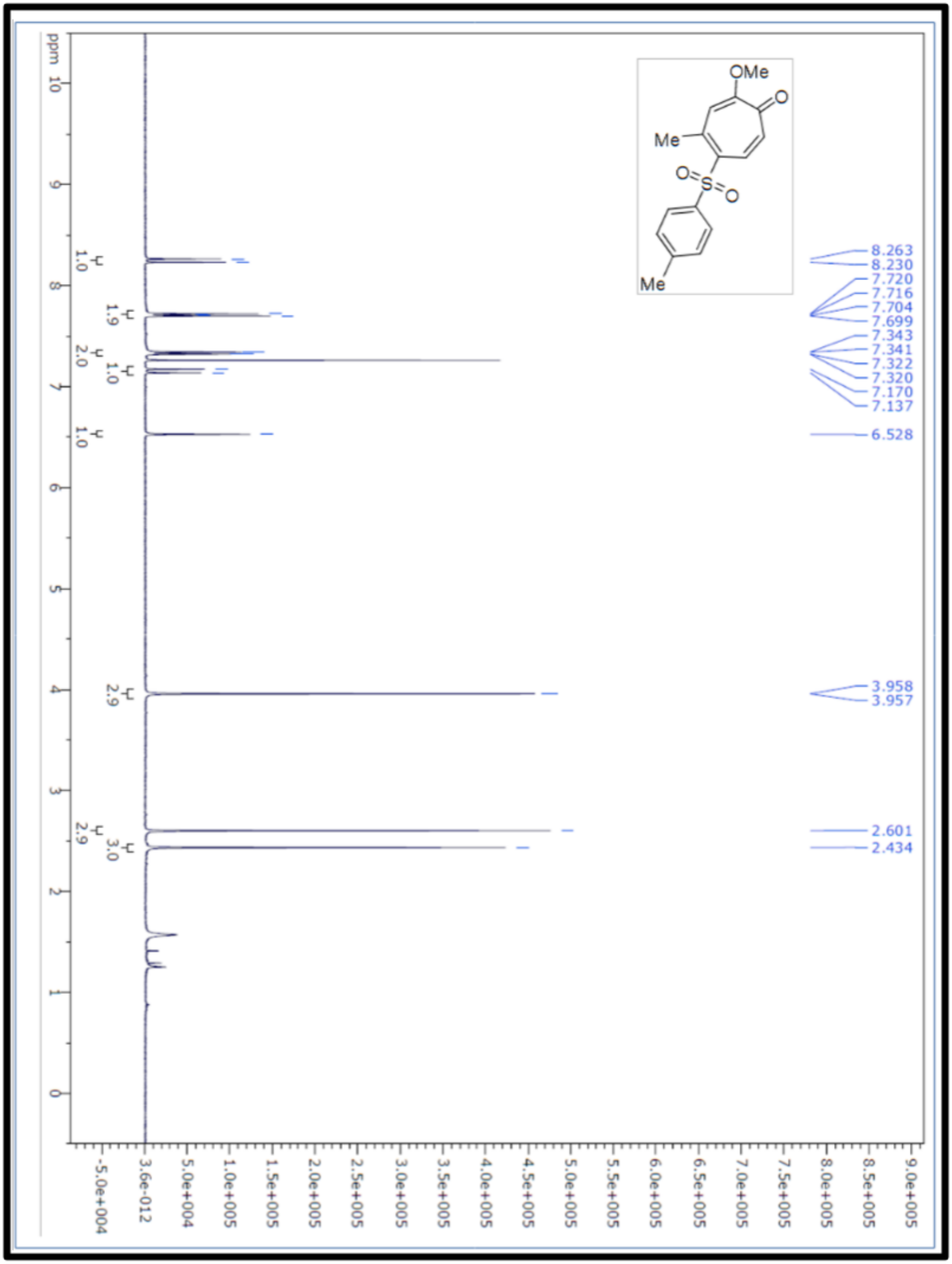

$^{13}C\{^1H\}$ NMR (101 MHz, $CDCl_3$) of 8g

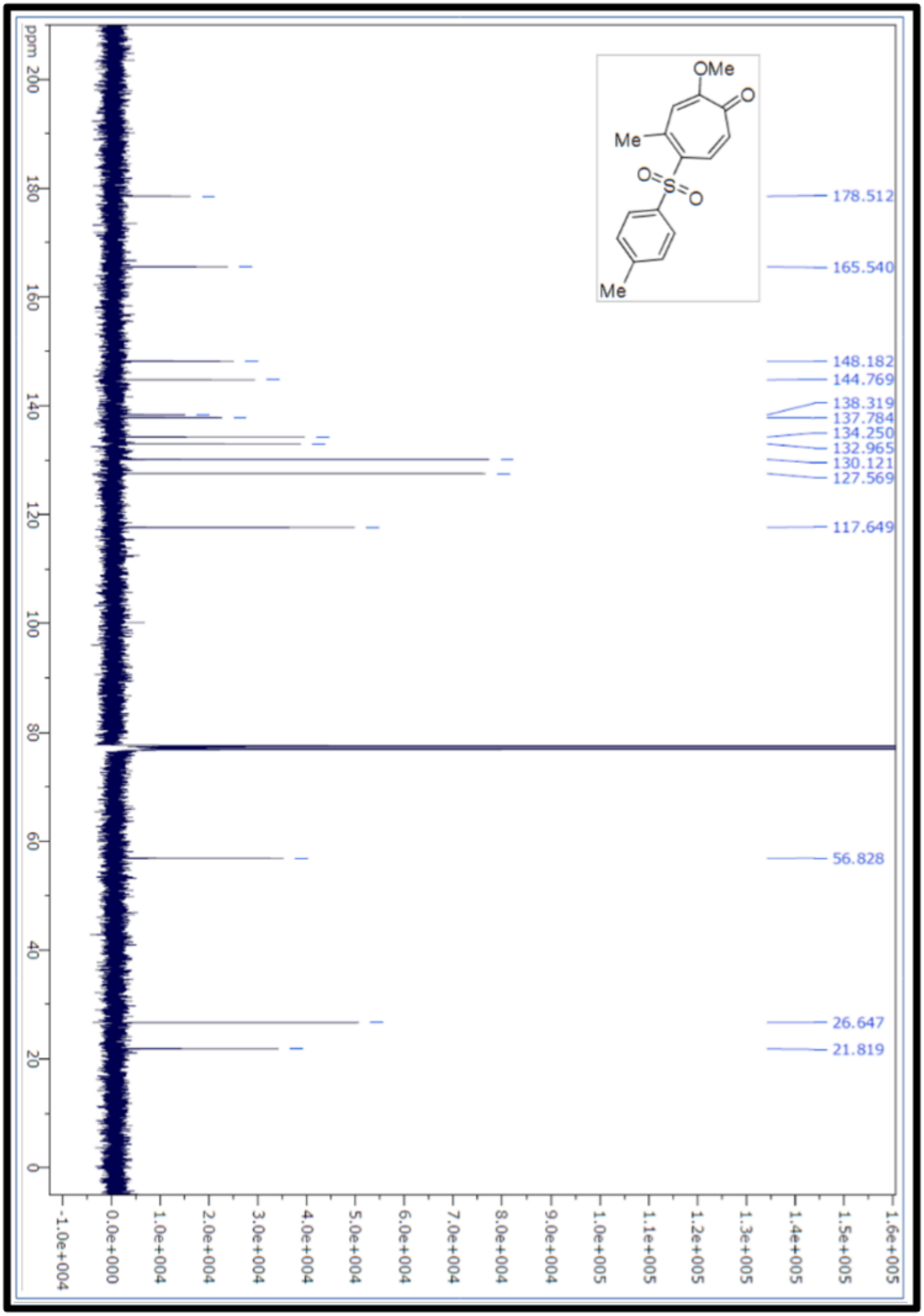

^{1}H NMR (400 MHz, $CDCl_3$) of 8h

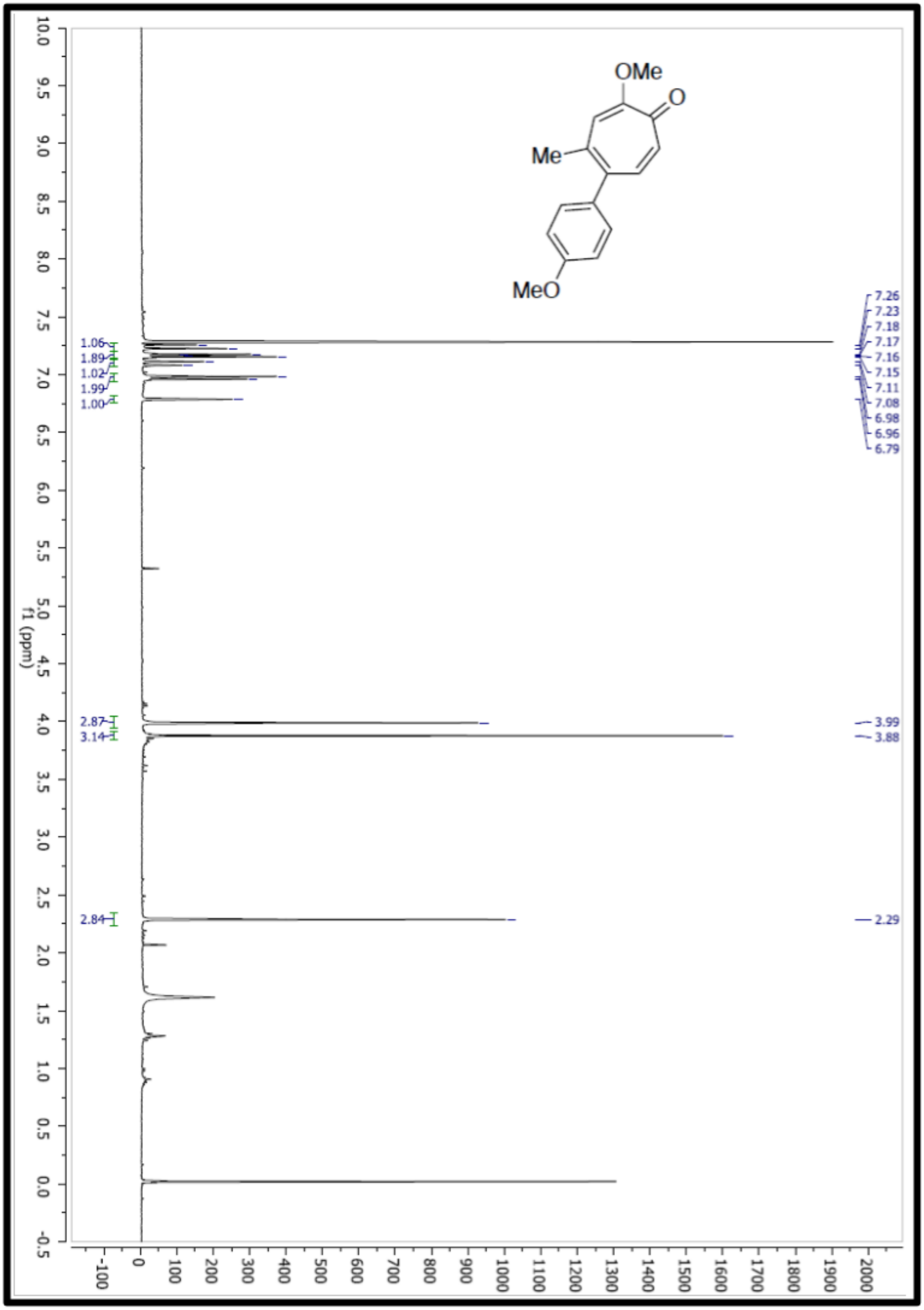

$^{13}C\{^{1}H\}$ NMR (101 MHz, $CDCl_3$) of 8h

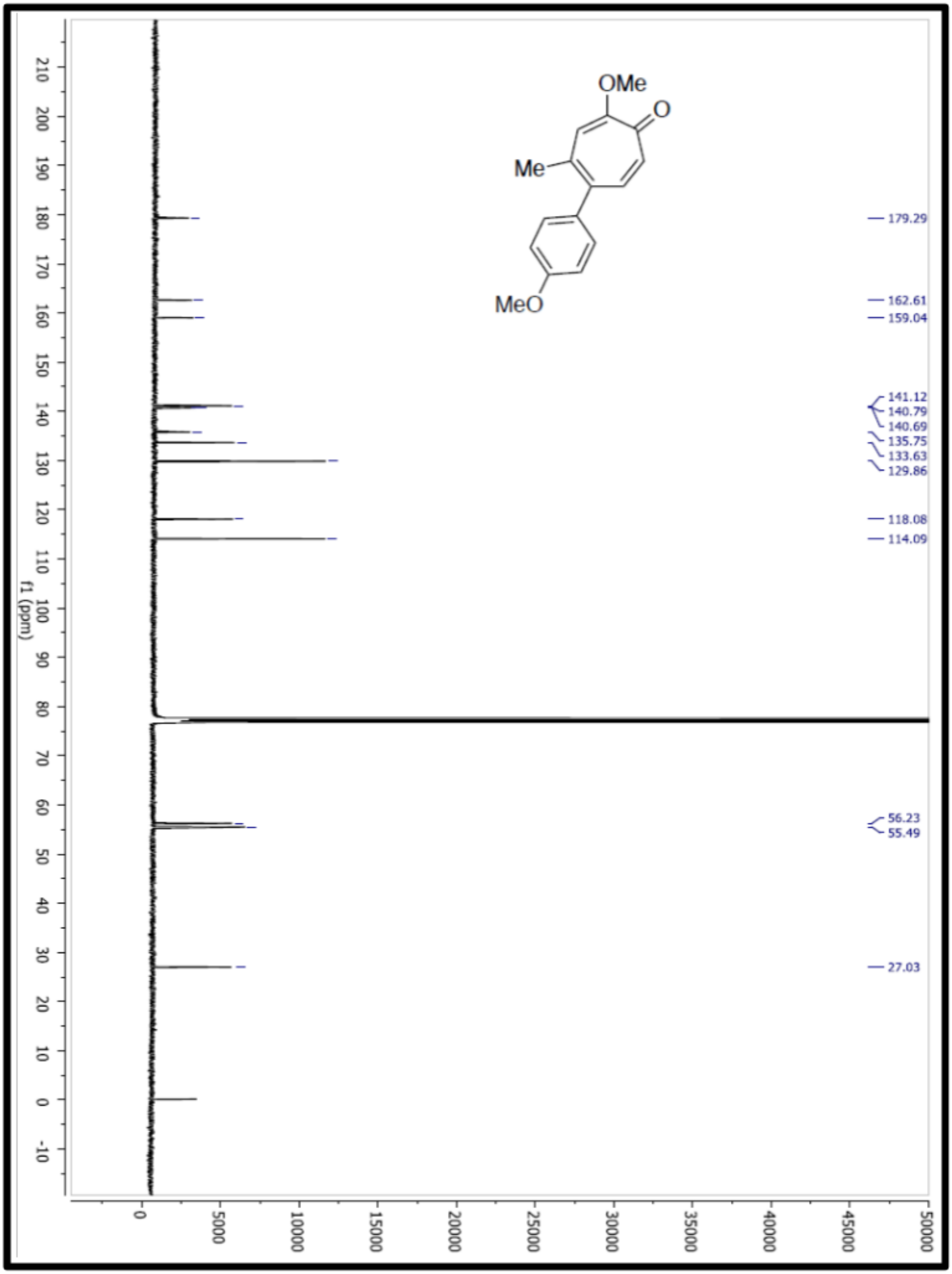

^{1}H NMR (400 MHz, CDCl3) of 8i

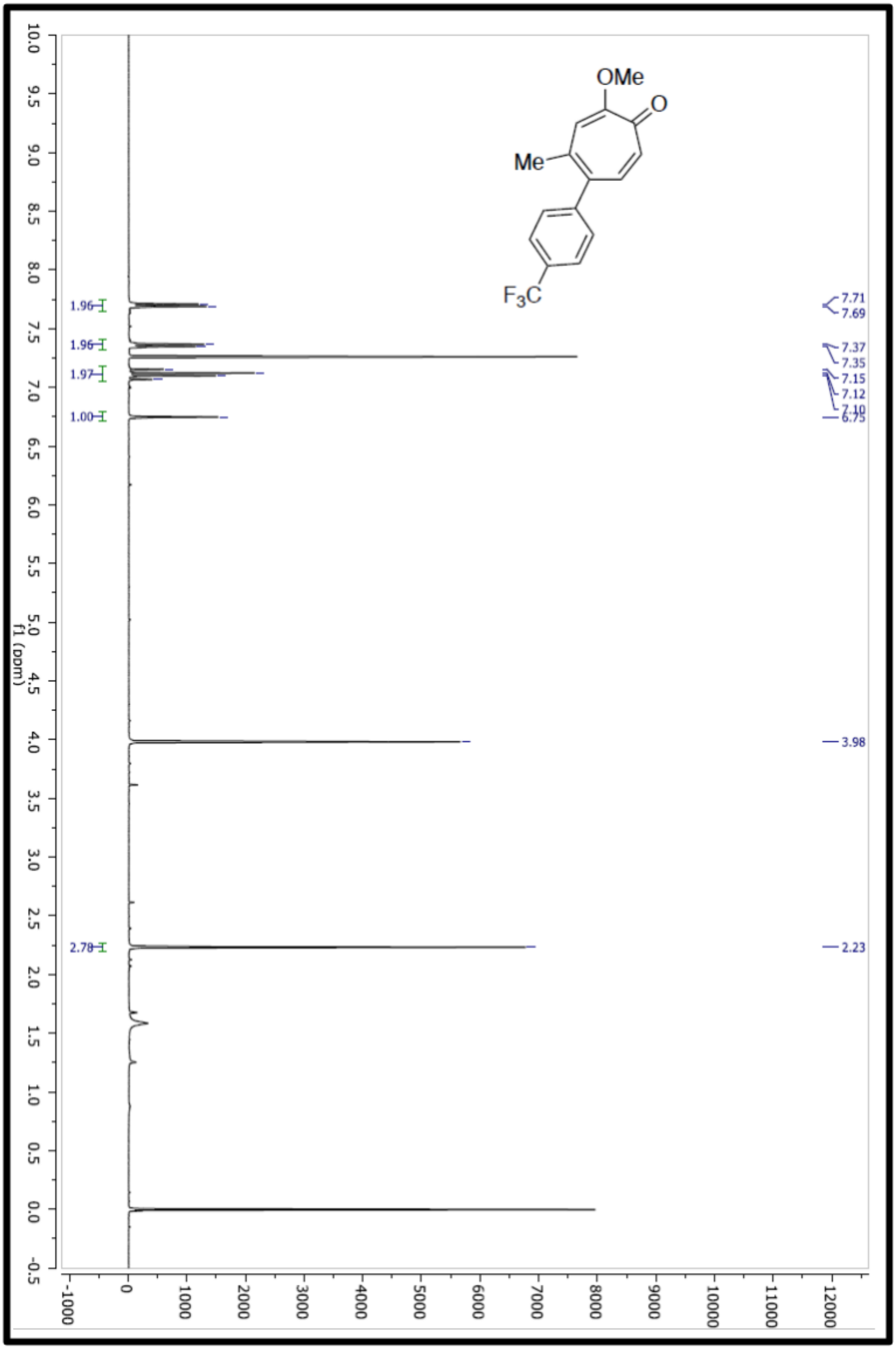

$^{13}C\{^{1}H\}$ NMR (101 MHz, $CDCl_3$) of 8i

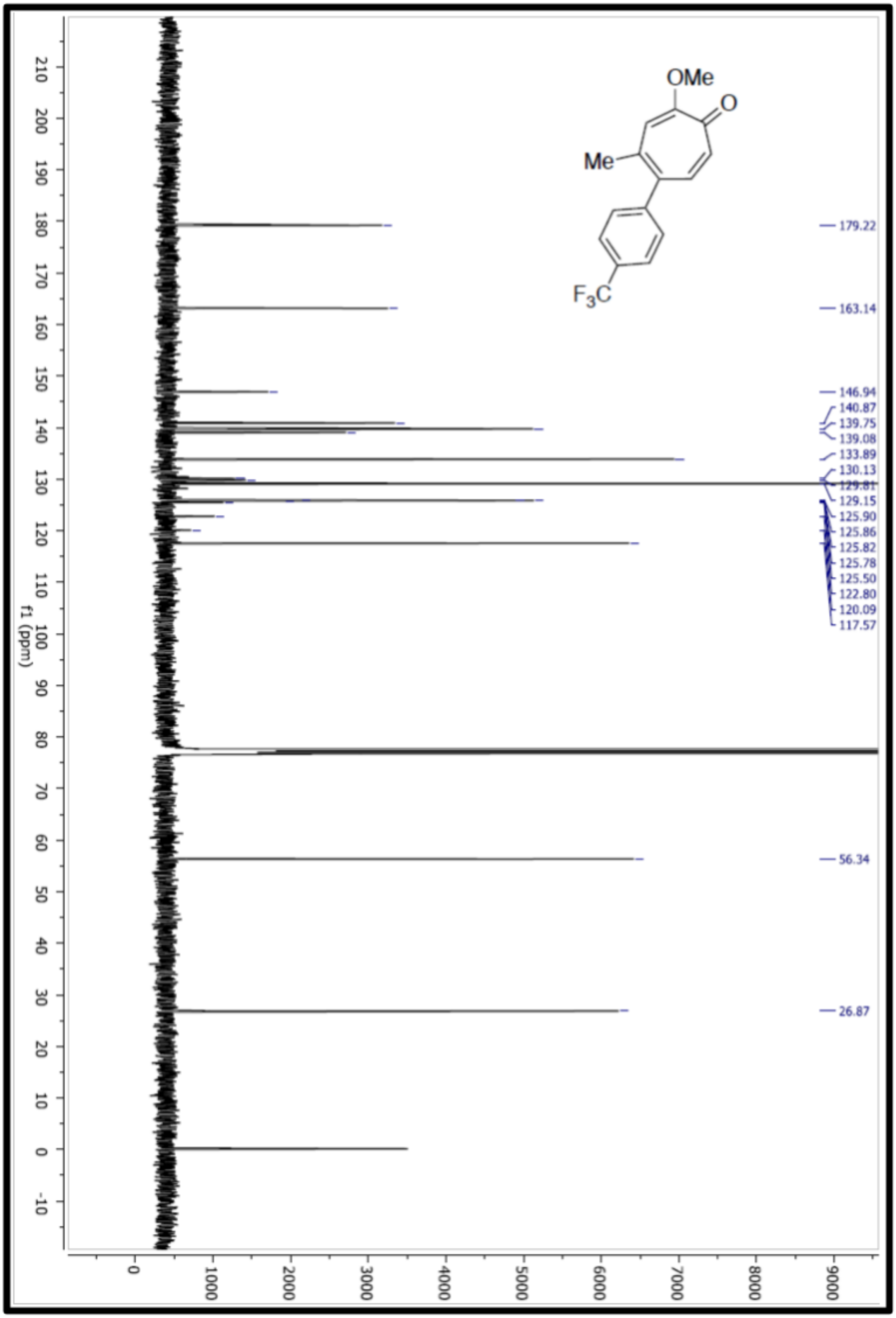

^{1}H NMR (400 MHz, $CDCl_3$) of 8k

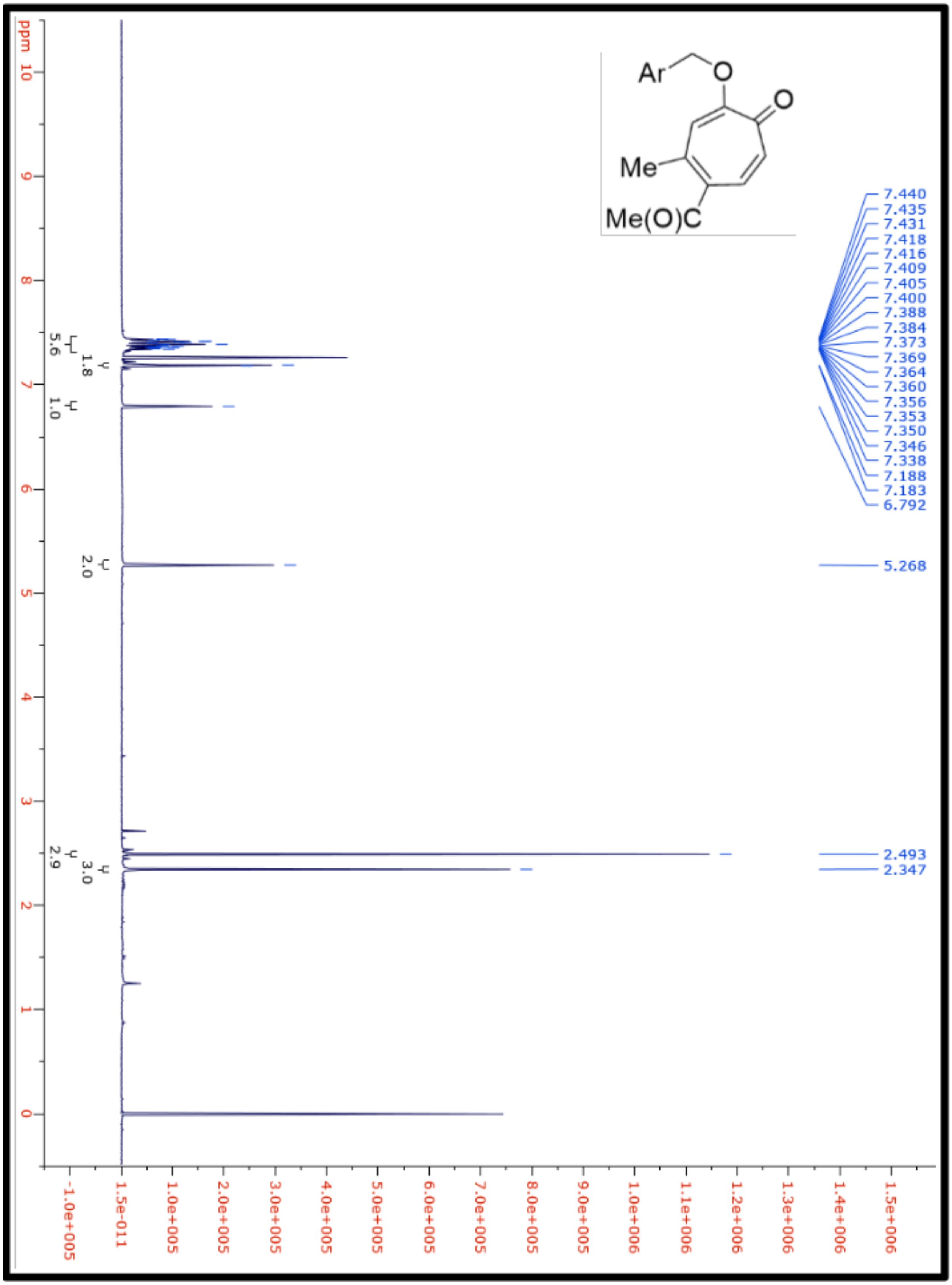

^{13}C{^{1}H} NMR (101 MHz, $CDCl_3$) of 8k

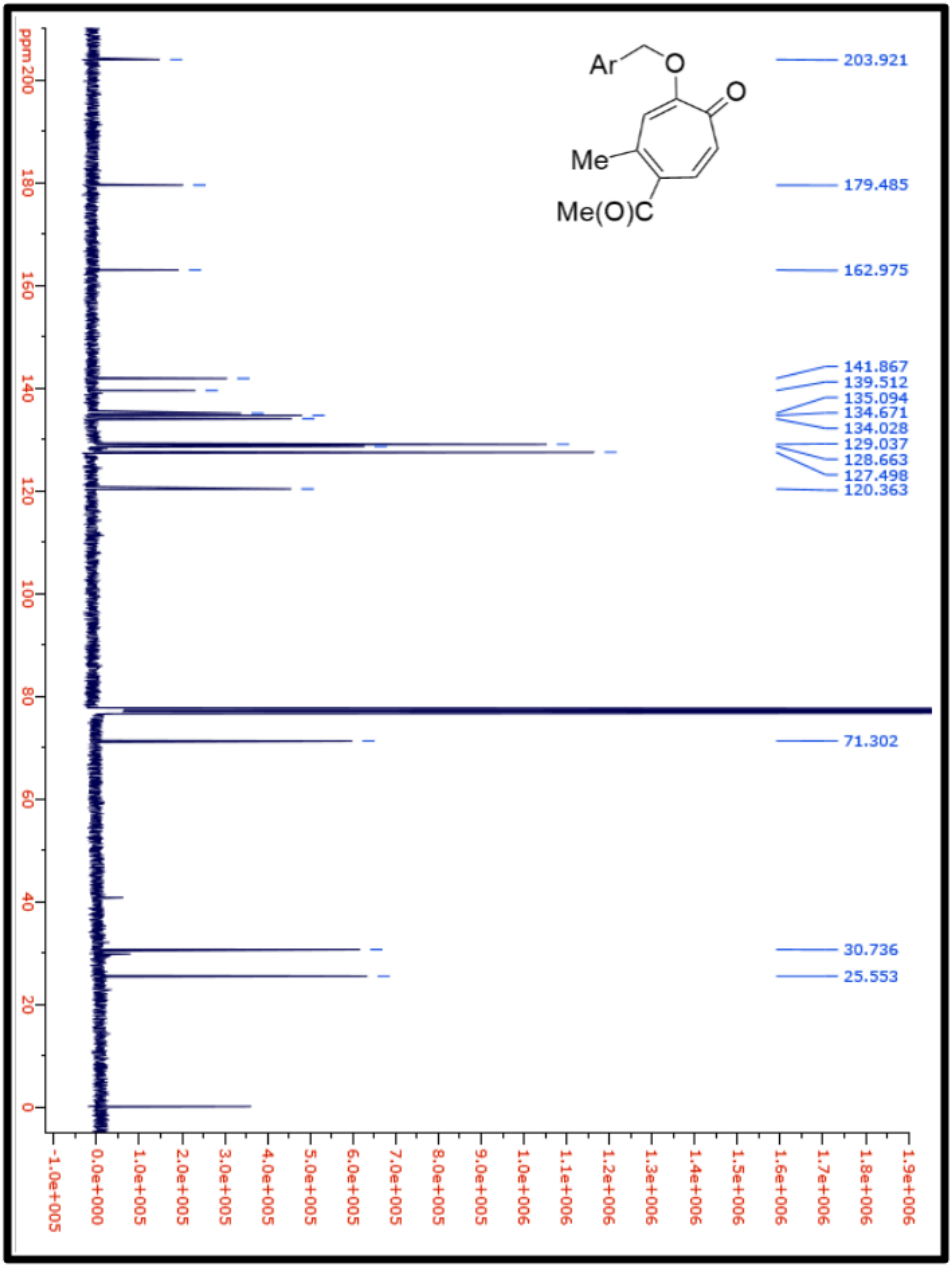

^{1}H NMR (400 MHz, $CDCl_3$) of 8l

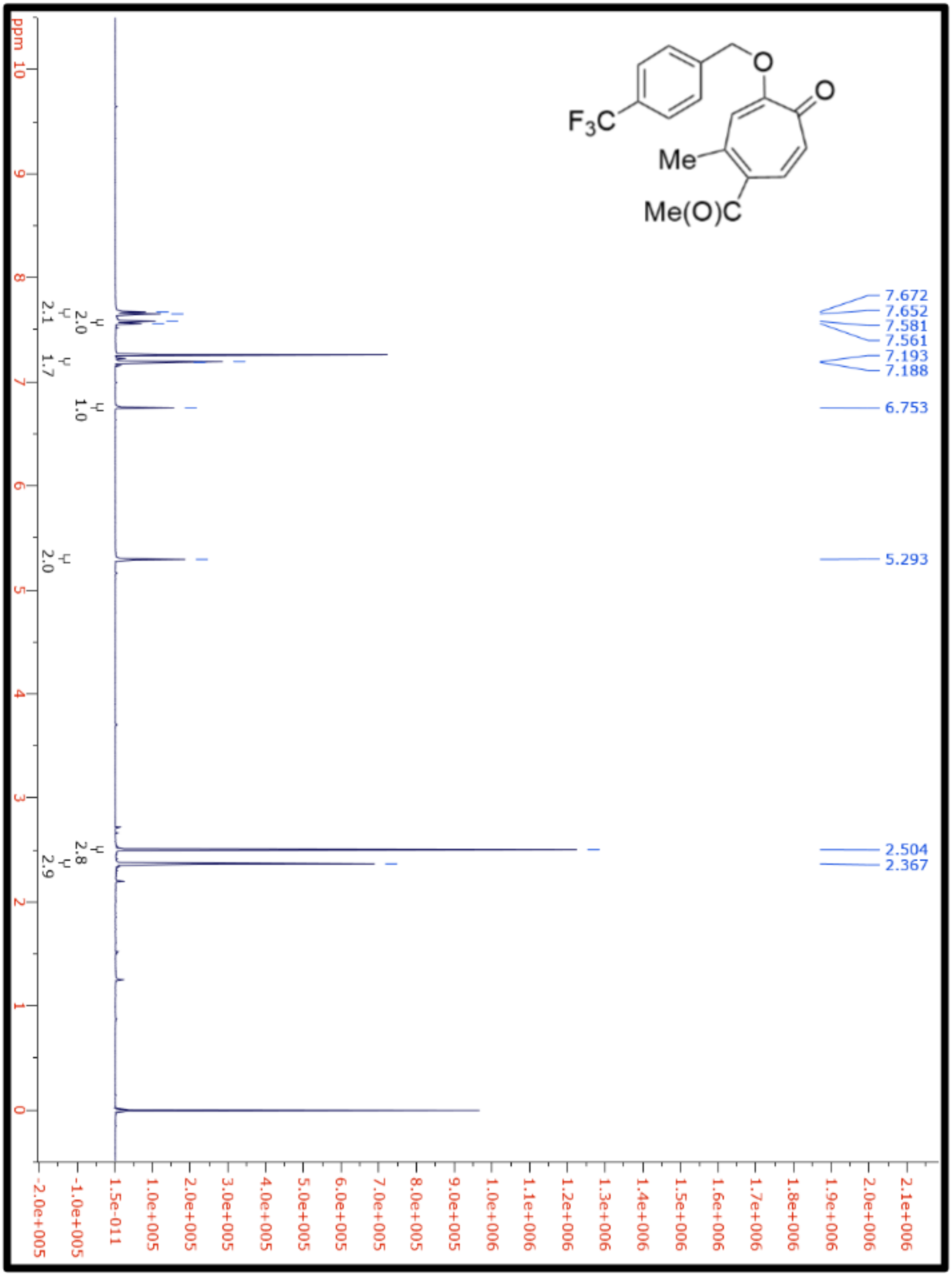

$^{13}C\{^{1}H\}$ NMR (101 MHz, $CDCl_3$) of 8l

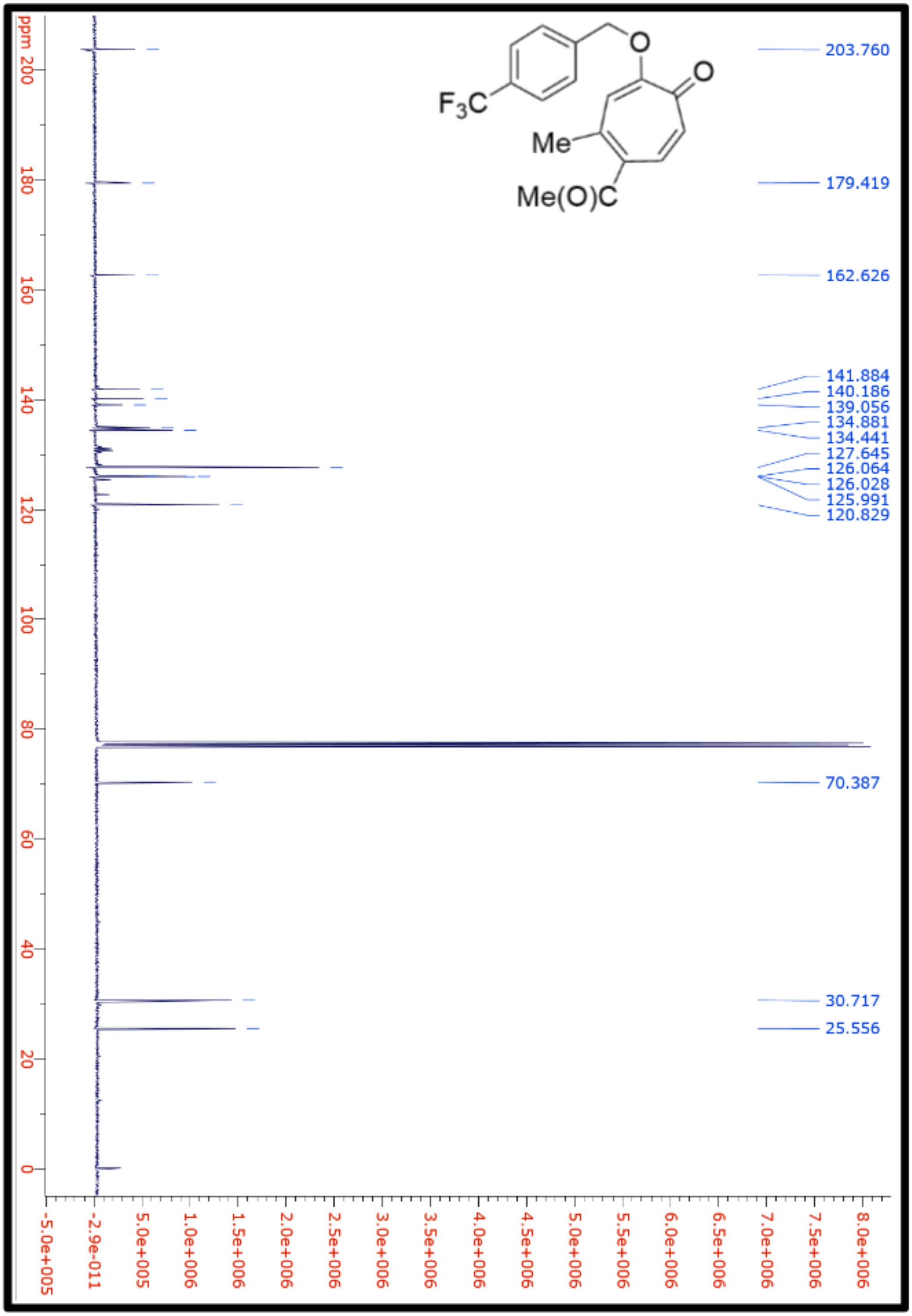

^{1}H NMR (400 MHz, CDCl3) of 25

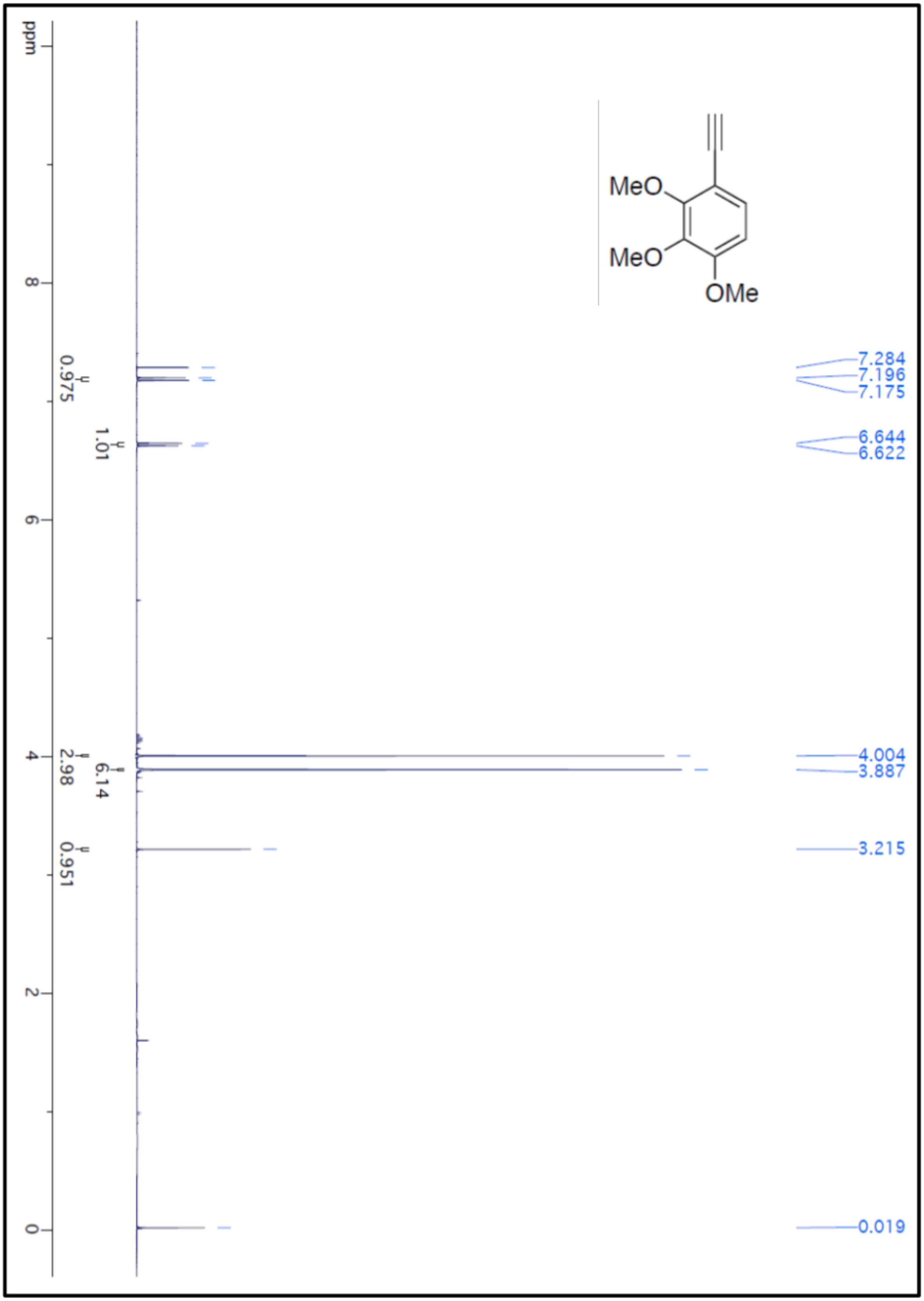

^{1}H NMR (400 MHz, $CDCl_3$) of S1

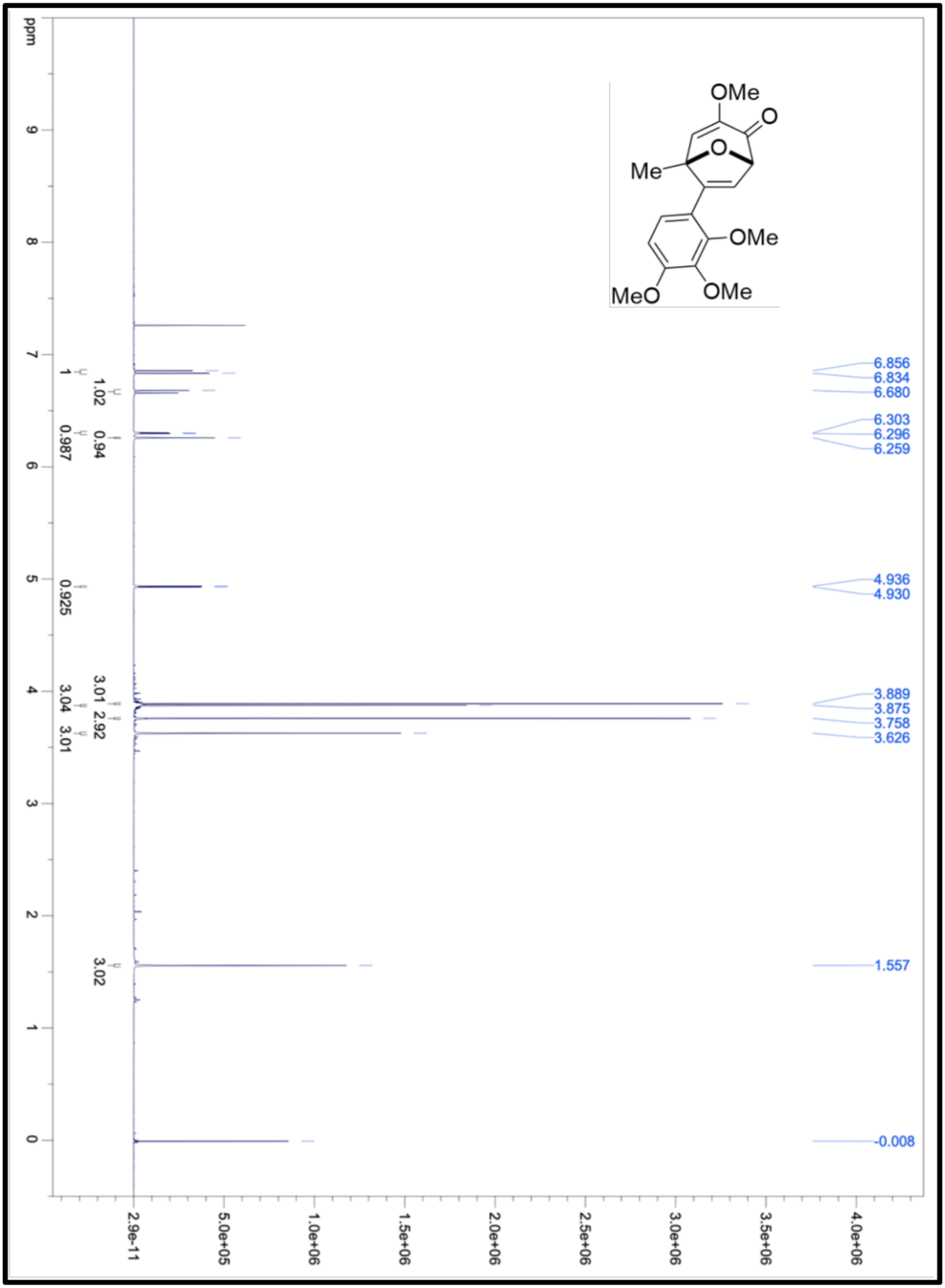

^{13}C{^{1}H} NMR (101 MHz, CDCl₃) of S1

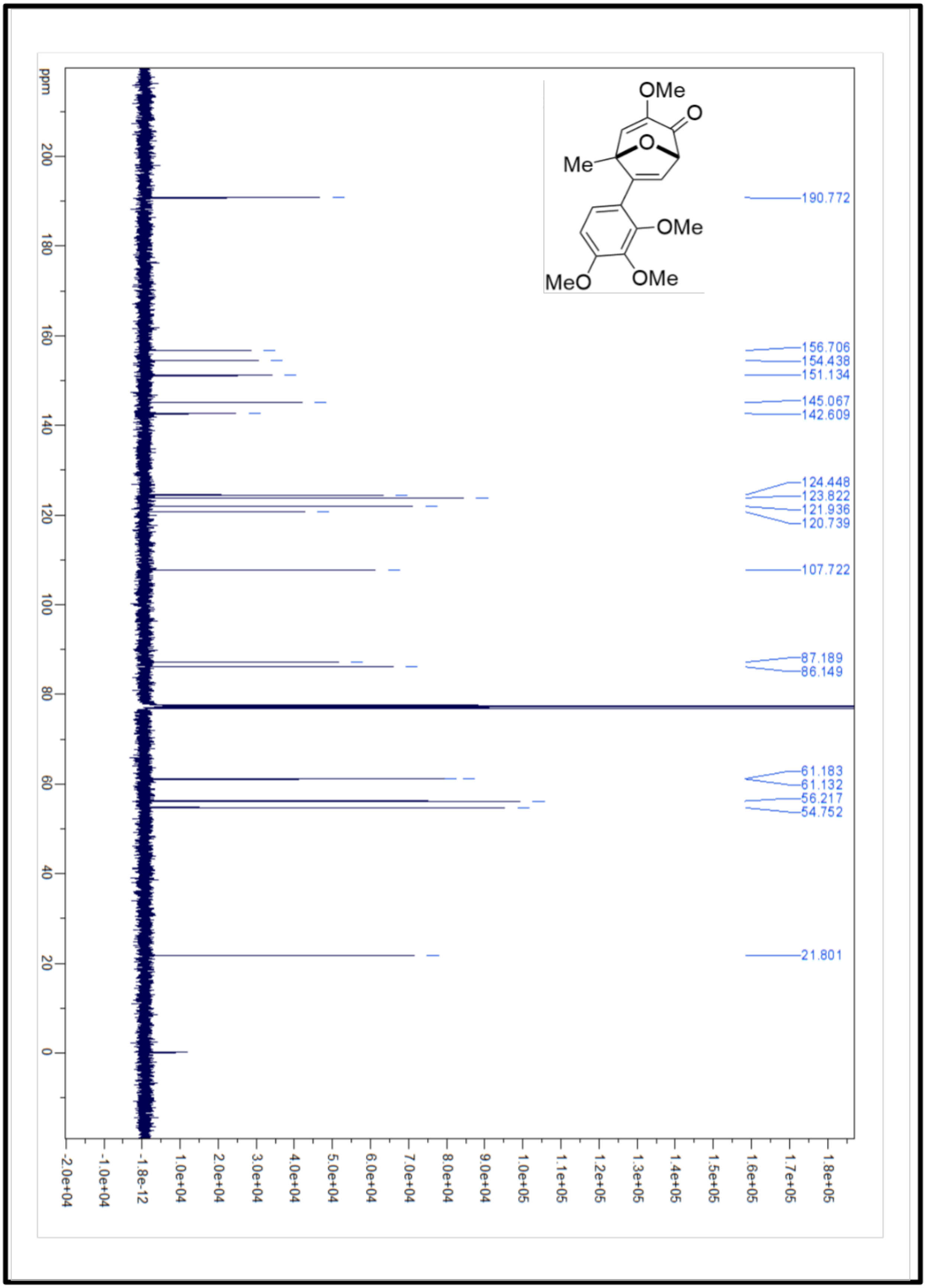

^{1}H NMR (400 MHz, $CDCl_3$) of 26

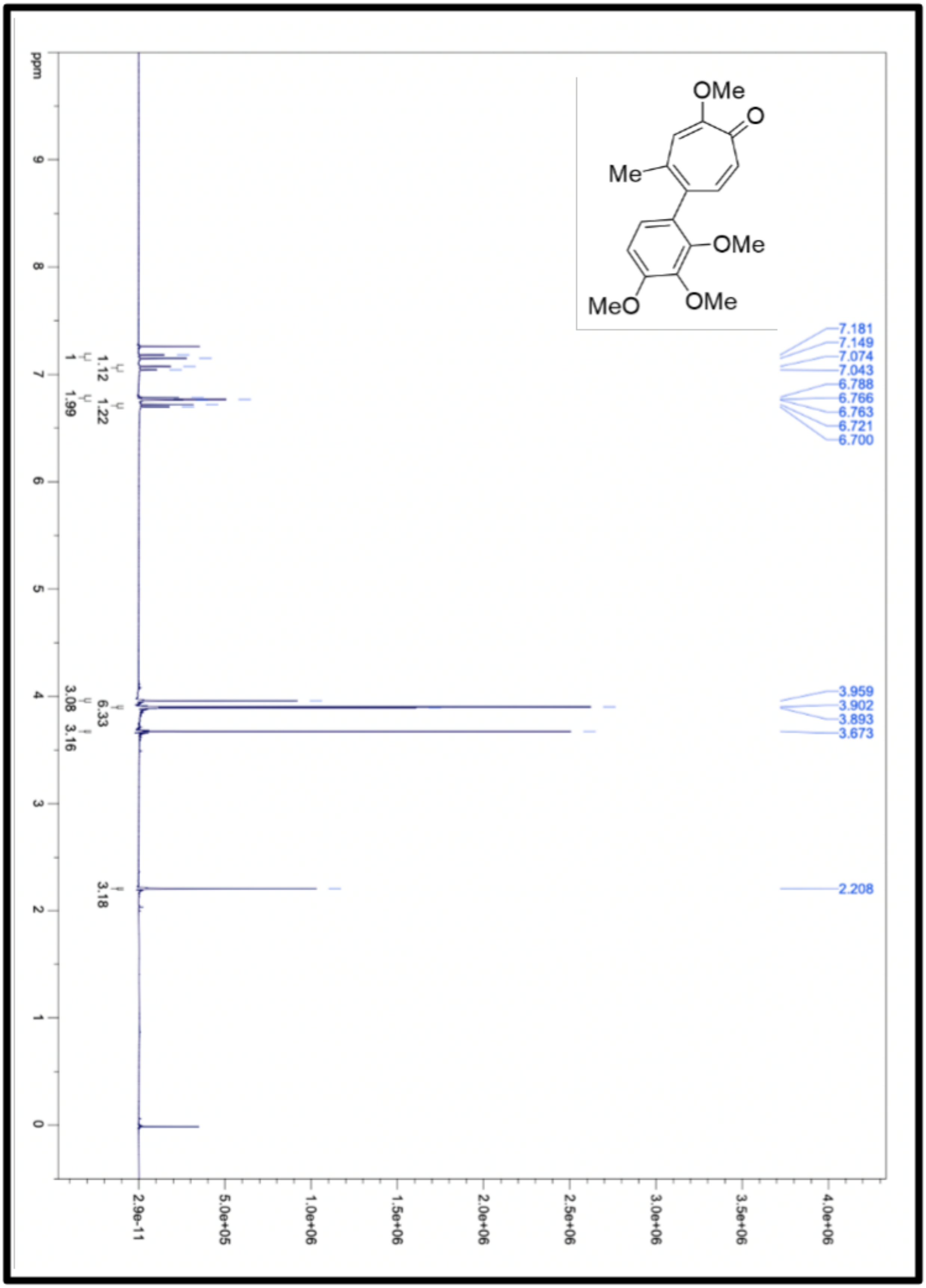

$^{13}C\{^{1}H\}$ NMR (101 MHz, $CDCl_3$) of 26

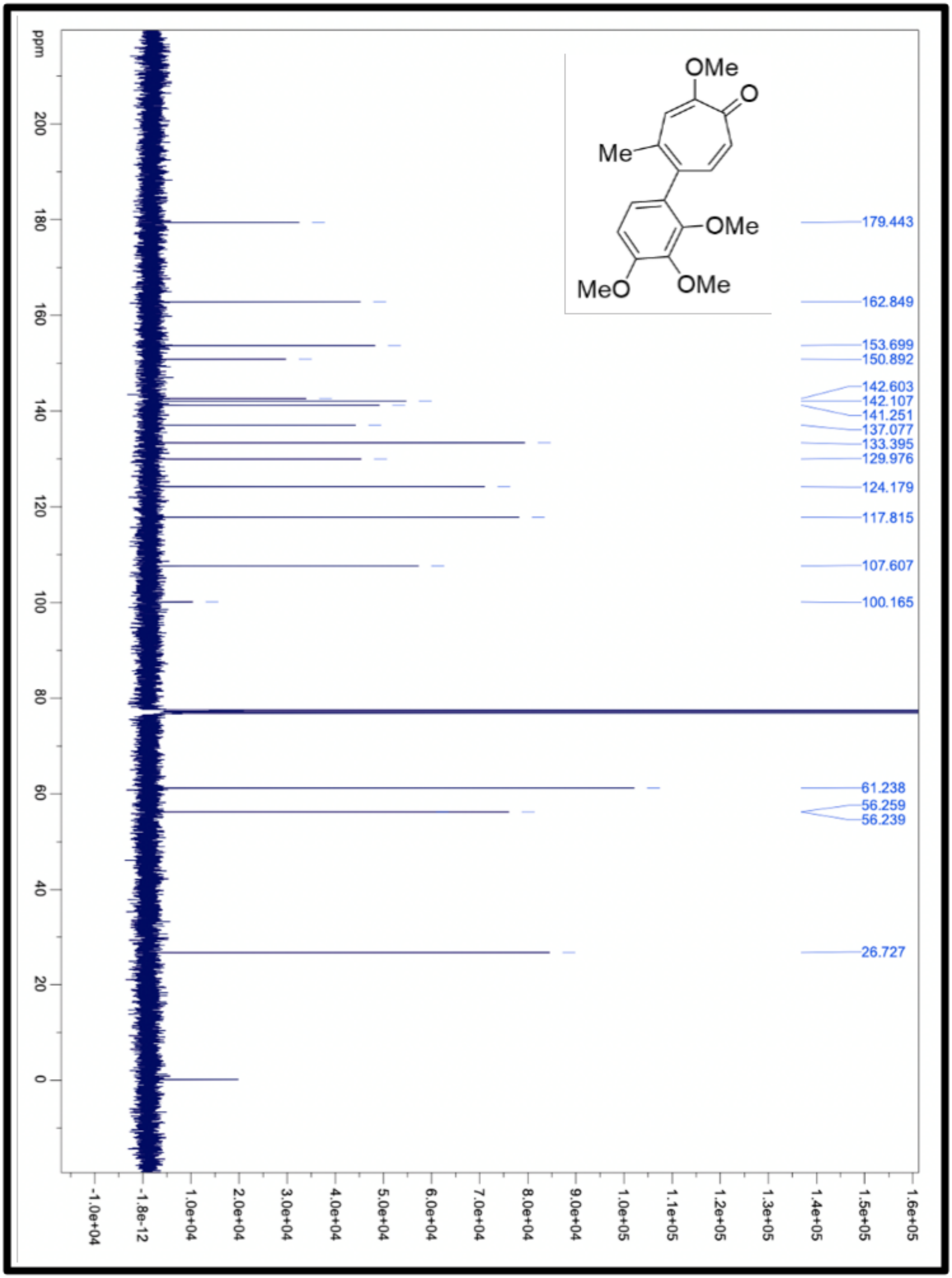

^{1}H NMR (400 MHz, $CDCl_3$) of S2

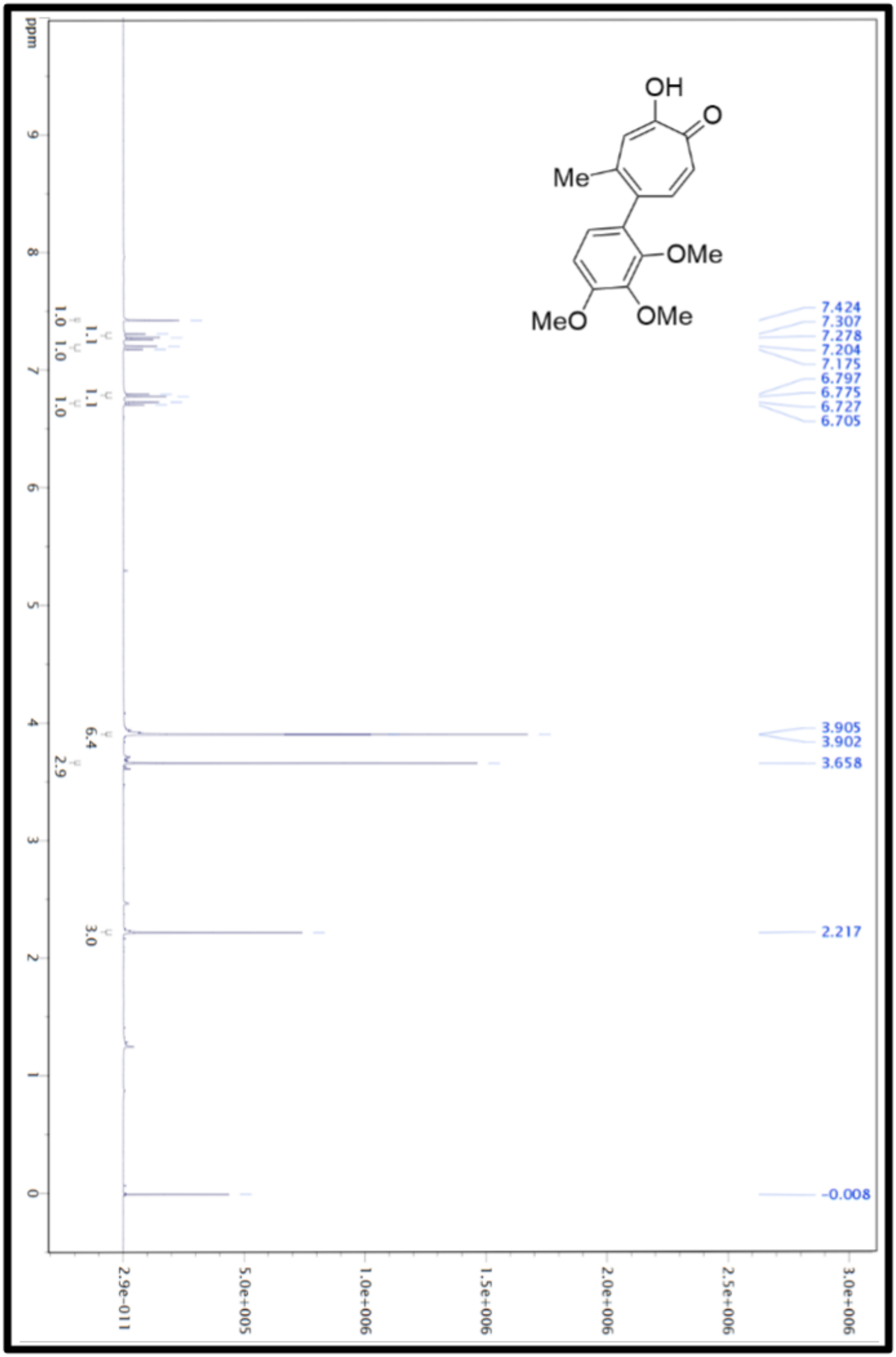

$^{13}C\{^{1}H\}$ NMR (101 MHz, $CDCl_3$) of S2

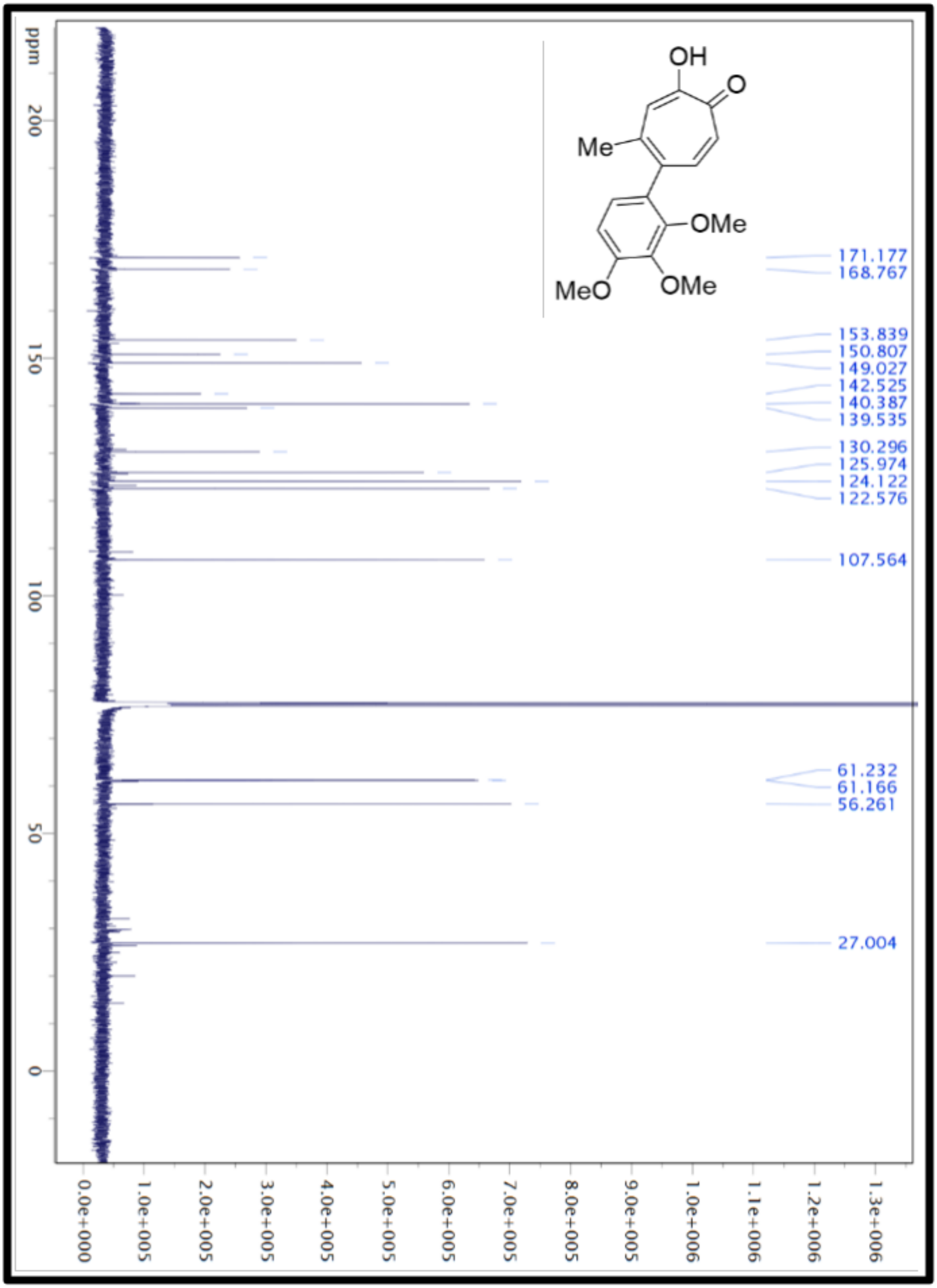

^{1}H NMR (400 MHz, $CDCl_3$) of 27

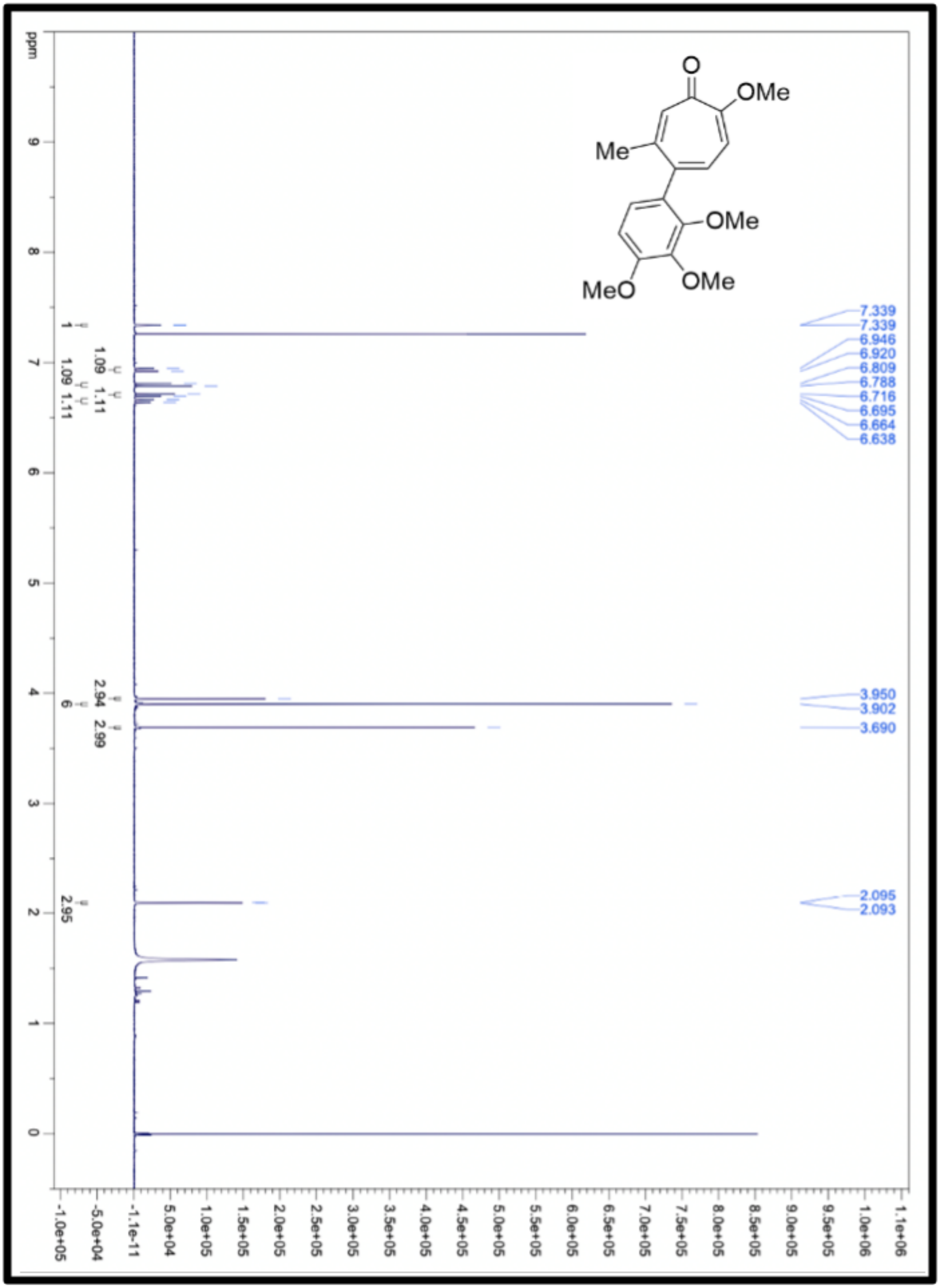

$^{13}C\{^{1}H\}$ NMR (101 MHz, $CDCl_3$) of 27

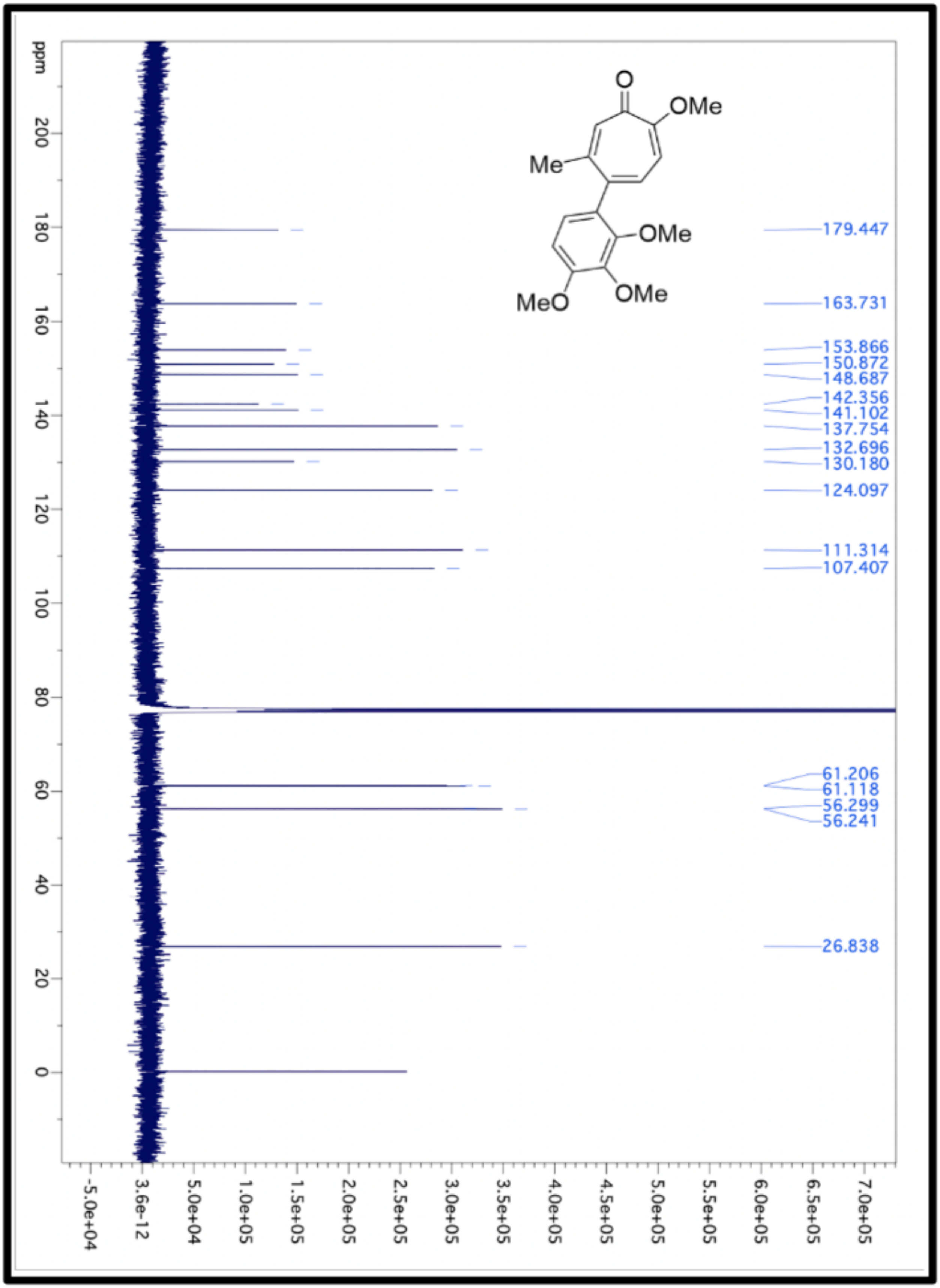

References

(1) Agyemang, N.; Murelli, R. Synthesis of 5-Hydroxy-4-Methoxy-2-Methylpyrylium Trifluoromethanesulfonate from Kojic Acid. *Org. Synth.* **2019**, *96*, 494–510. DOI: 10.15227/orgsyn.096.0494

(2) Meck, C.; Mohd, N.; Murelli, R. P. An Oxidopyrylium Cyclization/Ring-Opening Route to Polysubstituted α-Hydroxytropolones. *Org. Lett.* **2012**, *14* (23), 5988–5991. DOI: 10.10121/ol302892g

(3) Bejcek, L. P.; Garimallaprabhakaran, A. K.; Suyabatmaz, D. M.; Greer, A.; Hersh, W. H.; Greer, E. M.; Murelli, R. P. Maltol- And Allomaltol-Derived Oxidopyrylium Ylides: Methyl Substitution Pattern Kinetically Influences [5 + 3] Dimerization versus [5 + 2] Cycloaddition Reactions. *J. Org. Chem.* **2019**, 84 (22), 14670–14678.

(4) Bejcek, L. P.; Murelli, R. P. Synthesis of Aryl-Substituted 2-Methoxyphenol Derivatives from Maltol-Derived Oxidopyrylium Cycloadducts through an Acid-Mediated Ring Contraction Cascade. *Chem. Commun.* **2020**, *56* (21), 3203–3205. DOI: 10.1039/c3cc09213g.

(5) Williams, Y. D.; Meck, C.; Mohd, N.; Murelli, R. P. Triflic Acid-Mediated Rearrangements of 3-Methoxy-8-Oxabicyclo[3.2.1]Octa- 3,6-Dien-2-Ones: Synthesis of Methoxytropolones and Furans. *J. Org. Chem.* **2013**, *78* (23), 11707–11713. DOI: 10.1021/jo401617r.

(6) Meck, C.; Mohd, N.; Murelli, R. P. An Oxidopyrylium Cyclization/Ring-Opening Route to Polysubstituted α-Hydroxytropolones. *Org. Lett.* **2012**, *14* (23), 5988–5991. DOI: 10.1021/ol302892g.

(7) Rac-(1R,2S,6S,7R)-6,9-dimethoxy-3,11-dioxatricyclo[5.3.1.12,6]- dodeca-4,8-diene-10,12-dione. The Cambridge Structural Database, CCDC. DOI: 10.5517/ccdc.csd.cc22z3fm.

(8) Weingand, V.; Wurm, T.; Vethacke, V.; Dietl, M. C.; Ehjeij, D.; Rudolph, M.; Rominger, F.; Xie, J.; Hashmi, A. S. K. Intermolecular Desymmetrizing Gold-Catalyzed Yne–Yne Reaction of Push–Pull Diarylalkynes. *Chemistry – A European Journal* **2018**, *24* (15), 3725–3728. DOI: 10.1002/chem.201800360.

(9) Gangjee, A.; Namjoshi, O. A.; Keller, S. N.; Smith, C. D. 2-Amino-4-Methyl-5-Phenylethyl Substituted-7-N-Benzyl-Pyrrolo[2,3-d]Pyrimidines as Novel Antitumor Antimitotic Agents That Also Reverse Tumor Resistance. *Bioorg. Med. Chem.* **2011**, *19* (14), 4355–4365. DOI: 10.1016/j.bmc.2011.05.030.

(10) Hirsch, D. R.; Cox, G.; D'Erasmo, M. P.; Shakya, T.; Meck, C.; Mohd, N.; Wright, G. D.; Murelli, R. P. Inhibition of the ANT(2″)-Ia Resistance Enzyme and Rescue of Aminoglycoside Antibiotic Activity by Synthetic α-Hydroxytropolones. *Bioorg. Med. Chem. Lett.* **2014**, *24* (21), 4943–4947. DOI: 10.1016/j.bmcl.2014.09.037.

(11) Schiavone, D. V.; Kapkayeva, D. M.; Murelli, R. P. Investigations into a Stoichiometrically Equivalent Intermolecular Oxidopyrylium [5 + 2] Cycloaddition Reaction Leveraging 3-Hydroxy-4-Pyrone-Based Oxidopyrylium Dimers. *J. Org. Chem.* **2021**, *86* (5), 3826–3835. DOI: 10.1021/acs.joc.0c02655.

(12) D'Erasmo, M. P.; Meck, C.; Lewis, C. A.; Murelli, R. P. Discovery and Development of a Three-Component Oxidopyrylium [5 + 2] Cycloaddition. *J. Org. Chem.* **2016**, *81* (9), 3744–3751. DOI: 10.1021/acs.joc.6b00394.

(13) Hamel, E.; Mackay, M. E.; Collis, M. P.; Lambert, J. N.; Reum, M. E.; Scoble, J. A. The Palladium-Mediated Cross-Coupling of Bromotropolones with Organostannanes or Arylboronic Acids: Applications to the Synthesis of Natural Products and Natural Product Analogs. *Aust. J. Chem.* **1991**, *44* (5), 705–728. DOI: 10.1071/ch9910705.

(14) Janik, M. E.; Bane, S. L. Synthesis and Antimicrotubule Activity of Combretatropone Derivatives. *Bioorg. Med. Chem.* **2002**, *10* (6), 1895–1903. DOI: 10.1016/s0968-0896(02)00052-4.

(15) Winter, N.; Rupcic, Z.; Stadler, M.; Trauner, D. Synthesis and Biological Evaluation of (±)-Hippolachnin and Analogs. *J. Antibiot.* **2019**, *72* (6), 375–383. DOI: 10.1038/s41429-019-0176-x.
